# Cognitive Outcomes of Young Children After Prenatal Exposure to Medications for Opioid Use Disorder: A Systematic Review and Meta-analysis

**DOI:** 10.1001/jamanetworkopen.2020.1195

**Published:** 2020-03-18

**Authors:** Leah F. Nelson, Victoria K. Yocum, Keisha D. Patel, Fares Qeadan, Andrew Hsi, Sherry Weitzen

**Affiliations:** 1 Addiction Medicine Fellowship Program, Department of Family and Community Medicine, University of New Mexico, Albuquerque; 2 Honors College, University of New Mexico, Albuquerque; 3 Combined BA/MD Program, University of New Mexico, Albuquerque; 4 Division of Public Health, Department of Family and Preventive Medicine, University of Utah, Salt Lake City; 5 Department of Family and Community Medicine, University of New Mexico, Albuquerque

## Abstract

**Importance:**

The number of children with prenatal opioid exposure to medication for addiction treatment (MAT) with methadone and buprenorphine for maternal opioid use disorder is increasing, but the associations of this exposure with cognitive outcomes are not well understood.

**Objective:**

To examine the strength and consistency of findings in the medical literature regarding the association of prenatal exposure to MAT with early childhood cognitive development, particularly when accounting for variables outside MAT exposure.

**Data Sources:**

A search strategy obtained publications from PubMed, CINAHL, PsycINFO, Web of Science, and Embase from January 1972 to June 2019. Reference lists from identified articles were searched.

**Study Selection:**

Inclusion criteria were cohort studies, studies including children aged 1 to 60 months with at least 2 months of prenatal MAT exposure, studies using standardized direct-observation testing scales, and studies reporting means and SDs. Case reports, case series, historical controls, and reviews were excluded.

**Data Extraction and Synthesis:**

Two authors independently selected studies for inclusion, extracted data, and assessed study quality. Data extracted included demographic characteristics, covariates, sources of bias, and effect estimates. Meta-analysis was performed using random-effects models. This study was conducted according to the Meta-analysis of Observational Studies in Epidemiology (MOOSE) guidelines and the Preferred Reporting Items for Systematic Reviews and Meta-Analysis (PRISMA) statement. Data extraction and synthesis were conducted between January 2018 and August 2019.

**Main Outcomes and Measures:**

Cognitive test scores and demographic variability between exposed and unexposed groups.

**Results:**

A total of 16 unique cohorts, described in 27 articles and including 1086 children (485 [44.7%] with MAT exposure), were included in a quantitative synthesis. On meta-analysis, MAT exposure was associated with lower cognitive development scores (pooled standardized mean difference, −0.57; 95% CI, −0.93 to −0.21; *I*^2^ = 81%). Multiple subanalyses on demographic characteristics (ie, maternal education, race/ethnicity, socioeconomic status, prenatal tobacco exposure, infant sex) were conducted. In the subanalysis of studies with comparable prenatal exposure to tobacco smoke, the association of MAT exposure with cognitive scores was no longer statistically significant and became homogeneous (standardized mean difference, −0.11; 95% CI, −0.42 to 0.20; *I*^2^ = 0%).

**Conclusions and Relevance:**

In this study, predefined subanalyses demonstrated how poor recruitment, particularly imbalances in maternal tobacco use, could contribute to a negative overall association of cognitive development test scores with prenatal MAT exposure. Promoting tobacco cessation for pregnant women with opioid use disorder should be prioritized in this high-risk population.

## Key Points

**Question:**

Is prenatal exposure to methadone or buprenorphine for treatment of opioid use disorder during pregnancy associated with differences in cognitive development in young children?

**Findings:**

This systematic review and meta-analysis of nearly 50 years of observational research, analyzing 27 studies that included 1086 children, showed an overall negative association of exposure to methadone or buprenorphine with cognitive development. However, subanalyses revealed that this outcome may be associated with imbalances in the recruitment of mothers with different socioeconomic and educational backgrounds, levels of tobacco use in pregnancy, and fetal growth characteristics.

**Meaning:**

The findings of this study suggest that poor recruitment of comparison groups could prevent conclusive determination regarding the association of prenatal exposure to methadone or buprenorphine with cognitive outcomes. Prenatal exposure to methadone or buprenorphine may have minimal direct associations when confounders, particularly tobacco use, are controlled.

## Teaser

This systematic review and meta-analysis examines the strength and consistency of findings in the medical literature regarding the association of prenatal exposure to methadone and buprenorphine with early childhood cognitive developmental, particularly when accounting for variables outside opioid exposure.

## Introduction

The effects of the opioid crisis are permeating all areas of medicine in the US, including neonatology and pediatrics. Between 2009 and 2014, the number of women diagnosed with opioid use disorder (OUD) during pregnancy quadrupled from 1.5 to 6.5 cases per 100 000 delivery hospitalizations per year.^1^ With so many mother-fetal dyads experiencing OUD, it is recommended by the American College of Obstetricians and Gynecologists that pregnant women with OUD be treated with opioid agonists.^2^ Despite benefits for both mother and fetus, some infants develop neonatal opioid withdrawal syndrome (NOWS) and require opioid medications to alleviate withdrawal symptoms.^2,3,4,5^

After the acute withdrawal phase, the long-term consequences of prenatal exposure to medication for addiction treatment (MAT) with methadone and buprenorphine are less well understood. Some research suggests intrauterine exposure to MAT is associated with detrimental developmental outcomes, including problems with motor skills, language, and attention.^6^ However, indirect associations of a disordered home environment concomitant with the mother’s substance use disorder have been theorized as a more important factor in cognitive outcomes among these children.^7^ Women with substance use disorder often have fewer economic and employment opportunities, lower educational attainment, and a history of adverse childhood experiences, all of which may influence mother-infant interactions, maternal stress levels, and early childhood development.^8,9,10,11^

Two previous meta-analyses specific to cognitive outcomes among young children after opioid exposure have been published.^6,12^ Both identified a significant negative association (ie, lower cognitive development test scores) among children with opioid exposure. Furthermore, both meta-analyses identified that the included articles were of overall poor quality and suggested that differential social, environmental, and familial risks between children with and without exposure may contribute to the observed cognitive differences. The 2019 meta-analysis by Yeoh et al^12^ performed subanalyses on recruitment of comparable socioeconomic status and found stratification lessened the magnitude of the association of opioid exposure with cognitive development. However, neither prior meta-analysis subanalyzed on other factors associated with developmental risks, such as low maternal education or employment, infant sex, or tobacco smoke exposure, all of which are independently associated with cognitive development.^13,14,15^

The goal of this meta-analysis was to determine the consistency of findings regarding the association of prenatal exposure to methadone and buprenorphine with early childhood cognitive developmental when accounting for recruitment imbalances in the included studies. To the degree possible, we quantified the associations of predefined external variables that are associated with cognitive development of children with MAT exposure. We hypothesized that these children would have the same cognitive testing scores as children with no exposure after accounting for external maternal and infant recruitment variables.

## Methods

### Inclusion and Exclusion Criteria

This systematic review and meta-analysis was conducted according to the Meta-analysis of Observational Studies in Epidemiology (MOOSE) reporting guideline^16^ and the Preferred Reporting Items for Systematic Reviews and Meta-analyses (PRISMA) reporting guideline.^17^ A review protocol was created prior to data extraction. This review was not registered. Per the Common Rule, ethical approval and informed patient consent were not required given that this study was a literature review with no direct patient contact or influence on patient care directly related to this work.

Inclusion criteria were as follows: (1) children aged 1 to 60 months at testing, (2) prenatal exposure to legally prescribed methadone or buprenorphine during at least 2 months during pregnancy, (3) at least 10 children in each group, and (4) use of a previously published and validated direct observation method for measuring cognitive development. *Cognitive development* was defined as the construction of attention, perception, memory, language, categorization skills, reasoning and decision-making, problem solving, procedural and conceptual learning, and skill acquisition.^18^

Exclusion criteria were as follows: (1) case series and case studies, (2) use of historical or population-level data for the comparison group, (3) neurological studies without correlation to standard cognitive developmental tests (eg, visual evoked potentials, saccades), (4) parent-reports, and (5) statistics other than means and SDs.

### Search Strategy and Data Extraction

One of us (L.F.N.) has prior training and experience with meta-analysis techniques. The other reviewers (V.K.Y. and K.D.P.) had advanced scientific literature review experience from undergraduate coursework and were trained on subject-specific techniques using articles not meeting inclusion criteria. Articles were identified using an electronic and hand-searching strategy. An electronic search was performed of PubMed, CINAHL, PsycINFO, and Web of Science between January 1, 1970, and June 28, 2019 (ie, 49.5 years). Embase was searched through March 30, 2018 (ie, 48.3 years). No language constraints were applied. Search terms are available in the eAppendix in the Supplement and included variations of *prenatal exposure*, *methadone*, *buprenorphine*, *child development*, and *child behavior*.

Two of us (L.F.N and V.K.Y) independently reviewed all titles and abstracts for inclusion. Studies meeting inclusion criteria were extracted by 2 independent reviewers (L.F.N., V.K.Y., or K.D.P.) and compared. Data were extracted to a standard form for observational studies based on the Cochrane Group Data Extraction Template for Included Studies.^19^ Discrepancies were resolved by consensus through referral to the original studies and, if necessary, arbitration by a third reviewer. Reference lists of included articles were screened to find other suitable studies. Email contact with authors was attempted when insufficient data, conference abstracts, or unpublished data were identified. No further data were supplied by contacted authors. A total of 11 non–English language articles were screened for inclusion by translating the abstract using Google Translate (Google) as previously described,^20^ but none met inclusion criteria.

In some cases, authors published multiple articles on the same group of children over time. Typically, each publication was a cohort study with authors repeating testing as children aged and publishing a second or third article. Essentially, this represents a longitudinal study published at discrete points. To avoid double-counting participants from these articles, a composite extraction form was made to detail which information was extracted from each study (eTable in the Supplement). This cohort merge technique provided a more comprehensive compilation of demographic factors, given that comprehensive baseline characteristics were often described only in the first study published.

### Statistical Analysis

When available in the published studies, variables considered relevant confounders, moderators, and mediators were extracted. These were selected a priori based on literature review and clinical experience. Prespecified subgroup analyses included the following: maternal race/ethnicity; education; socioeconomic status; employment; exposure to illicit substances, tobacco, and/or alcohol; and infant sex.

Heterogeneity, ie, the variation in outcomes among studies, was assessed by the *I*^2^ index and τ. The presence of publication bias was assessed informally by visual inspection of funnel plots and formally by Egger test of the intercept.

A modified Downs and Black assessment of quality was used to evaluate internal and external validity, bias, and power.^21^ Two nonmasked reviewers (L.F.N., V.K.Y., or K.D.P.) independently completed the quality assessment form, and consensus was reached as described earlier. For the cohort merge extractions, the highest quality article was used for Downs and Black analysis. No articles were excluded on quality grounds.

Data were abstracted, quantified, coded, and assembled into a Microsoft Excel version 16.32 (Microsoft Corp) database. Statistical analysis was performed using *meta*, *metafor*, and *dpylr* packages in R Studio version 1.2.1335 (R Project for Statistical Computing). Standard meta-analytic techniques for means and SDs were used with the methods presented by Harrer et al.^22^ When testing was performed at multiple ages, the most recent point was used for meta-analysis. All developmental tests were transformed to a mean of 100 with an SD of 15. Statistical methods included calculation of weighted means and SDs as well as χ^2^, *t* tests, and *z* tests for proportions. Because of significant variation in study methods and small sample sizes, random-effect models were applied using Hedges *g* statistic for effect size and a Knapp-Hartung-Sidik-Jonkman adjustment for τ. Effect size is presented as standardized mean difference (SMD). Negative SMDs represent worse performance among children with MAT exposure. A 2-tailed α < .05 was used as the threshold for statistical significance. Data extraction and synthesis were conducted between January 2018 and August 2019.

## Results

**Figure 1.**
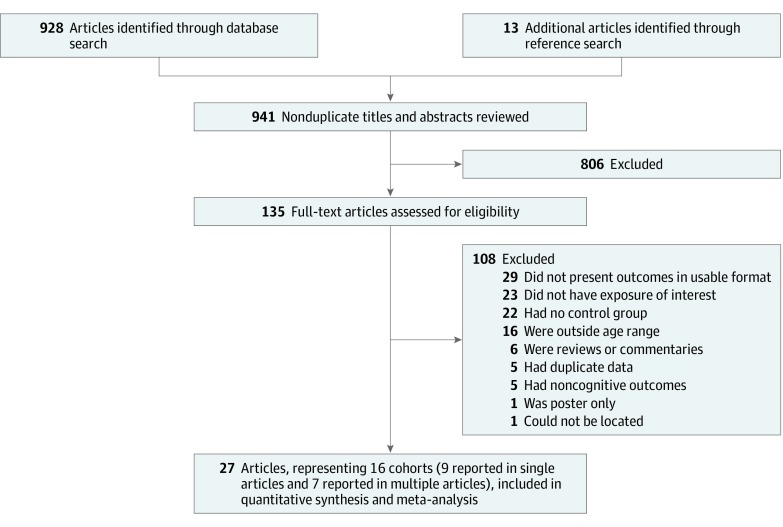
Search Strategy Flow Chart

Our literature search yielded 941 nonduplicate potential articles, of which 914 (97.1%) were excluded (Figure 1; eTable in the Supplement). A total of 27 studies met the inclusion criteria and were included in the final review, representing 16 unique cohorts of children from 6 countries.^23,24,25,26,27,28,29,30,31,32,33,34,35,36,37,38,39,40,41,42,43,44,45,46,47,48,49,50^ These cohorts included a total of 1086 children, 485 (44.7%) with exposure to methadone or buprenorphine prenatally and 601 (55.3%) with no exposure. Details of the included studies can be found in Table 1. Included cognitive tests were the Bayley Scales of Infant Development, Mental Development Index (10 cohorts [62.5%]), the Stanford-Binet Intelligence Test (1 cohort [6.3%]), the McCarthy General Cognitive Index (2 cohorts [12.5%]), Griffith Intellectual Performance (1 cohort [6.3%]), the Wechsler Preschool and Primary Scale of Intelligence–Revised (1 cohort [6.3%]), and the Revisie Amsterdamse Kinder Intelligentie Test (in Dutch; 1 cohort [6.3%]).^51,52^ In every study, the mean score on cognitive testing scales for children with MAT exposure children was within the normal range (ie, within 1 SD of the mean).

**Table 1.** General Characteristics of Included Studies and 16 Cohorts Comparing Infants With and Without Opioid Exposure

Source	Study location	Children tested, No.^a^^,^^b^	Age, mean (SD) mo^b^^,^^c^	Exposure type^b^	Cognitive test	Tester masked^d^
Bakhireva et al,^23^ 2019	Albuquerque, New Mexico	78	7 (1)	Methadone or buprenorphine	BSID MDI version 3	Yes
Bauman and Levine,^24^ 1986	Northern and Southern California	65	29 (NA)	Methadone	SBIT	Not reported
Bunikowski et al,^25^ 1998	Berlin, Germany	60	12 (NA)	Methadone	GIP	Not reported
Chasnoff et al,^26^ 1984	Chicago, Illinois	38	12 (NA)	Methadone	BSID MDI version 1	Yes
Hans cohort^27,28^ 1989, 1994	Chicago, Illinois	74	24 (NA)	Methadone	BSID MDI version 1	Yes
Hunt et al,^29^ 2008	Sydney, Australia	111	36 (NA)	Methadone	SBIT	Not reported
Kaltenbach and Finnegan,^30^ 1989	Philadelphia, Pennsylvania	44	48 (NA)	Methadone	McCarthy GCI	Not reported
Marcus cohort,^31,32^ 1984, 1986	Chicago, Illinois	40	4 (NA)	Methadone	BSID MDI version 1	Yes
Rosen and Johnson,^33^ 1988	New York, New York	40	12 (NA)	Methadone	BSID MDI version 1	Not reported
Rosen cohort,^34,35,36,37^ 1982, 1984, 1985	New York, New York	56	24 (NA)	Methadone	BSID MDI version 1	Yes
Salo et al,^38^ 2010	Helsinki, Finland	72	9.3 (2.4)	Buprenorphine	BSID MDI version 3	Yes
Strauss cohort,^39,40^ 1976, 1979	Detroit, Michigan	58	58 (4)	Methadone	McCarthy GCI	Yes
van Baar cohort,^41,42,43^ 1989, 1990, 1994	Amsterdam, the Netherlands	54	54 (NA)	Methadone	RAKIT-IQ	Not reported
Whitham,^44^ 2012	Adelaide, Australia	81	12 (NA)	Methadone or buprenorphine	BSID MDI, version 2	No
Wilson cohort,^45,46,47^ 1981, 1985, 1989	Houston, Texas	67	41 (NA)	Methadone	McCarthy GCI	Yes
Woodward cohort,^48,49,50^ 2011, 2012, 2018	Canterbury, New Zealand	148	54 (NA)	Methadone	WPPSI-R	Yes

Abbreviations: BSID MDI, Bayley Scales of Infant Development, Mental Development Index; GCI, General Cognitive Index; GIP, Griffith Intellectual Performance; NA, not available; RAKIT-IQ, Revisie Amsterdamse Kinder Intelligentie Test; SBIT, Stanford-Binet Intelligence Test; WPPSI-R, Wechsler Preschool and Primary Scale of Intelligence–Revised.

^a^ Number of children includes exposed and unexposed groups.

^b^ Number of children, age, and cognitive test are from the final point at which data were collected.

^c^ Few articles reported SDs for ages of children at the time of testing.

^d^ Tester was masked to the exposure status of the child during testing.

### Quality and Publication Bias

The mean (SD) quality of the studies was low (15.2 [4.6] of 24 points), as measured by the modified Downs and Black tool (eFigure 1 in the Supplement).^21^ Most studies had poor internal validity, particularly regarding selection bias, with recruitment of comparison mothers who were dissimilar to the mothers receiving MAT. As a whole, the included studies inadequately described the study population base, recruitment methods, children lost to follow-up, and adjustment for confounding. Assessment of loss to follow-up was performed by comparing the number of children recruited with the number evaluated at the final point for each study or cohort. Loss to follow-up was higher for children with MAT exposure, with a median (interquartile range) loss to follow-up of 39% (15%-49%) for children with exposure and 15% (7%-33%) for children without exposure. Four studies did not report sufficient baseline recruitment data to calculate losses. No studies adequately reported whether the children who were lost to follow-up differed from those who completed the study.

Visual inspection of the funnel plot (eFigure 2 in the Supplement) and Egger test of the intercept indicated no significant asymmetry (intercept, −2.3; 95% CI, −7.5 to 2.9; *P* = .40). This finding reduces the likelihood of publication bias, meaning both positive and negative findings were identified by our search strategy.

### Demographic Characteristics

Maternal and child characteristics are shown in Table 2. Compared with the nonexposed group, the MAT-exposed group had lower socioeconomic status (108 of 238 [45.3%] vs 171 of 190 [90.0%]; *P* < .001), lower educational attainment (less than high school: 82 of 241 [34.0%] vs 137 of 206 [66.5%]; *P* < .001), and a higher proportion of tobacco use (156 of 394 [39.6%] vs 314 of 353 [89.0%]; *P* < .001) and other drug use (13 of 566 [2.3%] vs 199 of 513 [38.8%]; *P* < .001) during pregnancy. Compared with infants with no MAT exposure, those with MAT exposure were more likely to be male (249 of 532 [46.8%] vs 295 of 536 [55.0%]; *P* = .03), to be born at an earlier term mean (SD) gestational age (39.3 [1.8] weeks vs 38.9 [1.9] weeks; *P* < .001), to have a lower mean (SD) birth weight (3366.6 [444.3] g vs 2966.5 [467.8] g; *P* < .001), and to have a smaller mean (SD) head circumference (34.7 [1.5] cm vs 33.4 [1.6] cm; *P* < .001). Approximately half of infants (264 of 542 [48.7%]) with MAT exposure required medical treatment for NOWS.

**Table 2.** Mother and Infant Characteristics of Medication for Addition Treatment Exposed vs Unexposed Groups

Factor	No./total No. (%)^a^		*P* value	Source
	Exposed group (n = 485)	Unexposed group (n = 601)		
Maternal factors				
Age, y^b^				
No.	299	336	.26	Bakhireva et al,^23^ Hans cohort^27,28^ Rosen and Johnson,^33^ Rosen cohort,^34,35,36,37^ Salo et al,^38^ Wilson cohort,^46^ Woodward cohort^48,49,50^
Mean (SD)	28.2 (4.7)	27.8 (4.3)		
Race/ethnicity				
Non-Hispanic white	149/409 (36.4)	174/455 (38.2)	.66^c^	Bakhireva et al,^23^ Chasnoff et al,^26^ Hans cohort,^27,28^ Marcus cohort,^31,32^ Rosen and Johnson,^33^ Rosen cohort,^34,35,36,37^ Strauss cohort,^39,40^ van Baar cohort,^41,42,43^ Whitham,^44^ Wilson cohort,^45,46,47^ Woodward cohort^48,49,50^
Black or African American	168/409 (41.1)	171/455 (37.6)		
Hispanic	57/409 (13.9)	67/455 (14.7)		
Other	32/409 (7.8)	43/455 (9.5)		
<High school education	137/206 (66.5)	82/241 (34.0)	<.001	Bakhireva et al,^23^ Chasnoff et al,^26^ Hans cohort,^27,28^ Salo et al,^38^ van Baar cohort,^41,42,43^ Whitham,^44^ Wilson cohort,^45,46,47^ Woodward cohort^48,49,50^
Employed	33/187 (19.4)	140/208 (67.3)	<.001	Bakhireva et al,^23^ Bunikowski et al,^25^ van Baar cohort,^41,42,43^ Whitham,^44^ Woodward cohort^48,49,50^
Low socioeconomic status	171/190 (90.0)	108/238 (45.3)	<.001	Bakhireva et al,^23^ Bunikowski et al,^25^ van Baar cohort,^41,42,43^ Whitham,^44^ Woodward cohort^48,49,50^
Child factors				
No. tested and recruited	384/593 (64.8)	443/592 (74.8)	.002	Bunikowski et al,^25^ Chasnoff et al,^26^ Hans cohort^27,28^ Hunt et al,^29^ Marcus cohort,^31,32^ Rosen and Johnson,^33^ Rosen cohort,^34,35,36,37^ Strauss cohort,^39,40^ van Baar cohort,^41,42,43^ Whitham,^44^ Wilson cohort,^45,46,47^ Woodward cohort^48,49,50^
Male infants	295/536 (55.0)	249/532 (46.8)	.03	Bakhireva et al,^23^ Bunikowski et al,^25^ Hans cohort^27,28^ Hunt et al,^29^ Rosen and Johnson,^33^ Rosen cohort,^34,35,36,37^ Strauss cohort,^39,40^ van Baar cohort,^41,42,43^ Whitham,^44^ Wilson cohort,^45,46,47^ Woodward cohort^48,49,50^
Gestational age, wk^b^				
No.	496	529	<.001	Bunikowski et al,^25^ Rosen and Johnson,^33^ Rosen cohort,^34,35,36,37^ Salo et al,^38^ van Baar cohort,^41,42,43^ Whitham,^44^ Wilson cohort,^45,46,47^ Woodward cohort^48,49,50^
Mean (SD)	38.9 (1.9)	39.3 (1.8)		
Birth weight, g^b^				
No.	361	457	<.001	Bakhireva et al,^23^ Chasnoff et al,^26^ Hans cohort,^27,28^ Johnson,^33^ Rosen cohort,^34,35,36,37^ Salo et al,^38^ van Baar cohort,^41,42,43^ Whitham,^44^ Wilson cohort,^45,46,47^ Woodward cohort^48,49,50^
Mean (SD)	2966.5 (467.8)	3366.6 (444.3)		
Birth head circumference, cm^b^				
No.	192	178	<.001	Chasnoff et al,^26^ Whitham,^44^ Wilson cohort,^45,46,47^ Woodward cohort^48,49,50^
Mean (SD)	33.4 (1.6)	34.7 (1.5)		
NOWS requiring treatment	264/542 (48.7)	NA	NA	Bakhireva et al,^23^ Bunikowski et al,^25^ Chasnoff et al,^26^ Kaltenbach and Finnegan,^30^ Marcus cohort,^31,32^ Rosen cohort,^34,35,36,37^ Salo et al,^38^ Whitham,^44^ Wilson cohort,^45,46,47^ Woodward cohort^48,49,50^
Prenatal exposures				
Polysubstance^d^	199/513 (38.8)	13/566 (2.3)	<.001	Bakhireva et al,^23^ Bunikowski et al,^25^ Chasnoff et al,^26^ Hans cohort^27,28^ 1989, Hunt et al,^29^ Rosen and Johnson,^33^ Rosen cohort,^34,35,36,37^ Salo et al,^38^ van Baar cohort,^41,42,43^ Whitham,^44^ Wilson cohort,^45,46,47^ Woodward cohort^48,49,50^
Alcohol	55/298 (18.4)	41/287 (14.3)	.18	Bakhireva et al,^23^ Chasnoff et al,^26^ Hans cohort,^27,28^ Rosen cohort,^34,35,36,37^ Whitham,^44^ Woodward cohort^48,49,50^
Tobacco	314/353 (89.0)	156/394 (39.6)	<.001	Bakhireva et al,^23^ Bunikowski et al,^25^ Chasnoff et al,^26^ Rosen and Johnson,^33^ Rosen cohort,^34,35,36,37^ van Baar cohort,^41,42,43^ Whitham,^44^ Wilson cohort,^45,46,47^ Woodward cohort^48,49,50^
Out-of-family care	69/292 (24)	0/386 (0)	<.001	Bunikowski et al,^25^ Hans cohort^27,28^ Hunt et al,^29^ Salo et al,^38^ van Baar cohort,^41,42,43^ Wilson cohort,^45,46,47^ Woodward cohort^48,49,50^

Abbreviation: NA, not applicable; NOWS, neonatal opioid withdrawal syndrome.

^a^ Inconsistent reporting of demographic characteristics among studies resulted in variation of denominators.

^b^ Data presented are weighted means and standard deviations.

^c^ Groupwise comparison using χ^2^ test for *P* value.

^d^ Polysubstance use was defined as use of more than 1 illicit substance during pregnancy, including illicit opioids, nonprescribed benzodiazepines, stimulants, or cannabis.

### Association of MAT With Child Cognitive Development

**Figure 2.**
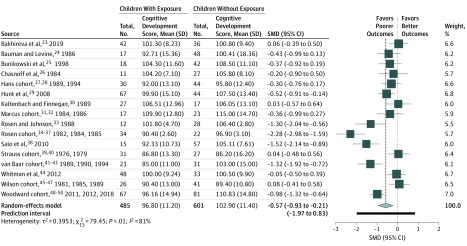
Cognitive Development Among Young Children With and Without Opioid Exposure SMD indicates standardized mean difference.

On meta-analysis of overall cognitive development (not accounting for suspected influential variables), MAT exposure was associated with statistically significantly lower cognitive test scores (pooled SMD, −0.57; 95% CI, −0.93 to −0.21). A large amount of heterogeneity between studies was apparent (*I*^2^ = 81%) (Figure 2). We evaluated the data for outliers and conduced an influence analysis using a Baujat plot.^22^ The Rosen cohort^34,35,36,37^ was identified as being very influential and a possible outlier. A sensitivity analysis excluding the Rosen cohort increased the pooled SMD to −0.46 (95% CI, −0.76 to −0.16; *I*^2^ = 74%). Because of the minimal improvement in heterogeneity, we elected to include the Rosen cohort in the analyses.

### Planned Subanalyses

Given that this study planned multiple subanalyses a priori, we set out to examine the robustness of the overall association when accounting for maternal and infant differences. First, we conducted subanalyses stratifying by whether studies recruited comparable maternal populations (Table 3). Studies were considered comparable if the exposed and unexposed groups had within 10% similarity on maternal race/ethnicity, socioeconomic status, and education level. These factors were chosen because differences in maternal education and socioeconomic status are independently associated with infant development.^11^ Race/ethnicity are social constructs, not biological characteristics, and as such are not independently associated with developmental outcomes^53^ but were included in the analysis as a proxy for whether studies recruited mothers from similar populations. As shown in Table 3, the SMD changed minimally in studies with more comparable maternal race/ethnicity, socioeconomic status, or maternal education compared with studies with less comparable characteristics (eg, education level: −0.47 [95% CI, −1.59 to 0.65] vs −0.56 [95% CI, −1.64 to 0.51]), and 95% CIs expanded across 0, becoming nonsignificant. All retained high heterogeneity (eg, education level: 87% vs 79%).

**Table 3.** Meta-analytic Comparison of More Comparable vs Less Comparable Recruitment for Maternal and Infant Confounding Variables

Factor	More comparable^a^	Less comparable^b^
**Maternal factors**		
Race/ethnicity		
SMD (95% CI)	−0.63 (−1.31 to 0.05)	−0.45 (−2.22 to 1.33)
*I*^2^, %	87	79
Individuals, No.^c^	578	156
Source	Hans cohort,^27,28^ Marcus cohort,^31,32^ Rosen cohort,^34,35,36,37^ Strauss cohort,^39,40^ Whitham,^44^ Wilson cohort,^45,46,47^ Woodward cohort^48,49,50^	Bakhireva et al,^23^ Chasnoff et al,^26^ Rosen and Johnson,^33^
Education		
SMD (95% CI)	−0.47 (−1.59 to 0.65)	−0.56 (−1.64 to 0.51)
*I*^2^, %	82	88
Individuals, No.^c^	251	361
Source	Chasnoff et al,^26^ Hans cohort,^27,28^ Strauss cohort,^39,40^ Wilson cohort,^45,46,47^	Bakhireva et al,^23^ van Baar cohort,^41,42,43^ Whitham,^44^ Woodward cohort^48,49,50^
Socioeconomic status		
SMD (95% CI)	−0.60 (−1.60 to 0.41)	−0.56 (−1.64 to 0.51)
*I*^2^, %	88	88
Individuals, No.^c^	339	361
Source	Hans cohort,^27,28^ Kaltenbach and Finnegan,^30^ Johnson,^33^ Rosen cohort,^34,35,36,37^ Strauss cohort,^39,40^ Wilson cohort,^45,46,47^	Bakhireva et al,^23^ van Baar cohort,^41,42,43^ Whitham,^44^ Woodward cohort^48,49,50^
**Child factors**		
Infant sex		
SMD (95% CI)	−0.40 (−1.35 to 0.55)	−0.84 (−1.40 to −0.28)
*I*^2^, %	87	67
Individuals, No.^c^	400	427
Source	Bakhireva et al,^23^ Bunikowski et al,^25^ Rosen cohort,^34,35,36,37^ Strauss cohort,^39,40^ Whitham,^44^ Wilson cohort,^45,46,47^	Hans cohort^27,28^ Hunt et al,^29^ Rosen and Johnson,^33^ van Baar cohort,^41,42,43^ Woodward cohort^48,49,50^
Prenatal tobacco exposure		
SMD (95% CI)	−0.11 (−0.42 to 0.20)	−1.19 (−2.00 to −0.39)
*I*^2^, %	0	87
Individuals, No.^c^	246	448
Source	Bunikowski et al,^25^ Chasnoff et al,^26^ Whitham,^44^ Wilson cohort,^45,46,47^ ^25,26,44,45,46,47^	Bakhireva et al,^23^ Johnson,^33^ Rosen cohort,^34,35,36,37^ Salo et al,^38^ van Baar cohort,^41,42,43^ Woodward cohort^48,49,50^

Abbreviation: SMD, standardized mean difference.

^a^ More comparable was defined as proportions in the exposed and unexposed groups that were similar within a study or cohort. Within 10% of exposed group value was used as threshold.

^b^ Less comparable was defined as greater than 10% and had to be explicitly reported by the authors. Studies that did not report the data for both exposed and unexposed groups were excluded.

^c^ Total number of individuals (exposed and unexposed combined).

Most studies recruited women during the prenatal period, risking an imbalance in infant characteristics, particularly sex and exposure to tobacco smoke during pregnancy. Sex imbalance can be problematic because female infants tend to score higher on standardized cognitive testing.^54^ In this subanalysis, when studies had similar proportions of male infants in the exposed and unexposed groups, the SMD improved to −0.40 (95% CI −1.35 to 0.55; *I*^2^ = 67%) and became statistically nonsignificant. As many as 85% to 90% of infants of mothers receiving MAT also have prenatal tobacco exposure^55^; however, only 4 cohorts recruited women to the comparison group who reported regular tobacco use. When these 4 cohorts were meta-analyzed, the SMD was reduced to −0.11 (95% CI, −0.42 to 0.20) with a low heterogeneity of *I^2^* = 0%. Conversely, when poorly matched studies on maternal tobacco use and infant characteristics were pooled for subanalysis, the SMD became more negative and the 95% CI was statistically significant (SMD, −1.19; 95% CI, −2.00 to −0.39).

## Discussion

Our study, as with previously published work, found that prenatal exposure to MAT was associated with a statistically significant negative difference in cognitive scores compared with those without exposure, although lower scores among these children do not necessarily indicate developmental delay. The SMD of −0.57 indicated that there is an approximately 66% chance that a child picked at random from the exposed group will have a lower score than a child picked at random from the unexposed comparison group and that there was a 76% overlap between the 2 populations.^56^ However, the high heterogeneity of 81% makes interpretation difficult. This high degree of heterogeneity remained for the majority of subanalyses, with the notable exception of tobacco smoke exposure, which had an *I*^2^ of 0%, indicating a very homogeneous sample.

While consistent with previous research, the subanalyses reported here provide evidence that the overall effect size in a meta-analysis is not a final answer to the question of interest. Conducting predefined subanalyses allowed us to demonstrate how poor study design, especially recruitment, could contribute to a negative overall finding. Tobacco use, low socioeconomic status, low educational attainment, black race, and methadone are all independently associated with poor fetal growth and birth outcomes, which can affect early childhood cognition.^13,14,15^

Although we cannot conclude whether MAT has a direct influence on the fetal brain, the well-known deleterious associations of tobacco smoke are again illustrated in this meta-analysis. Tobacco is associated with birth outcomes, early childhood development, and more severe NOWS symptoms.^13,55^ When we subanalyzed 4 cohorts with comparable tobacco smoke exposure between children exposed and unexposed to MAT, the negative association of MAT exposure with cognitive development approached zero (SMD, −0.11; 95% CI, −0.42 to 0.20), and the heterogeneity decreased to 0%. Conversely, pooling poorly comparable studies on smoking accentuated the negative association (SMD, −1.19; 95% CI, −2.00 to −0.39; *I*^2^ = 89%). This indicates 2 critical issues: first, mismatched recruitment on tobacco use in pregnancy is a likely moderator or explanatory variable for the overall negative association of MAT with cognitive development reported in previous studies, and second, intensive smoking cessation efforts should be incorporated into all opioid treatment programs for pregnant women. Previous work has shown successful contingency management strategies with quit rates of more than 30% in 12-week cessation programs for opioid-dependent pregnant women.^55^

Given that many children with prenatal opioid exposure are born into families with complicated trauma histories, low socioeconomic status, low maternal education, and experiences of racism, early childhood intervention programs should be prioritized, regardless of the presence of gross delay. In the setting of prenatal opioid exposure, home-based early intervention services have been shown to reduce child abuse and promote cognitive development.^57,58^ School-based programs for children from low-income families and/or belonging to minority groups improve school readiness, which results in lower long-term costs for special education, behavioral problems, unemployment, and later criminal behaviors.^59^ Therefore, both home-based and school-based programs should be universally available to this at-risk population.

### Limitations

This study has limitations. Meta-analyses are only as valid as the studies that contribute, and the included studies had considerable limitations with respect to recruitment of comparable unexposed groups and loss to follow-up. Our subanalyses attempted to control for imbalanced recruitment. Furthermore, included studies were observational cohorts, which are subject to many biases and can have lower internal validity compared with randomized clinical studies. Only 9 of the 16 included cohorts reported masking investigators to the participants’ exposure statuses, possibly introducing an expectancy bias. No randomized studies were identified for inclusion. Additionally, cognitive testing of infants and young children is challenging; test results have poor positive predictive value for later developmental delay.^60,61^ Therefore, the purpose of this study was not to predict developmental delay but rather to measure cognitive abilities of children with MAT exposure compared with their peers with no exposure.

In addition to problems with the internal validity of the included studies, there are limitations for this systematic review and meta-analysis. A major limitation is that only studies with means and SDs were included. We attempted to contact authors for missing data but were unsuccessful. By excluding studies with other metrics, particularly those with adjusted effect size estimates, we may have excluded data with different conclusions. Another limitation is the high heterogeneity of the overall effect and subanalyses. This is not unexpected given the long time line, variety of developmental tests, clinical factors, and children’s age range. Similar heterogeneity has been reported in previous meta-analyses of this topic.^6,12^ Next, there is a potential problem of multiple comparisons; however, this is likely limited, given that each subanalysis had a different selection of input data. A final limitation is generalizability. The included studies had a lengthy time range (ie, January 1972 to June 2019) and had a large geographic distribution (ie, North America, Europe, and Oceania), all were English-language, and most were conducted in urban settings. Therefore, it is difficult to generalize these findings to individual children in a clinical setting, particularly for rural or non–English speaking populations.

## Conclusions

In conclusion, this meta-analysis, spanning nearly 50 years of research, demonstrated that the developmental detriment reported in observational studies of children with prenatal MAT exposure could be heavily influenced by poor recruitment methods, particularly tobacco exposure. Reducing tobacco use in pregnancy and improving social equity on issues such as education, economics, employment, mental health, and access to early intervention services would likely have the greatest positive effect on children’s cognitive development after prenatal MAT exposure.

## Notes

Supplement. **eAppendix.** Electronic Database Search Strategy **eTable.** All Studies Identified by Search Strategy With Exclusion Reasons **eFigure 1.** Downs and Black Quality Assessment of All Included Cohorts **eFigure 2.** Publication Bias Funnel Plot
