# Supplementary material for: Cognitive Outcomes of Young Children After Prenatal Exposure to Medications for Opioid Use Disorder: A Systematic Review and Meta-analysis
Source: JAMA Netw Open. 2020 Mar 18;3(3):e201195. doi: 10.1001/jamanetworkopen.2020.1195 (PMC7081119; doi:10.1001/jamanetworkopen.2020.1195)
Supplement: Supplement. — eAppendix. Electronic Database Search Strategy eTable. All Studies Identified by Search Strategy With Exclusion Reasons eFigure 1. Downs and Black Quality Assessment of All Included Cohorts eFigure 2. Publication Bias Funnel Plot [file jamanetwopen-3-e201195-s001.pdf]

Supplementary Online Content

Nelson LF, Yocum VK, Patel KD, Qeadan F, Hsi A, Weitzen S. Cognitive outcomes of young children after prenatal exposure to medications for opioid use disorder: a systematic review and meta-analysis. *JAMA Netw Open*. 2020;3(3):e201195. doi:10.1001/jamanetworkopen.2020.1195

eAppendix. Electronic Database Search Strategy
eTable. All Studies Identified by Search Strategy With Exclusion Reasons
eFigure 1. Downs and Black Quality Assessment of All Included Cohorts
eFigure 2. Publication Bias Funnel Plot

This supplementary material has been provided by the authors to give readers additional information about their work.

© 2020 Nelson LF et al. *JAMA Network Open.*

eAppendix. Electronic Database Search Strategy

1. Population: humans AND infant OR child, preschool
2. Prenatal Exposure: in utero OR prenatal OR pregnan* OR (prenatal AND exposure)
3. Medication: opiate substitution treatment OR narcotics OR narcotic drugs OR opioid OR opiate OR opioid-related disorders OR analgesics OR buprenorphine OR methadone
4. Outcome: child development OR early childhood development OR child behavior OR prenatal exposure delayed effects
5. Filters: human, age 1 month - 5 years, timespan Jan. 1972- June 2019

Example: EMBASE

1. 'child development'/exp
2. 'child development and growth'/exp
3. 'child behavior'/exp
4. 'prenatal exposure'/exp
5. 1 OR #2 OR #3 OR #4
6. 'opiate substitution treatment'/exp
7. 'narcotic agent'/exp
8. 'opiate addiction'/exp
9. 'narcotic analgesic agent'/exp
10. 'buprenorphine'/exp
11. 'methadone'/exp
12. 'buprenorphine'
13. 'methadone'
14. #6 OR #7 OR #8 OR #9 OR #10 OR #11 OR #12 OR #13
15. #5 AND #14
16. 'human'/exp
17. 'preschool child'/exp
18. 'infant'/exp
19. #17 OR #18
20. #16 AND #19
21. #15 AND #20

© 2020 Nelson LF et al. *JAMA Network Open.*

eTable. All Studies Identified by Search Strategy With Exclusion Reasons

Include/Exclude (Reason)	Item Type	Publication Y	Author	Title	Publication Title	ISSN	DOI	Url	Date	Pages	Num Pages	Issue	Volume
Exposure	thesis	2018	Abbott-Egnor, Wendy	Child Sexual Abuse and Prenatal Care: Understanding Screening, Modifications, and Proper Care				http://libpro	2018		120		
Outcome	journalArticle	2007	Abdel-Latif M.E.; Bajuk B.; Lui K.; Oei J.	Short-term outcomes of infants of substance-using mothers admitted to neonatal intensive care units in New South Wales and the Australian Capital Territory	Journal of Paediatrics and Child Health	1034-4810	10.1111/j.14	http://www.	2007	127-133		3	43
Study Type	journalArticle	2013	Abdel?Latif, Mohamed E.; Oei, Julee; Craig, Fiona; Lui, Kei	Profile of infants born to drug-using mothers: A state-wide audit.	Journal of Paediatrics and Child Health	1034-4810	10.1111/j.14	http://libpro	2013-01	E80-E86		1	49
Exposure	thesis	2006	Abela, Michelle Buda	The impact of social support and other risk factors on drinking during pregnancy				http://libpro	2006		69		
Outcome	thesis	2018	Addicks, Sarah H.	Prenatal Lactation-Focused Motivational Interviewing for Enhancing Breastfeeding Initiation, Exclusivity, and Duration: Feasibility and Preliminary Outcomes				http://libpro	2018		209		
Age	journalArticle	2018	Agarwal, Pratibha; Bailey, Beth; Hall, Jesi; Devoe, Michael; Wood, David	Factors associated with maternal drug use and the severity of neonatal abstinence syndrome.	International Public Health Journal	1947-4989		http://libpro	2018-10	265-274		4	10
Age	journalArticle	2003	Akera C.; Ro S.	Medical concerns in the neonatal period	Clinics in Family Practice	1522-5720		http://www.	2003	265-292		2	5
Exposure	journalArticle	2011	Al-Tamimi, Y.; Ilett, K. F.; Paech, M. J.; O'Halloran, S. J.; Hartmann, P. E.	Estimation of infant dose and exposure to pethidine and norpethidine via breast milk following patient-controlled epidural pethidine for analgesia post caesarean delivery	International Journal of Obstetric Anesthesia	0959-289X	10.1016/j.ijoa.2010.12.00		2011-04	128-134		2	20
Study Type	journalArticle	2012	älamberov· R.	Drugs in pregnancy: The effects on mother and her progeny	Physiological Research	0862-8408		http://www.	2012	S123-S135		SUPPL. 1	61
Study Type	journalArticle	2009	Albretsen C.S.	Children of drug addicts must get a broader care	Tidsskrift for den Norske lÊgeforening : tidsskrift for praktisk medicin, ny rÊkke	0807-7096		http://www.	2009	1353		13	129
Study Type	journalArticle	1997	Alderman E.M.	Opiates.	Pediatrics in review / American Academy of Pediatrics	0191-9601		http://www.	1997	122-126		4	18
Exposure	journalArticle	1993	Alfonzo-Echeverri E.C.; Berg J.H.; Wild T.W.; Glass N.L.	Oral ketamine for pediatric outpatient dental surgery sedation.	Pediatric dentistry	0164-1263		http://www.	1993	182-185		3	15
Outcome	journalArticle	2014	Ali, Kamal; Wolff, Kim; Peacock, Janet L.; Hannam, Simon; Rafferty, Gerrard F.; Bhat, Ravindra; Greenough, Anne	Ventilatory response to hypercarbia in newborns of smoking and substance-misusing mothers.	Annals of the American Thoracic Society	2325-6621 2	10.1513/AnnalsATS.20140		2014-07	933-938		6	11
Outcome	journalArticle	2015	Aliefendio?lu D.; G¸zo?lu N.	Pain in newborn infants	Cocuk Sagligi ve Hastaliklari Dergisi	0010-0161		http://www.	2015	35-42		1	58
Outcome	journalArticle	2005	Allegaert K.; Devlieger H.; Bulckaert D.; Naulaers G.; Casaer P.; Tibboel D.	Variability in pain expression characteristics in former preterm infants	Journal of Perinatal Medicine	0300-5577	10.1515/JPM	http://www.	2005	442-448		5	33
Outcome	journalArticle	2006	Allegaert K.; Van den Anker J.N.; Debeer A.; Cossey V.; Verbesselt R.; Tibboel D.; Devlieger H.; de Hoon J.	Maturational changes in the in vivo activity of CYP3A4 in the first months of life	International Journal of Clinical Pharmacology and Therapeutics	0946-1965		http://www.	2006	303-308		7	44
Exposure	journalArticle	2004	Allotey P.; Reidpath D.D.; Elisha D.	"Social medication" and the control of children: A qualitative study of over-the-counter medication among australian children	Pediatrics	0031-4005	10.1542/ped	http://www.	2004	e378-e383		3	114
Outcome	thesis	2006	Alvarez-Rodriguez, Mary Jo	The ecological correlates of substance abuse among CPS involved women and Non -CPS involved women				http://libpro	2006		175		
Exposure	journalArticle	2017	Ancora G.; Lago P.; Garetti E.; Pirelli A.; Merazzi D.; Pierantoni L.; Ferrari F.; Faldella G.	Follow-up at the corrected age of 24 months of preterm newborns receiving continuous infusion of fentanyl for pain control during mechanical ventilation	Pain	1872-6623	10.1097/j.pa	http://www.	2017	840-845		5	158
Outcome	thesis	2015	Anderson, Courtney C.	Relationships among Emotional Availability, Touching Behavior, and Parenting Stress in Mother-Infant Dyads				http://libpro	2015		114		
Exposure	journalArticle	2008	Antela Lopez, Antonio	[Utility of atazanavir in special populations].	Enfermedades infecciosas y microbiologia clinica		10.1016/S0213-005X(08)7		2008-12	49-54			26 Suppl 17
Exposure	journalArticle	2007	Arai Y.-C.P.; Ito H.; Kandatsu N.; Kurokawa S.; Kinugasa S.; Komatsu T.	Parental presence during induction enhances the effect of oral midazolam on emergence behavior of children undergoing general anesthesia	Acta Anaesthesiologica Scandinavica	0001-5172	10.1111/j.13	http://www.	2007	858-861		7	51

© 2020 Nelson LF et al. *JAMA Network Open.*

Exposure	journalArticle	2010	Arai Y.-C.P.; Kandatsu N.; Ito H.; Sato J.; Ushida T.; Suetomi K.; Nishihara M.; Matsubara T.; Komatsu T.	Behavior of children emerging from general anesthesia correlates with their heart rate variability	Journal of Anesthesia	0913-8668	10.1007/s00	http://www	2010	317-318		2	24
Exposure	journalArticle	2008	Arai Y.-C.P.; Ueda W.; Ito H.; Wakao Y.; Matsura M.; Komatsu T.	Maternal heart rate variability just before surgery significantly correlated with emergence behavior of children undergoing general anesthesia	Paediatric Anaesthesia	1155-5645	10.1111/j.14	http://www	2008	167-171		2	18
Exposure	journalArticle	2008	Farrer L; Kranzler HR; Arias, Albert J; Gelernter, Joel; Chan, Grace; Weiss, Roger D; Brady, Kathleen T; Farrer, Lindsay; Kranzler, Henry R	Correlates of co-occurring ADHD in drug-dependent subjects: prevalence and features of substance dependence and psychiatric disorders.	Addictive Behaviors	0306-4603	10.1016/j.ad	http://libpro	2008-09	1199-1207		9	33
Outcome	journalArticle	2008	Arnold, R.; Johnson, C.; McNulty, B.; Gaisie, G.	Substantia nigra MR imaging signal changes and cardiomyopathy following prenatal exposure to cocaine and heroin.	AJNR. American journal of neuroradiology	1936-959X 0	10.3174/ajnr.A0966		2008-04	828-829		4	29
Exposure	thesis	2003	Arrindell, Janis Marie	Differential coping strategies, anxiety, depression, and symptomatology among African -American women with HIV /AIDS				http://libpro	2003		188		
Age	journalArticle	2014	Artigas V.	Management of neonatal abstinence syndrome in the newborn nursery	Nursing for women's health	1751-486X	10.1111/175	http://www	2014	509-514		6	18
Exposure	journalArticle	2010	Ashley P.F.; Parry J.; Parekh S.; Al-Chihabi M.; Ryan D.	Sedation for dental treatment of children in the primary care sector (UK)	British Dental Journal	0007-0610	10.1038/sj.b	http://www	2010	E21		11	208
Age	journalArticle	2016	Atwell K.A.; Weiss H.B.; Gibson C.; Miller R.; Corden T.E.	Neonatal abstinence syndrome and maternal substance use in Wisconsin, 2009-2014	Wisconsin Medical Journal	1098-1861		http://www	2016	287-294		6	115
Study Type	journalArticle	1982	Aylward, G. P.	Methadone outcome studies: is it more than the methadone?	The Journal of pediatrics	0022-3476 0022-3476			1982-08	214-215		2	101
Outcome	journalArticle	2002	Bada H.S.; Bauer C.R.; Shankaran S.; Lester B.; Wright L.L.; Das A.; Poole K.; Smeriglio V.L.; Finnegan L.P.; Maza P.L.	Central and autonomic system signs with in utero drug exposure	Archives of Disease in Childhood: Fetal and Neonatal Edition	1359-2998		http://www	2002	F106-F112		2	87
Outcome	journalArticle	1998	Bada H.S.; Bauer C.R.; Shankaran S.; Lester B.; Wright L.L.; Verter J.; Smeriglio V.L.; Finnegan L.P.; Maza P.L.	Central and autonomic nervous systems' signs associated with in utero exposure to cocaine/opiates	Annals of the New York Academy of Sciences	0077-8923	10.1111/j.17	http://www	1998	431-434		(Bada H.S.; E	846
Age	journalArticle	2015	Bada H.S.; Sithisarn T.; Gibson J.; Garlitz K.; Caldwell R.; Capilouto G.; Li Y.; Leggas M.; Breheny P.	Morphine versus clonidine for neonatal abstinence syndrome	Pediatrics	1098-4275	10.1542/ped	http://www	2015	e383-e391		2	135
Outcome	journalArticle	2002	Bada, H. S.; Bauer, C. R.; Shankaran, S.; Lester, B.; Wright, L. L.; Das, A.; Poole, K.; Smeriglio, V. L.; Finnegan, L. P.; Maza, P. L.	Central and autonomic system signs with in utero drug exposure.	Archives of disease in childhood. Fetal and neonatal edition	1359-2998 1359-2998			2002-09	F106-112		2	87
Exposure	journalArticle	2002	Bada, H. S.; Das, A.; Bauer, C. R.; Shankaran, S.; Lester, B.; Wright, L. L.; Verter, J.; Smeriglio, V. L.; Finnegan, L. P.; Maza, P. L.	Gestational cocaine exposure and intrauterine growth: Maternal lifestyle study	Obstetrics and Gynecology	0029-7844	10.1016/S0029-7844(02)0		2002-11	916-924		5	100
Outcome	journalArticle	2012	Bada, Henrietta S.; Bann, Carla M.; Whitaker, Toni M.; Bauer, Charles R.; Shankaran, Seetha; Lagasse, Linda; Lester, Barry M.; Hammond, Jane; Higgins, Rosemary	Protective factors can mitigate behavior problems after prenatal cocaine and other drug exposures.	Pediatrics	1098-4275 0	10.1542/peds.2011-3306		2012-12	e1479-1488		6	130
Exposure	journalArticle	2008	Charlotte; Lagasse, Linda; Bauer, Charles R.; Shankaran, Seetha; Lester, Barry M.; Higgins, Rosemary; Maza, Penelope L.	Importance of Stability of Early Living Arrangements on Behavior Outcomes of Children With and Without Prenatal Drug Exposure:	Journal of Developmental & Behavioral Pediatrics	0196-206X	10.1097/DBF	http://conte	2008-06	173-182		3	29
Study Type	journalArticle	2014	Bagley S.M.; Wachman E.M.; Holland E.; Brogly S.B.	Review of the assessment and management of neonatal abstinence syndrome	Addiction science & clinical practice	1940-0640	10.1186/194	http://www	2014	19		1	9
Exposure	journalArticle	2009	Loncar, Cynthia; LaGasse, Linda L.; Lester, Barry M.; Liu, Jing; Bauer, Charles R.; Shankaran, Seetha; Bada, Henrietta; Das, Abhik	The Effect of Parenting Stress on Child Behavior Problems in High-Risk Children with Prenatal Drug Exposure	Child psychiatry and human development	0009-398X	10.1007/s10	http://www	2009-03	73-84		1	40
Outcome	journalArticle	2006	Bajanowski, T.; Brinkmann, B.; Vennemann, M.	The San Diego definition of SIDS: practical application and comparison with the GeSID classification.	International journal of legal medicine	0937-9827 0	10.1007/s00414-005-0043		2006-11	331-336		6	120
?	journalArticle	2019	Bakhireva, Ludmila N.; Holbrook, Bradley D.; Shrestha, Shikhar; Leyva, Yuridia; Ashley, Malia; Cano, Sandra; Lowe, Jean; Stephen, Julia M.; Leeman, Lawrence	Association between prenatal opioid exposure, neonatal opioid withdrawal syndrome, and neurodevelopmental and behavioral outcomes at 5-8 months of age.	Early human development	1872-6232 0	10.1016/j.earlhumdev.201		2019-01	69-76			128
Study Type	journalArticle	2015	Baldacchino, Alex; Arbuckle, Kathleen; Petrie, Dennis J; McCowan, Colin	'Neurobehavioral consequences of chronic intrauterine opioid exposure in infants and preschool children: A systematic review and meta-analysis': Erratum.	BMC Psychiatry	1471-244X		http://libpro	6/25/15				15
Study Type	journalArticle	2014	Baldacchino, Alex; Arbuckle, Kathleen; Petrie, Dennis J.; McCowan, Colin	Neurobehavioral consequences of chronic intrauterine opioid exposure in infants and preschool children: a systematic review and meta-analysis.	BMC psychiatry	1471-244X 1	10.1186/1471-244X-14-10		4/8/14	104			14
Study Type	journalArticle	2010	Bandstra, Emmalee S.; Morrow, Connie E.; Mansoor, Elana; Accornero, Veronica H.	Prenatal drug exposure: infant and toddler outcomes.	Journal of addictive diseases	1545-0848 1	10.1080/10550881003684		2010-04	245-258		2	29

© 2020 Nelson LF et al. *JAMA Network Open.*

Outcome	thesis	2010	Bango-Sanchez, Vivian	general elf-efficacy and mother infant attachment among mothers in a residential rehabilitation facility for drug addiction and substance abuse				http://libpro	2010		143		
Exposure	journalArticle	1992	Barr E.B.; Wynn R.L.	IV sedation in pediatric dentistry: an alternative to general anesthesia.	Pediatric dentistry	0164-1263		http://www	1992	251-255		4	14
Study Type	journalArticle	1994	Barr R.G.	Bridging species: a new look and new questions.	Monographs of the Society for Research in Child Development	0037-976X		http://www	1994	82-96		1	59
Exposure	journalArticle	2006	Bartu, Anne; Sharp, Jennifer; Ludlow, Joanne; Doherty, Dorota A.	Postnatal home visiting for illicit drug-using mothers and their infants: A randomised controlled trial	Australian & New Zealand Journal of Obstetrics & Gynaecology	0004-8666	10.1111/j.1479-828X.2006		2006-10	419-426		5	46
Study Type	bookSection	1997	Batshaw, Mark L.; Conlon, Charles J.	Substance abuse: A preventable threat to development.	Children with disabilities, 4th ed.			http://libpro	1997	143-162			
Duplicate Data	journalArticle	1983	Bauman, P. S.; Dougherty, F. E.	Drug-addicted mothers' parenting and their children's development.	The International journal of the addictions	0020-773X 0020-773X			1983-04	291-302		3	18
Included	journalArticle	1986	Bauman, P. S.; Levine, S. A.	The development of children of drug addicts.	The International Journal of the Addictions	0020-773X 0020-773X			1986	849-863		8	21
Exposure	journalArticle	2013	Beaulieu, Michele J.	Oral clonidine in the management of acquired opioid dependency.	Neonatal network : NN	1539-2880 0	10.1891/0730-0832.32.6.4		2013-12	419-424		6	32
No Control Group	journalArticle	2015	Beckwith, Anna Malia; Burke, Sharon A.	Identification of Early Developmental Deficits in Infants With Prenatal Heroin, Methadone, and Other Opioid Exposure	Clinical Pediatrics	0009-9228	10.1177/00099228145495		2015-04	328-335		4	54
Study Type	journalArticle	1993	Behnke, M.; Eyler, F. D.	The consequences of prenatal substance use for the developing fetus, newborn, and young child.	The International journal of the addictions	0020-773X 0020-773X			1993-11	1341-1391		13	28
Exposure	thesis	2000	Bejarano, Anabel	Latino mother -child dyads: Attachment, psychosocial factors, mother's perception of the child's temperament, and quality of dyadic competence				http://libpro	2000		133		
Exposure	journalArticle	2005	Belcher HME; Butz AM; Wallace P; Hoon AH; Reinhardt E; Reeves SA; Pulsifer MB	Spectrum of early intervention services for children with intrauterine drug exposure.	Infants & Young Children: An Interdisciplinary Journal of Early Childhood Intervention	0896-3746		http://libpro	2005-01	15-Feb		1	18
Outcome	thesis	2016	Bell, Alicia Marie	Predicting length of treatment involvement of substance using perinatal women in an integrated inpatient healthcare program				http://libpro	2016		143		
Exposure	journalArticle	2014	Beringer R.M.; Greenwood R.; Kilpatrick N.	Development and validation of the Pediatric Anesthesia Behavior score - An objective measure of behavior during induction of anesthesia	Paediatric Anaesthesia	1155-5645	10.1111/pan	http://www	2014	196-200		2	24
Exposure	journalArticle	2006	Berner M.E.; Rimensberger P.C.; H,ppi P.S.; Pfister R.E.	National ethical directives and practical aspects of forgoing life-sustaining treatment in newborn infants in a Swiss intensive care unit	Swiss Medical Weekly	1424-7860		http://www	2006	597-602		37-38	136
Exposure	journalArticle	2010	Bernet, Vera; Latal, Beatrice; Natalucci, Giancarlo; Doell, Carsten; Ziegler, Anna; Wohlrab, Gabriele	Effect of sedation and analgesia on postoperative amplitude-integrated EEG in newborn cardiac patients.	Pediatric research	1530-0447 0	10.1203/PDR.0b013e3181		2010-06	650-655		6	67
Included	journalArticle	1994	Bernstein, V. J.; Hans, S. L.	Predicting the developmental outcome of two-year-old children born exposed to methadone: Impact of socialÄnvironmental risk factors.	Journal of Clinical Child Psychology	0047-228X	10.1207/s15	http://libpro	1994	349-359		4	23
Included	journalArticle	1984	Bernstein, V. J.; Jeremy, R. J.; Hans, S. L.; Marcus, J.	A longitudinal study of offspring born to methadone-maintained women. II. Dyadic interaction and infant behavior at 4 months.	The American journal of drug and alcohol abuse	0095-2990 0095-2990			1984	161-193		2	10
Included	journalArticle	1986	Bernstein, V. J.; Jeremy, R. J.; Marcus, J.	Mother-infant interaction in multiproblem families: finding those at risk.	Journal of the American Academy of Child Psychiatry	0002-7138 0002-7138			1986	631-640		5	25
Outcome	thesis	2000	Bertrand Finch, Jeanne Ann Marie	Mothers in recovery: Their perspectives on drug involvement and motherhood				http://libpro	2000		305		
Study Type	journalArticle	1999	Bevot A.; Krageloh-Mann I.	Effects of maternal drug and alcohol consumption in pregnancy on the development of the child	Sucht	0939-5911		http://www	1999	302-305		5	45
Exposure	journalArticle	1990	Beyer, Judith E.; McGrath, Patrick J.; Berde, Charles B.	Discordance between self-report and behavioral pain measures in children aged 3-7 years after surgery.	Journal of Pain and Symptom Management	0885-3924	10.1016/088	http://libpro	1990-12	350-356		6	5
Exposure	journalArticle	2014	Bhalla T.; Shepherd E.; Tobias J.	Neonatal pain management	Saudi Journal of Anaesthesia	0975-3125	10.4103/165	http://www	2014	S89-S97		5	8

© 2020 Nelson LF et al. *JAMA Network Open.*

No Control Group	journalArticle	2015	Bier, J. B.; Finger, A. S.; Bier, B. A.; Johnson, T. A.; Coyle, M. G.	Growth and developmental outcome of infants with in-utero exposure to methadone vs buprenorphine	Journal of Perinatology	0743-8346	10.1038/jp.2015.22		2015-08	656-659		8	35
Exposure	journalArticle	2002	Bishop-Kurylo D	Pediatric pain management in the emergency department.	Topics in Emergency Medicine	0164-2340		http://libpro	2002-03	19-30		1	24
Sample Size	thesis	2003	Bizzarro, Michael Robert	Lifetime patterns of maternal substance abuse as a predictor of child maltreatment and child developmental outcomes				http://libpro	2003		215		
Outcome	journalArticle	1994	Black M.M.; Nair P.; Kight C.; Wachtel R.; Roby P.; Schuler M.	Parenting and early development among children of drug-abusing women: Effects of home intervention	Pediatrics	0031-4005		http://www	1994	440-448		4 I	94
Outcome	journalArticle	2005	Blackburn S.	Alterations in drug handling in the pregnant woman and neonate	Journal of Perinatal and Neonatal Nursing	0893-2190		http://www	2005	14-Dec		1	19
Exposure	journalArticle	1984	Blair V.W.; Hollenbeck A.R.; Smith R.F.; Scanlon J.W.	Neonatal preference for visual patterns: Modification by prenatal anesthetic exposure?	Developmental Medicine and Child Neurology	0012-1622		http://www	1984	476-483		4	26
Outcome	thesis	2010	Blakey, Joan Marie	Struggle for custody: The salience of trauma among African American women navigating substance abuse treatment and child protection				http://libpro	2010		317		
Outcome	journalArticle	1994	Blass E.M.; Ciaramitaro V.	A new look at some old mechanisms in human newborns: taste and tactile determinants of state, affect, and action.	Monographs of the Society for Research in Child Development	0037-976X		http://www	1994	I-V, 1-81		1	59
Exposure	journalArticle	2002	Blazys D.	An informal discussion of emergency nurses' current clinical practice: What's new and what works	Journal of Emergency Nursing	0099-1767	10.1067/me	http://www	2002	549-551		6	28
Exposure	thesis	2008	Blevins, Wendy M.	Factors related to the outcomes of a residential substance abuse treatment program for women				http://libpro	2008		119		
Exposure	journalArticle	1994	Boccia M.L.; Laudenslager M.L.; Reite M.L.	Intrinsic and extrinsic factors affect infant responses to maternal separation	Psychiatry	0033-2747		http://www	1994	43-50		1	57
Exposure	journalArticle	1985	Bohlin, A. B.; Larsson, G.; Tunell, R.	[Withdrawal symptoms and behavioral disorders in children of mothers taking heroin/methadone during pregnancy]. Swedish	Lakartidningen	0023-7205	0023-7205		8/7/85	2697-2700		32-33	82
Exposure	thesis	1997	Bombardier, Cynthia Lee	Prenatal exposure to cocaine and other substances: Its effect on newborn behavior and subsequent attachment behavior				http://libpro	1997		130		
Exposure	journalArticle	2009	Bong C.L.; Ng A.S.B.	Evaluation of emergence delirium in Asian children using the Pediatric Anesthesia Emergence Delirium Scale	Paediatric Anaesthesia	1155-5645	10.1111/j.14	http://www	2009	593-600		6	19
Outcome	journalArticle	2010	Borelli, Jessica L.; Luthar, Suniya S.; Suchman, Nancy E.	Discrepancies in perceptions of maternal aggression: Implications for children of methadone-maintained mothers.	American Journal of Orthopsychiatry	0002-9432	10.1111/j.19	http://libpro	2010-07	412-421		3	80
Exposure	journalArticle	2014	Bortone, Luciano; Bertolizio, Gianluca; Engelhardt, Thomas; Frawley, Geoff; Somaini, Marta; Ingelmo, Pablo M.	The effect of fentanyl and clonidine on early postoperative negative behavior in children: a double-blind placebo controlled trial.	Paediatric anaesthesia	1460-9592 1	10.1111/pan.12388		2014-06	614-619		6	24
Exposure	journalArticle	1989	Bosshard, J. P.; Schwander, D.; Peters, G.	[Oral preanesthetic medication in children].	Revue medicale de la Suisse romande	0035-3655	0035-3655		1989-08	617-627		8	109
Exposure	journalArticle	2003	Bouwmeester N.J.; van den Anker J.N.; Hop W.C.J.; Anand K.J.S.; Tibboel D.	Age- and therapy-related effects on morphine requirements and plasma concentrations of morphine and its metabolites in postoperative infants	British Journal of Anaesthesia	0007-0912	10.1093/bja/	http://www	2003	642-652		5	90
Exposure	thesis	2006	Brachet, Tanguy Jean	Essays on the effects of maternal smoking				http://libpro	2006		103		
Study Type	journalArticle	2017	Brandt L.; Finnegan L.P.	Neonatal abstinence syndrome: Where are we, and where do we go from here?	Current Opinion in Psychiatry	1473-6578	10.1097/YCO	http://www	2017	268-274		4	30
Study Type	journalArticle	2016	Brandt L.; Swoboda P.; Fischer G.; Unger A.	Monitoring neonatal abstinence syndrome in buprenorphine-exposed in vitro fertilization twins: A case study	Substance abuse	1547-0164		http://www	2016	501-506		4	37
Study Type	journalArticle	1987	Braude M.C.; Szeto H.H.; Kuhn C.M.	Perinatal effects of drugs of abuse	Federation Proceedings	0014-9446		http://www	1987	2446-2453		7	46
Outcome	thesis	1999	Brewster, Frederick Bosley	Women in treatment for substance abuse: The effects of gender -sensitive services				http://libpro	1999		193		

© 2020 Nelson LF et al. *JAMA Network Open.*

Outcome	thesis	2006	Broder, Sally Anne	Resilience as a factor in long -term recovery from opiate addiction				http://libpro	2006		194		
Study Type	journalArticle	1994	Brooks-Gunn, J.; McCarton, C.; Hawley, T.	Effects of in utero drug exposure on children's development. Review and recommendations.	Archives of pediatrics & adolescent medicine	1072-4710 1072-4710			1994-01	33-39		1	148
Exposure	journalArticle	2006	Brown K.A.; LaferriËre A.; Lakheeram I.; Moss I.R.	Recurrent hypoxemia in children is associated with increased analgesic sensitivity to opiates	Anesthesiology	0003-3022	10.1097/000	http://www	2006	665-669		4	105
Exposure	thesis	2010	Brown, Eless D.	PRAMS evaluation 2001-2005 in Florida, Maryland, and North Carolina: Screening, treatment, and referral to services for women exposed to violence during pregnancy				http://libpro	2010		181		
Outcome	thesis	2016	Brown, Samantha M.	A Mindfulness-Based Intervention to Improve Family Functioning among Child Welfare-Involved Families with Substance Use				http://libpro	2016		131		
Outcome	thesis	2012	Brown, Suzanne	The impact of bonding history and social networks on parenting competence among mothers with substance dependence or co-occurring disorders				http://libpro	2012		169		
Outcome	thesis	2004	Brownstein-Evans, Carol	Reaffirming motherhood: Mothers, substance use, and recovery				http://libpro	2004		268		
Included	journalArticle	1998	Bunikowski R.; Grimmer I.; Heiser A.; Metze B.; Sch‰fer A.; Obladen M.	Neurodevelopmental outcome after prenatal exposure to opiates	European Journal of Pediatrics	0340-6199	10.1007/s00	http://www	1998	724-730		9	157
Outcome	thesis	1995	Burgos-Ocasio, Hilda	Social workers' attitudes toward drug-addicted mothers and their drug-exposed children				http://libpro	1995		175		
Study Type	journalArticle	1996	Burns MS; Stagg V; Saitz C; Amadi N	Innovations in practice. Intervention for infants and toddlers exposed to methadone in utero: three case studies.	Infants & Young Children: An Interdisciplinary Journal of Early Childhood Intervention	0896-3746		http://libpro	1996-07	75-88		1	9
Age	journalArticle	1996	Burns, E. Claire; O'Driscoll, Margaret; Wason, Gem	The health and development of children whose mothers are on methadone maintenance.	Child Abuse Review	0952-9136	10.1002/(SIC	http://libpro	1996-05	113-122		2	5
Outcome	thesis	2002	Busby-Pope, LaTanya Arlecia	A study of attachment and stress in substance -abusing and non -abusing mothers				http://libpro	2002		101		
Outcome	journalArticle	1998	Butz A.M.; Lears M.K.; O'Neil S.; Lukk P.	Home intervention for in utero drug-exposed infants.	Public health nursing (Boston, Mass.)	0737-1209		http://www	1998	307-318		5	15
Outcome	journalArticle	2001	Butz AM; Pulsifer M; Marano N; Belcher H; Lears MK; Royall R	Effectiveness of a home intervention for perceived child behavioral problems and parenting stress in children with in utero drug exposure.	Archives of Pediatrics & Adolescent Medicine	1072-4710		http://libpro	2001	1029-1037		9	155
Exposure	journalArticle	2005	Butz AM; Pulsifer MB; Belcher HME; Leppert M; Donithan M; Zeger S	Infant head growth and cognitive status at 36 months in children with in-utero drug exposure.	Journal of Child & Adolescent Substance Abuse	1067-828X		http://libpro	2005	15-39		4	14
Outcome	journalArticle	1998	Butz, Arlene M.; Lears, Mary Kathleen; O'Neil, Siobhan	Home intervention for in utero drug-exposed infants.	Public Health Nursing	0737-1209	10.1111/j.15	http://libpro	1998-10	307-318		5	15
Outcome	journalArticle	2006	Byers J.F.; Lowman L.B.; Francis J.; Kaigle L.; Lutz N.H.; Waddell T.; Diaz A.L.	A quasi-experimental trial on individualized, developmentally supportive family-centered care.	Journal of obstetric, gynecologic, and neonatal nursing : JOGNN / NAACOG	0884-2175		http://www	2006	105-115		1	35
Outcome	thesis	2002	Caldwell, Barbara Ann	Impact of life events, trauma, interpersonal conflict and substance abuse on pregnancy outcomes of inner city women				http://libpro	2002		186		
Exposure	journalArticle	1998	Campbell R.L.; Ross G.A.; Campbell J.R.; Mourino A.P.	Comparison of oral chloral hydrate with intramuscular ketamine, meperidine, and promethazine for pediatric sedation--preliminary report.	Anesthesia progress	0003-3006		http://www	1998	46-50		2	45
Exposure	thesis	2003	Campbell, Cynthia Im	An organizational analysis of access to female -sensitive treatment services in outpatient substance abuse treatment				http://libpro	2003		346		
Exposure	journalArticle	2017	Cano S.; Garrison L.; Lowe J.; Leeman L.; Rayburn W.F.; Stephen J.M.; Bakhireva L.N.	Neurodevelopmental indices of prenatal alcohol exposure observed in 6-month old infants	Alcoholism: Clinical and Experimental Research	1530-0277	10.1111/ace	http://www	2017	58A		(Cano S.; Ga	41
Study Type	thesis	2008	Capaldi, Lindsey	The relationship between parental substance abuse and the effects on young children				http://digita	2008				
Outcome	thesis	2008	Capstick, Carrie C.	functioning: A correlational study of executive and reflective capacities and the related contributions of substance abuse and depression				http://libpro	2008		106		

© 2020 Nelson LF et al. *JAMA Network Open.*

Outcome	thesis	2014	Cardaci, Regina	"If she can do it, so can I" An ethnograpy of a supportive living environment for women in the criminal justice system and their children				http://libpro	2014		233		
Outcome	journalArticle	1997	Carnevale, F. A.; Ducharme, C.	Adverse reactions to the withdrawal of opioids and benzodiazepines in paediatric intensive care.	Intensive & critical care nursing	0964-3397 0964-3397			1997-08	181-188		4	13
Outcome	thesis	2009	Carpenter, Tracy	Recovering women: Intersectional approaches to African American addiction				http://libpro	2009		543		
Study Type	journalArticle	1993	Casado Flores J.; Bano Rodrigo A.; Lirio Casero J.; Solera Oliva R.	Children of heroin addicted parents: A study of 119 cases	Anales Espanoles de Pediatria	0302-4342		http://www	1993	125-131		2	39
Outcome	journalArticle	1989	Chambers H.M.; Haslam R.R.	Maternal narcotic abuse and neonatal thrombocytosis.	Archives of disease in childhood	1468-2044		http://www	1989	426		3	64
Outcome	thesis	2006	Chambers, Angelina N.	Maternal -infant bonding and attachment in incarcerated postpartum women				http://libpro	2006		250		
Exposure	journalArticle	1986	Chasnoff I.J.; Burns K.A.; Burns W.J.; Schnoll S.H.	Prenatal drug exposure: Effects on neonatal and infant growth and development	Neurobehavioral Toxicology and Teratology	0275-1380		http://www	1986	357-362		4	8
Included	journalArticle	1984	Chasnoff I.J.; Schnoll S.H.; Burns W.J.; Burns K.	Maternal nonnarcotic substance abuse during pregnancy: Effects on infant development	Neurobehavioral Toxicology and Teratology	0275-1380		http://www	1984	277-280		4	6
Exposure	journalArticle	1985	Chasnoff, I. J.	Effects of maternal narcotic vs. nonnarcotic addiction on neonatal neurobehavior and infant development.	NIDA research monograph	1046-9516 1046-9516			1985	84-95			59
Outcome	journalArticle	2011	Chaworth-Musters T.; Fernandez E.; Alimenti A.; Maan E.; CÙtÈ H.; Money D.; Forbes J.	Adverse health outcomes in HIV exposed uninfected children (HEU) in British Columbia - Cihr team grant in HIV therapy and aging (Carma)	Canadian Journal of Infectious Diseases and Medical Microbiology	1712-9532		http://www	2011	12B		(Chaworth- 1	22
Exposure	journalArticle	2015	Chen X.; Wan Y.; Wen K.; Liang T.; Lin T.; Li P.	Perioperative anesthetic exposure and the neurodevelopmental status of 1 year old baby underwent neonatal cardiac surgery	Zhong nan da xue xue bao. Yi xue ban = Journal of Central South University. Medical sciences	1672-7347	10.11817/j.is	http://www	2015	1234-1238		11	40
Exposure	thesis	1998	Chew, Rebekah J.	Development, attachment and play in drug-exposed children of multiple foster care placements.				http://libpro	1998-12		3090		
Exposure	journalArticle	2007	Chiriboga C.A.; Kuhn L.; Wasserman G.A.	Prenatal cocaine exposures and dose-related cocaine effects on infant tone and behavior	Neurotoxicology and Teratology	0892-0362	10.1016/j.nt	http://www	2007	323-330		3	29
Exposure	journalArticle	2009	Cho JE; Kim JY; Hong JY; Kil HK	The addition of fentanyl to 1.5 mg/ml ropivacaine has no advantage for paediatric epidural analgesia.	Acta Anaesthesiologica Scandinavica	0001-5172	10.1111/j.13	http://libpro	2009-09	1084-1087		8	53
Outcome	thesis	2006	Choi, Sam	abusing mothers with co -occurring problems in child welfare: A study of service effectiveness with a randomized trial				http://libpro	2006		158		
Age	journalArticle	2004	Choo R.E.; Huestis M.A.; Schroeder J.R.; Shin A.S.; Jones H.E.	Neonatal abstinence syndrome in methadone-exposed infants is altered by level of prenatal tobacco exposure	Drug and Alcohol Dependence	0376-8716	10.1016/j.dr	http://www	2004	253-260		3	75
Exposure	journalArticle	2005	Chowdhury J.; Vargas K.G.	Comparison of chloral hydrate, meperidine, and hydroxyzine to midazolam regimens for oral sedation of pediatric dental patients.	Pediatric dentistry	0164-1263		http://www	2005	191-197		3	27
Exposure	journalArticle	1993	Christensen E.	Children in families with alcohol and drug problems	Ugeskrift for laeger	0041-5782		http://www	1993	2356-2357		30	155
Outcome	thesis	2000	Christenson V	Registered nurses' beliefs and perceptions regarding opioid analgesic decision-making for hospitalized children in pain.				http://libpro	2000-01		141 p		
Outcome	thesis	2013	Church, Cara	Exploring attachment and defense style effects on the self-reflective ability among individuals with addictive disorders				http://libpro	2013		260		
Study Type	journalArticle	2015	Clark, Lisa; Rohan, Annie	Identifying and assessing the substance-exposed infant.	MCN: The American Journal of Maternal/Child Nursing	0361-929X		http://libpro	2015-03	87-95		2	40
Study Type	journalArticle	2010	Cleary B.J.; Donnelly J.; Strawbridge J.; Gallagher P.J.; Fahey T.; Clarke M.; Murphy D.J.	Methadone dose and neonatal abstinence syndrome-systematic review and meta-analysis.	Addiction (Abingdon, England)	1360-0443		http://www	2010	2071-2084		12	105
Study Type	journalArticle	2019	Clemans-Cope, Lisa; Lynch, Victoria; Howell, Embry; Hill, Ian; Holla, Nikhil; Morgan, Justin; Johnson, Paul; Cross-Barnet, Caitlin; Thompson, J. Alice	Pregnant women with opioid use disorder and their infants in three state Medicaid programs in 2013-2016.	Drug and alcohol dependence	1879-0046 0	10.1016/j.drugalcdep.201		2/1/19	156-163			195

© 2020 Nelson LF et al. *JAMA Network Open.*

Outcome	journalArticle	1972	Clifton R.K.; Meyers W.J.; Solomons G.	Methodological problems in conditioning the headturning response of newborn infants.	Journal of experimental child psychology	0022-0965		http://www	1972	29-42		1	13
Outcome	thesis	2000	Coel, Rachel Anne	The social and structural contexts of sexual health status: A study of sexually transmitted infections among Chicago women				http://libpro	2000		174		
Outcome	journalArticle	2015	Cohen M.C.; Morley S.R.; Coombs R.C.	Maternal use of methadone and risk of sudden neonatal death	Acta Paediatrica, International Journal of Paediatrics	1651-2227	10.1111/apa	http://www	2015	883-887		9	104
Outcome	journalArticle	1996	Cole JG	Intervention strategies for infants with prenatal drug exposure.	Infants & Young Children: An Interdisciplinary Journal of Early Childhood Intervention	0896-3746		http://libpro	1996-01	35-39		3	8
Outcome	thesis	2016	Coleman, Jennifer	Examination of the relationship between trauma exposure and substance use severity in pregnant and recently pregnant opioid users				http://libpro	2016		115		
Outcome	thesis	2016	Collins, Aisha	The role of perceived thought control ability in the psychological well-being of African American mothers				http://libpro	2016		332		
Outcome	thesis	2009	Collins, Sara Sofia Batalha	A Multidimensional Model for the Development of Female Antisocial Personality Disorder				http://libpro	2009		106		
Outcome	journalArticle	2017	Comiskey, Catherine M.; Milnes, Jennie; Daly, Maeve	Parents who use drugs: the well-being of parent and child dyads among people receiving harm reduction interventions for opiate use.	Journal of Substance Use	1465-9891	10.1080/146	http://libpro	2017-04	206-210		2	22
Outcome	journalArticle	2013	Concheiro M.; Gonz·lez-Colmenero E.; Lendoiro E.; Concheiro-Guis·n A.; De Castro A.; Cruz-Landeira A.; LÛpez-Rivadulla M.	Alternative matrices for cocaine, heroin, and methadone in utero drug exposure detection	Therapeutic Drug Monitoring	0163-4356	10.1097/FTD	http://www	2013	502-509		4	35
Outcome	journalArticle	2017	Colmenero E.; Concheiro-Guisan A.; PeÒas-Silva P.; Macias-CortiÒa M.; Cruz-Landeira A.; LÛpez-Rivadulla M.	Bioanalysis for cocaine, opiates, methadone, and amphetamines exposure detection during pregnancy	Drug Testing and Analysis	1942-7611	10.1002/dta.	http://www	2017	898-904		6	9
Exposure	journalArticle	2017	Conner E.R.; Musser E.D.; Colpitts K.M.; Laochamroonvorapongse D.L.; Koh J.L.	Perioperative opioid administration in children with and without developmental delay undergoing outpatient dental surgery	Journal of Clinical Anesthesia	1873-4529	10.1016/j.jcl	http://www	2017	92-96		(Conner E.R.	37
Outcome	journalArticle	2013	M.; Tronick, Ed; LaGasse, Linda L.; Shankaran, Seetha; Bada, Henrietta; Bauer, Charles R.; Whitaker, Toni M.; Hammond, Jane A.; Maternal Lifestyle Study	Prenatal substance exposure: neurobiologic organization at 1 month	The Journal of Pediatrics	1097-6833	10.1016/j.jpeds.2013.04.0		2013-10	989-994.e1		4	163
Exposure	thesis	2016	Cook, Brandianne	Pregnant graduate school students' perceived facilitators and barriers to academic success and the impact on well-being				http://libpro	2016		157		
Outcome	journalArticle	2004	Cook, Michael N.; Olshan, Andrew F.; Guess, Harry A.; Savitz, David A.; Poole, Charles; Blatt, Julie; Bondy, Melissa L.; Pollock, Brad H.	Maternal medication use and neuroblastoma in offspring.	American journal of epidemiology	0002-9262 0002-9262			4/15/04	721-731		8	159
Exposure	journalArticle	2004	Cooke, C. G.; Kelley, M. L.; Fals-Stewart, W.; Golden, J.	A comparison of the psychosocial functioning of children with drug-versus alcohol-dependent fathers	American Journal of Drug and Alcohol Abuse	0095-2990	10.1081/ADA-200037530		2004	695-710		4	30
Exposure	journalArticle	2007	Coppola D.; Russo L.J.; Kwarta Jr. R.F.; Varughese R.; Schmider J.	Evaluating the postmarketing experience of risperidone use during pregnancy: Pregnancy and neonatal outcomes	Drug Safety	0114-5916		http://www	2007	247-264		3	30
Age	journalArticle	2005	Coyle M.G.; Ferguson A.; LaGasse L.; Liu J.; Lester B.	Neurobehavioral effects of treatment for opiate withdrawal	Archives of Disease in Childhood: Fetal and Neonatal Edition	1359-2998	10.1136/adc	http://www	2005	F73-F74		1	90
Age	journalArticle	2012	Coyle M.G.; Salisbury A.L.; Lester B.M.; Jones H.E.; Lin H.; Graf-Rohrmeister K.; Fischer G.	Neonatal neurobehavior effects following buprenorphine versus methadone exposure.	Addiction (Abingdon, England)	1360-0443		http://www	2012	63-73		(Coyle M.G.)	107 Suppl 1
Outcome	journalArticle	2011	Crain, Marilyn J.; Williams, Paige L.; Griner, Ray; Tassiopoulos, Katherine; Read, Jennifer S.; Mofenson, Lynne M.; Rich, Kenneth C.	Human Immunodeficiency Virus-uninfected Children With In Utero Exposure to Human Immunodeficiency Virus and Antiretroviral Medications	Pediatric Infectious Disease Journal	0891-3668	10.1097/INF.0b013e3182		2011-12	1069-1074		12	30
Study Type	journalArticle	2009	Crinnion W.J.	Maternal levels of xenobiotics that affect fetal development and childhood health	Alternative Medicine Review	1089-5159		http://www	2009	212-222		3	14
Outcome	journalArticle	2007	Crocetti M.T.; Amin D.D.; Jansson L.M.	Variability in the evaluation and management of opiate-exposed newborns in Maryland	Clinical Pediatrics	0009-9228	10.1177/000	http://www	2007	632-635		7	46
Study Type	journalArticle	1998	D'Apolito K	Substance abuse: infant and childhood outcomes.	Journal of Pediatric Nursing	0882-5963		http://libpro	1998-10	307-316		5	13
Age	journalArticle	2001	D'Apolito K.	Most prominent symptoms of withdrawal in polydrug-exposed infants.	Tennessee nurse / Tennessee Nurses Association	1055-3134		http://www	2001	20, 22, 24		4	64

Outcome	thesis	2005	D'Isernia, Kathleen	The relationship between dependence and parenting: A qualitative study of mothers in treatment				http://libpro	2005		106		
Outcome	journalArticle	2014	Dag C.; Bezgin T.; ÷zalp N.; GˆlcˌklˌAyd?n G.	Utility of bispectral index monitoring during deep sedation in pediatric dental patients	The Journal of clinical pediatric dentistry	1053-4628		http://www	2014	68-73		1	39
Outcome	journalArticle	1977	DAR, H.; SCHMIDT, R.; NITOWSKY, HM	PALMAR CREASE VARIANTS AND THEIR CLINICAL SIGNIFICANCE - STUDY OF NEWBORNS AT RISK	Pediatric Research	0031-3998	10.1203/00006450-19770		1977	103-108		2	11
Outcome	journalArticle	2004	Davidson A.; Soriano S.	Does anaesthesia harm the developing brain - Evidence or speculation?	Paediatric Anaesthesia	1155-5645	10.1046/j.14	http://www	2004	199-200		3	14
Study Type	journalArticle	1982	Davidson, Debora A.; Short, Margaret A.	Developmental effects of perinatal heroin and methadone addiction.	Physical & Occupational Therapy in Pediatrics	0194-2638	10.1300/J00	http://libpro	1982	10-Jan		4	2
Included	thesis	2011	Davie-Gray, A.	The early development and family environments of children born to mothers engaged in methadone maintenance during pregnancy.				https://ir.ca	2011				
Exposure	journalArticle	1971	Davies D.R.; Doughty A.G.	Premedication in children. A trial of intramuscular droperidol, droperidol-phenoperidine, papaveretum-hyoscine and normal saline.	British journal of anaesthesia	0007-0912		http://www	1971	65-75		1	43
Age	journalArticle	1975	Davis M.M.; Shanks B.	Neurological aspects of perinatal narcotic addiction and methadone treatment.	Addictive diseases	0094-0267		http://www	1975	213-226		2-Jan	2
Outcome	journalArticle	2006	Davis P.J.	Pharmacology for infants and children	Anesthesia and Analgesia	0003-2999		http://www	2006	37-39		SUPPL.	
Exposure	journalArticle	1992	Davis P.J.	Narcotics in pediatric anesthesia	Seminars in Anesthesia	0277-0326		http://www	1992	274-285		4	11
Age	journalArticle	1988	Davis, D. D.; Templer, D. I.	Neurobehavioral functioning in children exposed to narcotics in utero	Addictive Behaviors	0306-4603			1988	275-283		3	13
Exposure	journalArticle	1992	DAVIS, E.; FENNOY, I.; LARAQUE, D.; KANEM, N.; BROWN, G.; MITCHELL, J.	AUTISM AND DEVELOPMENTAL ABNORMALITIES IN CHILDREN WITH PERINATAL COCAINE EXPOSURE	Journal of the National Medical Association	0027-9684			1992-04	315-319		4	84
Outcome	journalArticle	2000	Dawe, S.; Harnett, P. H.; Staiger, P.; Dadds, M. R.	Parent training skills and methadone maintenance: clinical opportunities and challenges.	Drug and alcohol dependence	0376-8716 0376-8716			7/1/00	11-Jan		1	60
Age	journalArticle	2011	De Castro A.; Jones H.E.; Johnson R.E.; Gray T.R.; Shakleya D.M.; Huestis M.A.	Maternal methadone dose, placental methadone concentrations, and neonatal outcomes	Clinical Chemistry	0009-9147	10.1373/clin	http://www	2011	449-458		3	57
Age	journalArticle	1993	de Cubas, M. M.; Field, T.	Children of methadone-dependent women: developmental outcomes	The American Journal of Orthopsychiatry	0002-9432			1993-04	266-276		2	63
Exposure	journalArticle	2011	H. P.; Anand, Kanwaljeet J. S.; Duivenvoorden, Hugo J.; Weisglas-Kuperus, Nynke; Roofthooft, Daniella W. E.; Groot Jebbink, Liesbeth J. M.; Veenstra, Ravian R.;	mechanically ventilated neonates on childrenís functioning: Five-year follow-up of a randomized controlled trial.	Pain	0304-3959	10.1016/j.pa	http://libpro	2011	1391-1397		6	152
Exposure	journalArticle	1993	de Moja, C. A.	Language, personal-social abilities, and motor performance in preschool children and family characteristics of addicts in southern Italy.	Perceptual and motor skills	0031-5125 0	10.2466/pms.1993.77.2.6		1993-10	609-610		2	77
Exposure	journalArticle	1996	Delaney-Black V.; Covington C.; Ostrea E. Jr.; Romero A.; Baker D.; Tagle M.- T.; Nordstrom-Klee B.; Silvestre M.A.; Angelili M.L.; Hack C.; Long J.	Prenatal cocaine and neonatal outcome: Evaluation of dose-response relationship	Pediatrics	0031-4005		http://www	1996	735-740		4	98
Outcome	journalArticle	2012	Delano, Kaitlyn; Koren, Gideon	Emerging Biomarkers of Intrauterine Neonatal and Pediatric Exposures to Xenobiotics	Pediatric Clinics of North America	0031-3955	10.1016/j.pcl.2012.07.005		2012-10	1059-+		5	59
Exposure	journalArticle	2002	Delgado-Riley A.M.; Roger V.; Zabaleta I.A.; Adams J.A.	Mesalamine associated neutropenia in a newborn	International Pediatrics	0885-6265		http://www	2002	54-56		1	17
Outcome	thesis	2014	Denton, Lisa Kratz	Encouraging health behaviors among pregnant substance users in treatment: Does motivational enhancement therapy promote readiness to change?				http://libpro	2014		114		
Study Type	journalArticle	1986	Deren, Sherry	CHILDREN OF SUBSTANCE ABUSERS A REVIEW OF THE LITERATURE	Journal of Substance Abuse Treatment	0740-5472	10.1016/0740-5472(86)90		1986	77-94		2	3
Age	journalArticle	2015	Desai, Rishi J.; Huybrechts, Krista F.; Hernandez-Diaz, Sonia; Mogun, Helen; Patorno, Elisabetta; Kaltenbach, Karol; Kerzner, Leslie S.; Bateman, Brian T.	Exposure to prescription opioid analgesics in utero and risk of neonatal abstinence syndrome: population based cohort study.	BMJ (Clinical research ed.)	1756-1833 0959-535X			5/14/15	h2102			350

© 2020 Nelson LF et al. *JAMA Network Open.*

Age	journalArticle	2018	Devlin, Lori A.; Davis, Jonathan M.	A Practical Approach to Neonatal Opiate Withdrawal Syndrome.	American journal of perinatology	1098-8785 0	10.1055/s-0037-1608630		2018-03	324-330		4	35
Outcome	journalArticle	2009	Dick A.; Ford R.	Cholinergic and oxidative stress mechanisms in sudden infant death syndrome	Acta Paediatrica, International Journal of Paediatrics	0803-5253	10.1111/j.16	http://www	2009	1768-1775		11	98
No Control Group	journalArticle	1988	Doberczak, T. M.; Shanzer, S.; Cutler, R.; Senie, R. T.; Loucopoulos, J. A.; Kandall, S. R.	One-year follow-up of infants with abstinence-associated seizures	Archives of Neurology	0003-9942			1988-06	649-653		6	45
Outcome	journalArticle	1991	Dominguez, R.; Aguirre Vila-Coro, A.; Slopis, J. M.; Bohan, T. P.	Brain and ocular abnormalities in infants with in utero exposure to cocaine and other street drugs.	American journal of diseases of children (1960)	0002-922X 0002-922X			1991-06	688-695		6	145
Exposure	journalArticle	1985	Doring K.R.	Evaluation of an alphaprodine-hydroxyzine combination as a sedative agent in the treatment of the pediatric dental patient.	Journal of the American Dental Association (1939)	0002-8177		http://www	1985	567-576		4	111
Exposure	journalArticle	2014	Dosani F.Z.; Flaitz C.M.; Whitmire H.C.; Vance B.J.; Hill J.R.	Postdischarge events occurring after pediatric sedation for dentistry	Pediatric dentistry	1942-5473		http://www	2014	411-416		5	36
Outcome	thesis	2008	Drescher-Burke, Krista Marie	Alcohol, substance use, and birth control				http://libpro	2008		141		
Outcome	thesis	2015	Dueger, Stephanie Kirsten	An Attachment- & Mentalization-Focused Group: Experiences During One's First Pregnancy				http://libpro	2015		236		
Exposure	journalArticle	2016	Duerden, Emma G.; Guo, Ting; Dodbiba, Lorin; Chakravarty, M. Mallar; Chau, Vann; Poskitt, Kenneth J.; Synnes, Anne; Grunau, Ruth E.; Miller, Steven P.	Midazolam dose correlates with abnormal hippocampal growth and neurodevelopmental outcome in preterm infants.	Annals of Neurology	0364-5134	10.1002/ana	http://libpro	2016-04	548-559		4	79
Outcome	journalArticle	2000	Easton J.; Lamb K.	Student elective report. Paediatric sedation in the USA.	SAAD digest	0049-1160		http://www	2000	12-Mar		2	17
Exposure	journalArticle	1999	Ebrahim A.H.K.; Mathews T.	Pentazocine withdrawal in a Bahraini neonate	Bahrain Medical Bulletin	1012-8298		http://www	1999	98-99		3	21
Exposure	thesis	1996	El Khatib, Lara M.	The development of language in children prenatally exposed to drugs				http://libpro	1996		176		
Exposure	thesis	2005	El-Adham, Azza Fouad Mohammed	Childbirth pain experience recall of United States Arab immigrant women: A cross cultural comparison				http://libpro	2005		289		
Exposure	journalArticle	1973	Eldred, Carolyn A.	Judgments of right side up and figure rotation by young children.	Child Development	0009-3920	10.2307/112	http://libpro	1973-06	395-399		2	44
Exposure	journalArticle	2018	Eldridge, Whitney B.; Foster, Cherie; Wyble, Lance	Neonatal Abstinence Syndrome Due to Maternal Kratom Use.	Pediatrics	0031-4005	10.1542/ped	http://libpro	2018-12	3-Jan		6	142
No Control Group	journalArticle	2006	Elstner, Thomas; Fiala-Preinsperger, Sabine; Berger, Ernst	Developmental follow-up for children of substance dependent mothers - the Vienna Comprehensive Care Model. German	Neuropsychiatrie	0948-6259			2006	109-117		2	20
Exposure	journalArticle	1996	Epstein, R. H.; Mendel, H. G.; Witkowski, T. A.; Waters, R.; Guarniari, K. M.; Marr, A. T.; Lessin, J. B.	The safety and efficacy of oral transmucosal fentanyl citrate for preoperative sedation in young children.	Anesthesia and analgesia	0003-2999 0003-2999			1996-12	1200-1205		6	83
Exposure	journalArticle	2006	Ericsson, Elisabeth; Wadsby, Marie; Hultcrantz, Elisabeth	Pre-surgical child behavior ratings and pain management after two different techniques of tonsil surgery.	International journal of pediatric otorhinolaryngology	0165-5876 0	10.1016/j.ijporl.2006.05.0		2006-10	1749-1758		10	70
Exposure, Age	journalArticle	1989	Eriksson, M.; Billing, L.; Steneroth, G.; Zetterstrom, R.	Health and development of 8-year-old children whose mothers abused amphetamine during pregnancy.	Acta paediatrica Scandinavica	0001-656X 0001-656X			1989-11	944-949		6	78
Exposure, Age	journalArticle	1986	ERIKSSON, M.; STENEROTH, G.; ZETTERSTROM, R.	INFLUENCE OF PREGNANCY AND CHILD-REARING ON AMPHETAMINE-ADDICTED WOMEN - 5 YEAR FOLLOW-UP AFTER DELIVERY	Acta Psychiatrica Scandinavica	0001-690X	10.1111/j.1600-0447.1986		1986-06	634-641		6	73
Outcome	journalArticle	2013	Espinet S.D.; Jeong J.J.; Motz M.; Racine N.; Major D.; Pepler D.	Multimodal assessment of the mother-child relationship in a substance-exposed sample: Divergent associations with the emotional availability scales	Infant Mental Health Journal	0163-9641	10.1002/imh	http://www	2013	496-507		6	34
Outcome	thesis	2015	Evans, Elizabeth Ayn	Childhood adversity and the presence and persistence of substance use disorders over the life course among a nationally representative sample of adult women				http://libpro	2015		290		
Outcome	thesis	2010	Evans, Erica	Persistence and success among minority single mothers with substance abuse histories				http://libpro	2010		114		

© 2020 Nelson LF et al. *JAMA Network Open.*

Outcome	thesis	2006	Evans, Jessica Smith	Assessing and comparing attitudes toward addiction and methadone treatment				http://libpro	2006		154		
Study Type	journalArticle	1999	Eyler F.D.; Behnke M.	Early development of infants exposed to drugs prenatally	Clinics in Perinatology	0095-5108		http://www	1999	107-150		1	26
Exposure	thesis	1997	Fair, Cynthia DeVane	The emotional/behavioral and educational functioning of uninfected children living with maternal HIV/AIDS and substance use				http://libpro	1997		133		
Outcome	journalArticle	2018	Fang, Shao-You; Huang, Nicole; Tsay, Jen-Huoy; Chang, Su-Hui; Chen, Chuan-Yu	Excess mortality in children born to opioid-addicted parents: A national register study in Taiwan.	Drug and alcohol dependence	1879-0046 0	10.1016/j.drugalcdep.201		2/1/18	118-126			183
Outcome	journalArticle	1975	Fanshel, D.	Parental failure and consequences for children. The drug-abusing mother whose children are in foster care.	American journal of public health	0090-0036 0090-0036			1975-06	604-612		6	65
Study Type	journalArticle	2008	Farid W.O.; Dunlop S.A.; Tait R.J.; Hulse G.K.	The effects of maternally administered methadone, buprenorphine and naltrexone on offspring: Review of human and animal data	Current Neuropharmacology	1570-159X	10.2174/157	http://www	2008	125-150		2	6
Outcome	thesis	2000	Feeney, Elaine Ruth	Cost and utilization of health services for substance dependent women before and after the initiation of substance dependence treatment				http://libpro	2000		113		
Exposure	journalArticle	2004	Ferguson E; Costa N	Pediatric sedation: it's more than just the drugs!	Journal of the Association for Vascular Access	1552-8855		http://libpro	2004	73-77		2	9
Exposure	journalArticle	2009	Cano, J. M.; Gonzalez-Tome, M. I.; Guillen Martin, S.; Navarro Gomez, M.; de Jose, M. I.; Beceiro, J.; Iglesias, E.; Prieto, L.; Santos, M. J.; Martinez Guardia,	[Birth defects in a cohort of uninfected children born to HIV-infected women].	Anales de pediatria (Barcelona, Spain : 2003)	1695-4033	10.1016/j.anpedi.2008.10.		2009-03	253-64		3	70
Outcome	journalArticle	1985	Fiks, K. B.; Johnson, H. L.; Rosen, T. S.	Methadone-maintained mothers: 3-year follow-up of parental functioning.	The International journal of the addictions	0020-773X 0020-773X			1985-05	651-660		5	20
Study Type	journalArticle	2018	Fill, Mary-Margaret A.; Miller, Angela M.; Wilkinson, Rachel H.; Warren, Michael D.; Dunn, John R.; Schaffner, William; Jones, Timothy F.	Educational disabilities among children born with neonatal abstinence syndrome	Pediatrics	0031-4005	10.1542/ped	http://libpro	2018-09	9-Jan		3	142
Outcome	thesis	2000	Finch, Brian Karl	Structural determinants of infant /child health: Investigating the causes and consequences of substance use as a proximal risk factor				http://libpro	2000		175		
Outcome	thesis	1994	Fineman, Neira Rebecca	Determinants of mother-infant interaction in a sample of polysubstance abusing women				http://libpro	1994		159		
Outcome	journalArticle	2018	Finger, Brent; Jobin, Allison; Bernstein, Victor J.; Hans, Sydney	Parenting contributors to early emerging problem behaviour in children of mothers in methadone maintenance treatment	Infant and Child Development	1522-7227		http://libpro	2018-01			1	27
IBMVPKVT	journalArticle	2018	Finger, Brent; Jobin, Allison; Bernstein, Victor J.; Hans, Sydney	Parenting contributors to early emerging problem behaviour in children of mothers in methadone maintenance treatment	Infant and Child Development	1522-7219	10.1002/icd.	https://onlir	2018	e2042		1	27
Outcome	thesis	2007	Fleck, Alexine	The low and the lost: Ethics, expertise and drug use memoirs				http://libpro	2007		241		
Study Type	journalArticle	2014	Fodor, Anna; Tìm·r, J'lia; Zelena, DÛra	Behavioral effects of perinatal opioid exposure	Life Sciences	0024-3205	10.1016/j.lfs	http://www	5/28/14	8-Jan		1	104
Outcome	thesis	2014	Ford, Veronica L.	Are they content with care? A correlation study on new mothers diagnosed with substance use disorders and patient satisfaction				http://libpro	2014		124		
Outcome	thesis	2015	Forrest, Gary Miles	Attachment, Anxiety, and Depression: A Study of Women in Residential Treatment with their Children at the Susan B. Anthony Recovery Center (SBARC) (1995-2010)				http://libpro	2015		239		
Study Type	journalArticle	2004	Fortin, G.	Prenatal substance exposure	Archives De Pediatrie	0929-693X	10.1016/j.arcped.2004.03.		2004-06	525-526		6	11
Outcome	journalArticle	2012	Fournier-CharriËre E.; Tourniaire B.; Carbajal R.; Cimerman P.; Lassauge F.; Ricard C.; Reiter F.; Turquin P.; Lombart B.; Letierce A.; Falissard B.	EVENDOL, a new behavioral pain scale for children ages 0 to 7 years in the emergency department: Design and validation	Pain	0304-3959	10.1016/j.pa	http://www	2012	1573-1582		8	153
Exposure	journalArticle	1998	Frank D.A.; Augustyn M.; Zuckerman B.S.	Neonatal neurobehavioral and neuroanatomic correlates of prenatal cocaine exposure. Problems of dose and confounding	Annals of the New York Academy of Sciences	0077-8923	10.1111/j.17	http://www	1998	40-50		(Frank D.A.;	846
Exposure	journalArticle	1996	Frank D.A.; Bresnahan K.; Zuckerman B.S.	Maternal cocaine use: impact on child health and development.	Current problems in pediatrics	0045-9380		http://www	1996	57-70		2	26

© 2020 Nelson LF et al. *JAMA Network Open.*

Exposure	journalArticle	2001	Frank DA; Augustyn M; Knight WG; Pell T; Zuckerman B; Frank, D A; Augustyn, M; Knight, W G; Pell, T; Zuckerman, B	Growth, development, and behavior in early childhood following prenatal cocaine exposure: a systematic review.	JAMA: Journal of the American Medical Association	0098-7484		http://libpro	3/28/01	1613-1650		12	285
Exposure	journalArticle	1996	Frank, D. A.; Bresnahan, K.; Zuckerman, B. S.	Maternal cocaine use: impact on child health and development.	Current problems in pediatrics	0045-9380 0045-9380			1996-02	57-70		2	26
Outcome	thesis	2009	Frank, Merry L.	Therapeutic change at Project Pride: Residential substance abuse treatment from an attachment perspective				http://libpro	2009		167		
Age	thesis	2005	Fried, Maurice W.	Psychosocial and pharmacological predictors of the cognitive functioning of children prenatally exposed to illicit drugs				https://sear	2005		152		
Exposure	journalArticle	2017	Friedlander, Edwa; Feldstein, Ohad; Mankuta, David; Yaari, Maya; Harel-Gadassi, Ayelet; Ebstein, Richard P.; Yirmiya, Nurit	Social impairments among children perinatally exposed to oxytocin or oxytocin receptor antagonist.	Early Human Development	0378-3782	10.1016/j.ea	http://libpro	2017-03	13-18			106-107
Outcome	journalArticle	1999	Friedman A.S.; Kramer S.; Kreisher C.	Childhood predictors of violent behavior	Journal of Clinical Psychology	0021-9762	10.1002/(SIC	http://www	1999	843-855		7	55
Outcome	thesis	2006	Friedman-Gell, Lynne	in spiritual health, quality of life, and attachment dimensions of avoidance and anxiety in relation to program involvement and time clean				http://libpro	2006		396		
Outcome	thesis	2009	Fulcher, Gingi Maree	Motherhood and motivation for substance abuse treatment and behavior change				http://libpro	2009		170		
Outcome	thesis	2014	Fuld, Jennifer Pesha	"It's just me on my own.": Conditions affecting social support among women who use drugs				http://libpro	2014		206		
No Control Group	journalArticle	1995	Fulks, Mary-Ann L.; Harris, Susan R.	Children Exposed to Drugs in Utero: Their Scores on the Miller Assessment for Preschoolers	Canadian Journal of Occupational Therapy	0008-4174	10.1177/000	https://doi.c	4/1/95	15-Jul		1	62
Outcome	thesis	2011	Fuller, Kathryn Aimee	A Retrospective Study of Substance Use and Mental Health Disorders in a Sample of Urban American Indian and Alaska Natives				http://libpro	2011		141		
Age	journalArticle	2012	H.; Coyle, Mara G.; Kaltenbach, Karol; Badger, Gary J.; Arria, Amelia M.; Stine, Susan M.; Martin, Peter R.; Jones, HendrÈe E.	Differences in the profile of neonatal abstinence syndrome signs in methadone- versus buprenorphine-exposed neonates	Addiction	0965-2140	10.1111/j.13	http://libpro	2012-11	53-62		Suppl 1	107
Study Type	journalArticle	2016	GabrhelÌk R.; Nechansk· B.; Mrav?Ìk V.; Skurtveit S.; Lund I.O.; Handal M.	Consequences in Children Prenatally Exposed to Illicit Drugs and Opioid Maintenance Treatment Using Czech and Scandinavian Registers	Central European journal of public health	1210-7778		http://www	2016	248-251		3	24
Study Type	journalArticle	1984	Gal P; Sharpless MK	Fetal drug exposure -- behavioral teratogenesis.	Drug Intelligence & Clinical Pharmacy	0012-6578		http://libpro	1984-03	186-201		3	18
Exposure	journalArticle	2003	Gale, S.; Ozonoff, S.; Lainhart, J.	Brief report: Pitocin induction in autistic and nonautistic individuals	Journal of Autism and Developmental Disorders	0162-3257	10.1023/A:102295182947		2003-04	205-208		2	33
Exposure, Age	journalArticle	1981	Gambini E.; Lenti C.; Busacca M.; Gementi P.	Neurobehavioral evaluation of newborn infants after administration of meridine and promethazine to their mothers during labor	Rivista di neurobiologia : organo ufficiale della Societ‡ dei neurologi, neuroradiologi e neurochirurghi ospedalieri	0035-6336		http://www	1981	687-697		34	27
Exposure	journalArticle	2008	Ganesh A.; Adzick N.S.; Foster T.; Cucchiaro G.	Efficacy of addition of fentanyl to epidural bupivacaine on postoperative analgesia after thoracotomy for lungr resection in infants	Anesthesiology	0003-3022	10.1097/ALN	http://www	2008	890-894		5	109
Exposure, Outcome	journalArticle	2007	Garcia-Bournissen F.; Rokach B.; Karaskov T.; Koren G.	Methamphetamine detection in maternal and neonatal hair: Implications for fetal safety	Archives of Disease in Childhood: Fetal and Neonatal Edition	1359-2998	10.1136/adc	http://www	2007	F351-F355		5	92
Exposure	journalArticle	2012	Gargari S.S.; Fallahian M.; Haghighi L.; Hosseinnezhad-Yazdi M.; Dashti E.; Dolan K.	Maternal and neonatal complications of substance abuse in Iranian pregnant women	Acta Medica Iranica	0044-6025		http://www	2012	411-416		6	50
Exposure	journalArticle	2006	Garrido, Maria J.; Habre, Walid; Rombout, Ferdinand; Troconiz, Inaki F.	Population pharmacokinetic/pharmacodynamic modelling of the analgesic effects of tramadol in pediatrics.	Pharmaceutical research	0724-8741 0	10.1007/s11095-006-9049		2006-09	2014-2023		9	23
Exposure	thesis	2003	Gentry, Quinn Michelle	Risk in the rough: An ethnographic inquiry of how poor African -American women who smoke crack reduce their risks for HIV -infection				http://libpro	2003		240		
Outcome	thesis	2013	German, Shanna T.	Emotional Availability in Mothers and Infants in Relation to Maternal Factors of Employment, Self-Efficacy and Stress				http://libpro	2013		82		
Exposure	journalArticle	1993	Giaufre E.	Epidural and spinal anaesthesia in children	Bailliere's Clinical Anaesthesiology	0950-3501		http://www	1993	727-747		3	7

© 2020 Nelson LF et al. *JAMA Network Open.*

Outcome	journalArticle	2017	Gibson, Kelly S.; Stark, Sydney; Kumar, Deepak; Bailit, Jennifer L.	The relationship between gestational age and the severity of neonatal abstinence syndrome.	Addiction	0965-2140	10.1111/add	http://libpro	2017-04	711-716		4	112
Outcome	journalArticle	2003	Gill, A. C.; Oei, J.; Lewis, N. L.; Younan, N.; Kennedy, I.; Lui, K.	Strabismus in infants of opiate-dependent mothers.	Acta paediatrica (Oslo, Norway : 1992)	0803-5253 0803-5253			2003	379-385		3	92
Exposure	thesis	2012	Gill, Marie E.	Predictors of Drug Court Client Graduation				http://libpro	2012		154		
Exposure	thesis	2009	Gillam, Susan	Expecting to quit: An implementation evaluation of a smoking cessation intervention for pregnant and parenting women				http://libpro	2009		388		
Exposure	journalArticle	2004	Ginsberg G.; Hattis D.; Miller R.; Sonawane B.	Pediatric Pharmacokinetic Data: Implications for Environmental Risk Assessment for Children	Pediatrics	0031-4005		http://www	2004	973-983		4 II	113
Exposure	journalArticle	2018	Giordano, V.; Deindl, P.; Fuiko, R.; Unterasinger, L.; Waldhoer, T.; Cardona, F.; Berger, A.; Olischar, M.	Effect of increased opiate exposure on three years neurodevelopmental outcome in extremely preterm infants.	Early human development	1872-6232 0	10.1016/j.earlhumdev.201		2018-08	5-Jan			123
Exposure	thesis	2001	Giovanola, Sophie	Psychological differentiation, depression, and patterns of coping with stress in HIV -positive mothers				http://libpro	2001		202		
Study Type	journalArticle	2009	Glatstein M.; Finkelstein Y.; Scolnik D.	Accidental methadone ingestion in an infant: Case report and review of the literature	Pediatric Emergency Care	0749-5161	10.1097/PEC	http://www	2009	109-111		2	25
Study Type	journalArticle	2014	Glauser W.	Divide over treating opioid-addicted moms.	CMAJ : Canadian Medical Association journal = journal de l'Association medicale canadienne	1488-2329	10.1503/cma	http://www	2014	E363-364		10	186
Outcome	journalArticle	1991	Goddard, Sarah; Bennett, Gerald; Rigby, Kate	Psychological change during residence in a rehabilitation centre for female drug misusers: II. Children of drug misusers.	Drug and Alcohol Dependence	0376-8716	10.1016/037	http://libpro	1991-03	159-165		2	27
Outcome	journalArticle	2011	Goel, Nitin; Beasley, Dana; Rajkumar, Veena; Banerjee, Sujoy	Perinatal outcome of illicit substance use in pregnancy--comparative and contemporary socio-clinical profile in the UK.	European journal of pediatrics	1432-1076 0	10.1007/s00431-010-1284		2011-02	199-205		2	170
Outcome	thesis	2001	Golder, Seana Marie	Modeling women's substance use and lawbreaking behavior				http://libpro	2001		163		
Outcome	thesis	2007	Goldman, Geraldine DeLong	A study of the pre-adoption functioning and post-adoption adjustment of children who have been prenatally exposed to alcohol, tobacco and/or other drugs				http://searc	2007				
Outcome	thesis	2001	Goldsberry, Yvonne Patricia	The deterrent effect of state mandatory child abuse and neglect reporting laws on alcohol and drug use during pregnancy				http://libpro	2001		216		
Outcome	journalArticle	2000	Goldstein, R. B.; McAvay, G. J.; Nunes, E. V.; Weissman, M. M.	Maternal life history--versus gestation-focused assessment of prenatal exposure to substances of abuse.	Journal of substance abuse	0899-3289 0899-3289			2000	355-368		4	11
Outcome	journalArticle	2011	Goldwater P.N.	A perspective on SIDS pathogenesis. The hypotheses: plausibility and evidence	BMC Medicine	1741-7015	10.1186/174	http://www	2011			(Goldwater	9
Exposure	journalArticle	1996	Golub, Mari S.	Labor analgesia and infant brain development.	Pharmacology, Biochemistry and Behavior	0091-3057	10.1016/S00	http://libpro	1996-12	619-628		4	55
Outcome	journalArticle	2005	Goodman, G.; Hans, S. L.; Bernstein, V. J.	behaviors as predictors of mother and child communication at 24 months in children of methadone-maintained women	Infant Mental Health Journal	1097-0355	10.1002/imh	http://online	2005	549-569		6	26
Outcome	journalArticle	1999	Goodman, G.; Hans, S. L.; Cox, S. M.	Attachment behavior and its antecedents in offspring born to methadone-maintained women.	Journal of Clinical Child Psychology	0047-228X 0	10.1207/s15374424jccp28		1999	58-69		1	28
Outcome	thesis	1991	Goodman, Geoff	Identifying attachment patterns and their antecedents among opioid-exposed 12-month-old infants.				http://libpro	1991-11		2773		
Outcome	thesis	1995	Gorsuch, Paulette	Childhood family disruption as a cofactor of impaired self-esteem and psychopathology in substance abusing female criminals				http://libpro	1995		169		
Study Type	journalArticle	2018	Gortner, Ludwig; Meyer, Sascha	[Long-term Outcome after Prenatal Drug Exposure]. German.	Zeitschrift fur Geburtshilfe und Neonatologie	1439-1651 0	10.1055/s-0043-120452		2018-02	13-18		1	222
Outcome	journalArticle	2012	Gover A.; Brummelte S.; Synnes A.R.; Miller S.P.; Brant R.; Weinberg J.; Grunau R.E.	Single course of antenatal steroids did not alter cortisol in preterm infants up to 18 months	Acta Paediatrica, International Journal of Paediatrics	0803-5253	10.1111/j.16	http://www	2012	604-608		6	101

© 2020 Nelson LF et al. *JAMA Network Open.*

Outcome	thesis	2005	Gragnani, Cynthia T.	The role of substance abuse, change style preference, demographic variables, and turning point events on criminal behavior in an ethnically diverse population				http://libpro	2005		226		
Outcome	journalArticle	2010	Gray, Teresa R.; Choo, Robin E.; Concheiro, Marta; Williams, Erica; Elko, Andrea; Jansson, Lauren M.; Jones, HendreÈ E.; Huestis, Marilyn A.	Prenatal methadone exposure, meconium biomarker concentrations and neonatal abstinence syndrome.	Addiction	0965-2140	10.1111/j.13	http://libpro	2010-12	2151-2159		12	105
Outcome	journalArticle	2012	S.; Bann, Carla M.; Miller-Loncar, Cynthia; Twomey, Jean E.; Bursi, Charlotte; Woldt, Eunice; Nelson, Jay Ann; Fleischmann, Debra; Alexander, Barbara	Maintaining participation and momentum in longitudinal research involving high-risk families.	Journal of nursing scholarship : an official publication of Sigma Theta Tau International Honor Society of Nursing	1547-5069 1	10.1111/j.1547-5069.2012		2012-06	120-126		2	44
Outcome	thesis	2003	Green, Sherri Lynn	client outcomes: Evaluation of anti-discriminatory practice in North Carolina perinatal and maternal substance abuse programs				http://libpro	2003		186		
Outcome	journalArticle	2003	Greene CM; Goodman MH	Neonatal abstinence syndrome: strategies for care of the drug-exposed infant.	Neonatal Network	0730-0832		http://libpro	2003-07	15-60		4	22
Exposure	journalArticle	1998	Greene O.; Varghese A.; Tuamokumo F.; Ashe W.K.; Ting P.	Perinatal outcome after cocaine ± polydrug exposure	Annals of the New York Academy of Sciences	0077-8923	10.1111/j.17	http://www	1998	396-398		(Greene O.,	846
Outcome	thesis	2018	Greene, Marion S.	The Risks and Consequences of Opioid Misuse				http://libpro	2018		102		
Study Type	thesis	2008	Greeno, Elizabeth J.	Psychosocial risk factors and prevalence of alcohol consumption in pregnant women with substance abuse: A secondary data analysis				http://libpro	2008		207		
Exposure	journalArticle	2006	GrÈgoire M.-C.; Frager G.	Ensuring pain relief for children at the end of life	Pain Research and Management	1203-6765		http://www	2006	163-172		3	11
Outcome	journalArticle	1993	GREIF, GL; DRECHSLER, M.	Common issues for parents in a methadone-maintenance group	Journal of Substance Abuse Treatment	0740-5472	10.1016/0740-5472(93)90		1993-08	339-343		4	10
Outcome	thesis	2008	Gresham, Debra L.	Test of the predictive validity of the Florida supplement to the American Society of Addiction Medicine patient placement criteria				http://libpro	2008		225		
Exposure	journalArticle	2014	Grewen, Karen; Burchinal, Margaret; Vachet, Clement; Gouttard, Sylvain; Gilmore, John H.; Lin, Weili; Johns, Josephine; Elam, Mala; Gerig, Guido	Prenatal cocaine effects on brain structure in early infancy.	NeuroImage	1053-8119	10.1016/j.ne	http://libpro	11/1/14	114-123			101
Exposure	journalArticle	2015	Grewen, Karen; Salzwedel, Andrew P.; Gao, Wei	Functional connectivity disruption in neonates with prenatal marijuana exposure.	Frontiers in Human Neuroscience	1662-5161		http://libpro	11/4/15				9
Exposure	thesis	2008	Griffin, Jonyanee Boonmee	The experience of mothers of a preterm infant during the first month after the infant's hospital discharge				http://libpro	2008		180		
Exposure	journalArticle	2004	Grunau R.E.; Weinberg J.; Whitfield M.F.	Neonatal procedural pain and preterm infant cortisol response to novelty at 8 months.	Pediatrics	1098-4275		http://www	2004	e77-84		1	114
Exposure	journalArticle	2009	Grunau R.E.; Whitfield M.F.; Petrie-Thomas J.; Synnes A.R.; Cepeda I.L.; Keidar A.; Rogers M.; MacKay M.; Hubber-Richard P.; Johannesen D.	Neonatal pain, parenting stress and interaction, in relation to cognitive and motor development at 8 and 18 months in preterm infants	Pain	0304-3959	10.1016/j.pa	http://www	2009	138-146		2-Jan	143
Exposure	journalArticle	2005	Oberlander, Tim; Weinberg, Joanne; Solimano, Alfonso; Whitfield, Michael F.; Fitzgerald, Colleen; Yu, Wayne	Neonatal procedural pain exposure predicts lower cortisol and behavioral reactivity in preterm infants in the NICU.	Pain	0304-3959 0	10.1016/j.pain.2004.10.02		2005-02	293-300		3	113
Exposure	journalArticle	2004	Grunau, Ruth E.; Weinberg, Joanne; Whitfield, Michael F.	Neonatal procedural pain and preterm infant cortisol response to novelty at 8 months.	Pediatrics	1098-4275 0031-4005			2004-07	e77-84		1	114
Exposure	journalArticle	2005	Guevara-Lopez, Uriah; Covarrubias-Gomez, Alfredo; Delille-Fuentes, Ramon; Hernandez-Ortiz, Andres; Carrillo-Esper, Raul; Moyao-Garcia, Diana	[Practice guidelines for the management of acute perioperative pain].	Cirugia y cirujanos	0009-7411			2005-06	223-32		3	73
Outcome	journalArticle	2015	Gugusheff, Jessica Rose; Ong, Zhi Yi; Muhlhausler, Beverly Sara	The early origins of food preferences: targeting the critical windows of development	Faseb Journal	0892-6638	10.1096/fj.14-255976		2015-02	365-373		2	29
Exposure	thesis	1998	Gunderson, Erica Pauline	The influence of pregnancy on the weight status of women				http://libpro	1998		312		
Age	journalArticle	1994	Guo, X.; Spencer, J. W.; Suess, P. E.; Hickey, J. E.; Better, W. E.; Herning, R. I.	Cognitive brain potential alterations in boys exposed to opiates: in utero and lifestyle comparisons.	Addictive behaviors	0306-4603 0306-4603			1994-08	429-441		4	19
Study Type	journalArticle	1992	Gustavsson, Nora S.	Drug exposed infants and their mothers: Facts, myths, and needs.	Social Work in Health Care	0098-1389	10.1300/J01	http://libpro	1992	87-100		4	16

© 2020 Nelson LF et al. *JAMA Network Open.*

Exposure	journalArticle	2015	Haabrekke, Kristin J.; Siqveland, Torill; Smith, Lars; Wentzel-Larsen, Tore; Walhovd, Kristine B.; Moe, Vibeke	Mother-Child Interaction and Early Language Skills in Children Born to Mothers with Substance Abuse and Psychiatric Problems	Child Psychiatry & Human Development	0009-398X	10.1007/s10578-014-0512		2015-10	702-714		5	46
Outcome	thesis	1978	Hale, L. E.	The effects of intrauterine exposure to heroin and methadone on child motor development and learning potential.				http://libpro	1978		2132		
Exposure	journalArticle	2002	Hamers, Jan P. H.; Abu-Saad, Huda Huijer	Children's pain at home following (adeno)tonsillectomy.	European Journal of Pain	1090-3801	10.1053/euj	http://libpro	2002-05	213-219		3	6
Outcome	journalArticle	2010	Hamilton, R.; McGlone, L.; MacKinnon, J. R.; Russell, H. C.; Bradnam, M. S.; Mactier, H.	Ophthalmic, clinical and visual electrophysiological findings in children born to mothers prescribed substitute methadone in pregnancy.	The British journal of ophthalmology	1468-2079 0	10.1136/bjo.2009.169284		2010-06	696-700		6	94
Population	thesis	2009	Hammers, Dustin B.	Neuropsychological, personality, and cerebral oxygenation correlates of undergraduate poly -substance use				http://libpro	2009		145		
Study Type	journalArticle	1997	Hancock J	The passive effects of addiction: how maternal substance abuse affects the health and development of the child.	Journal of Neonatal Nursing	1355-1841		http://libpro	1997-05	14-18		3	3
Exposure	journalArticle	2011	Hand D.; Averley P.; Lyne J.; Girdler N.	Advanced paediatric conscious sedation: an alternative to dental general anaesthetic in the U.K.	SAAD digest	0049-1160		http://www	2011	24-29		(Hand D.) De	27
Exposure	journalArticle	1993	Haney K.L.; McWhorter A.G.; Seale N.S.	An assessment of the success of meperidine and promethazine sedation in medically compromised children.	ASDC journal of dentistry for children			http://www	1993	288-294		5-Apr	60
Outcome	journalArticle	1983	Hans S.L.; Marcus J.	Motoric and attentional behavior in infants of methadone-maintained women.	NIDA research monograph	1046-9516		http://www	1983	287-293		(Hans S.L.; M	43
Included	journalArticle	1989	Hans, S. L.	Developmental consequences of prenatal exposure to methadone.	Annals of the New York Academy of Sciences	0077-8923 0077-8923			1989	195-207			562
Outcome	journalArticle	1999	Hans, S. L.; Bernstein, V. J.; Henson, L. G.	The role of psychopathology in the parenting of drug-dependent women	Development and Psychopathology	0954-5794	10.1017/S0954579499002		1999	957-977		4	11
Outcome	thesis	2016	Hardin, Ashleigh Michelle	The age of intervention: Addiction, culture, and narrative during the War on Drugs				http://libpro	2016		250		
Outcome	thesis	2000	Hardy, Barbara Nell	Perceived differences in social interaction and family systems among women who are in substance abuse treatment, incarcerated and employed				http://libpro	2000		169		
Exposure	thesis	2015	Harlan Kaneaiakala, Alia	Understanding addiction as a pseudo attachment				http://libpro	2015		168		
Outcome	thesis	1995	Harmon, Michele Alicia	Reducing drug use among pregnant and parenting teens: A program evaluation and theoretical examination				http://libpro	1995		215		
Outcome	thesis	1994	Harpring, Jayme Elizabeth	Information-seeking by pregnant, drug-addicted women: An application of the sense-making approach				http://libpro	1994		276		
Study Type	journalArticle	1994	Harris S.R.; Lee M.; Fulks M.-A.	Transient dystonia in an infant with prenatal drug exposure: A case report	Pediatric Physical Therapy	0898-5669		http://www	1994	185-188		4	6
Exposure	journalArticle	2012	Harrison D.; Beggs S.; Stevens B.	Sucrose for procedural pain management in infants	Pediatrics	1098-4275	10.1542/ped	http://www	2012	918-925		5	130
Exposure	journalArticle	1991	Hasty M.F.; Vann Jr. W.F.; Dilley D.C.; Anderson J.A.	investigation of chloral hydrate, hydroxyzine pamoate, and meperidine vs. chloral hydrate and hydroxyzine pamoate.	Pediatric dentistry	0164-1263		http://www	1991	19-Oct		1	13
Exposure	journalArticle	1995	HAWLEY, TL; HALLE, TG; DRASIN, RE; THOMAS, NG	CHILDREN OF ADDICTED MOTHERS - EFFECTS OF THE CRACK EPIDEMIC ON THE CAREGIVING ENVIRONMENT AND THE DEVELOPMENT OF PRESCHOOLERS	American Journal of Orthopsychiatry	0002-9432	10.1037/h0079693		1995-07	364-379		3	65
Outcome	thesis	2001	Hayes, Vicki Lynn	emotion regulation and a model of projected psychopathology resulting from attachment deficits and distortions.				http://libpro	2001-07		549		
Study Type	journalArticle	1988	Hayford, S. M.; Epps, R. P.; Dahl-Regis, M.	Behavior and development patterns in children born to heroin-addicted and methadone-addicted mothers	Journal of the National Medical Association	0027-9684			1988-11	1197-1200		11	80
Exposure	journalArticle	1997	Heffelfinger, A.; Craft, S.; Shyken, J.	Visual attention in children with prenatal cocaine exposure.	Journal of the International Neuropsychological Society : JINS	1355-6177 1355-6177			1997-05	237-245		3	3

© 2020 Nelson LF et al. *JAMA Network Open.*

Exposure	journalArticle	2002	Heffelfinger, Amy K.; Craft, Suzanne; White, Desiree A.; Shyken, Jaye	Visual attention in preschool children prenatally exposed to cocaine: implications for behavioral regulation.	Journal of the International Neuropsychological Society : JINS	1355-6177 1355-6177			2002-01	21-Dec		1	8
Exposure	journalArticle	2013	Hegarty, Mary; Calder, Alyson; Davies, Kylie; Shave, Margaret; Christiansen, Elaine; Meyer, Tessa; von Ungern-Sternberg, Britta S.	Does take-home analgesia improve postoperative pain after elective day case surgery? A comparison of hospital vs parent-supplied analgesia.	Paediatric anaesthesia	1460-9592 1	10.1111/pan.12077		2013-05	385-389		5	23
Outcome	thesis	2013	Heller, N. A.	Temperament and dyadic interactions in infants of opiate-dependent mothers				https://sear	2013		146		
Outcome	journalArticle	2017	Heller, N. A.; Logan, B. A.; Morrison, D. G.; Paul, J. A.; Brown, M. S.; Hayes, M. J.	Neonatal abstinence syndrome: Neurobehavior at 6 weeks of age in infants with or without pharmacological treatment for withdrawal.	Developmental Psychobiology	0012-1630	10.1002/dev	http://libpro	2017	574-582		5	59
Exposure	journalArticle	2005	Hender E.A.; Balit C.R.	Which medicines do young children access from blister packs? [1]	Medical Journal of Australia	0025-729X		http://www	2005	594		11	182
Study Type	journalArticle	1997	Hendrickse, R. G.	Of sick turkeys, kwashiorkor, malaria, perinatal mortality, heroin addicts and food poisoning: research on the influence of aflatoxins on child health in the tropics.	Annals of tropical medicine and parasitology	0003-4983 0003-4983			1997-10	787-793		7	91
Exposure	journalArticle	2009	Heras-Herraiz C.I.	Premature rupture of membranes of the second identical twin preterm births	Matronas Profesion	1578-0740		http://www	2009	25-27		3	10
Outcome	journalArticle	1995	Hickey, John E.; Suess, Patricia E.; Newlin, David B.; Spurgeon, Loretta; Porges, Stephen W.	Vagal tone regulation during sustained attention in boys exposed to opiates in utero	Addictive Behaviors	0306-4603	10.1016/030	http://www	1/1/95	43-59		1	20
Exposure	thesis	2005	Hill, Patricia D.	Addiction and recovery experiences of African American women: A phenomenological study				http://libpro	2005		218		
Outcome	journalArticle	2012	Himes, Sarah K.; Goodwin, Robert S.; Rock, Colleen M.; Jones, Hendree E.; Johnson, Rolley E.; Wilkins, Diana G.; Huestis, Marilyn A.	Methadone and metabolites in hair of methadone-assisted pregnant women and their infants.	Therapeutic drug monitoring	1536-3694 0	10.1097/FTD.0b013e3182		2012-06	337-344		3	34
Exposure	journalArticle	2012	Hippard, Helena K.; Govindan, Kalyani; Friedman, Ellen M.; Sulek, Marcelle; Giannoni, Carla; Larrier, Deidre; Minard, Charles G.; Watcha, Mehernoor F.	intranasal fentanyl, intravenous morphine, and intramuscular morphine in pediatric patients undergoing bilateral myringotomy and placement of ventilating tubes.	Anesthesia and analgesia	1526-7598 0	10.1213/ANE.0b013e3182		2012-08	356-363		2	115
Age	thesis	2017	Hock, Alyson J.	Processing of spacial information in social and non-social stimuli by opioid-exposed and non-exposed newborns.				http://uknov	2017		73		
Outcome	thesis	2018	Hock, Alyson Jo	Processing of spatial information in social and non-social stimuli by opioid-exposed and non-exposed newborns				http://libpro	2018				
Exposure	thesis	2016	Hoffman, Kevin R.	Developing a faith-based early intervention program for adults with alcohol and drug issues				http://libpro	2016		349		
Study Type	journalArticle	1998	Hogan, D. M.	Annotation: The psychological development and welfare of children of opiate and cocaine users: Review and research needs	Journal of Child Psychology and Psychiatry and Allied Disciplines	0021-9630	10.1017/S0021963098002		1998-07	609-620		5	39
Exposure	journalArticle	2008	Holsti L.; Grunau R.E.; Oberlander T.F.; Osiovich H.	Is it painful or not? Discriminant validity of the Behavioral Indicators of Infant Pain (BIIP) scale	Clinical Journal of Pain	0749-8047	10.1097/AJP	http://www	2008	83-88		1	24
Exposure	journalArticle	1974	Holzman, P. S.	Personality.	Annual review of psychology	0066-4308 0	10.1146/annurev.ps.25.02		1974	247-276			25
Study Type	journalArticle	2016	Hon K.L.; Chan M.H.M.; Ng M.H.J.; Ho C.C.; Tsang K.Y.C.; Tam W.H.; Ho C.S.	Urine comprehensive drug screen, low birth weight and withdrawal symptoms in a neonatal unit: A case control study	Current Clinical Pharmacology	1574-8847	10.2174/157	http://www	2016	274-281		4	11
Outcome	thesis	2007	Honde, Christina K.	The relation of atypical maternal behavior (ìdisrupted communicationî) to maternal risk factors and infant attachment				http://libpro	2007		113		
Exposure	thesis	1999	Hoogbruin, Amandah Lea	Affirming life despite a poisoned fate: A grounded theory of reproductive decision-making among women living with HIV				http://libpro	1999		217		
Exposure	journalArticle	2015	Hoover, Rebecca M.; Hayes, V. Autumn Gombert; Erramouspe, John	Association Between Prenatal Acetaminophen Exposure and Future Risk of Attention Deficit/Hyperactivity Disorder in Children.	Annals of Pharmacotherapy	1060-0280	10.1177/106	http://libpro	2015-12	1357-1361		12	49
Outcome	thesis	2013	Hopsicker, Rachel J.	The Impact of Parenting Interventions on Family Functioning for Women in Residential Substance Abuse Treatment				http://libpro	2013		141		
Exposure	thesis	2012	Horn, Carrie A.	Evaluation of a substance abuse treatment program for forensically committed individuals with cognitive impairment and severe mental illness				http://libpro	2012		186		

© 2020 Nelson LF et al. *JAMA Network Open.*

Exposure	journalArticle	1977	Horowitz, Frances Degen; Ashton, Jennifer; Culp, Rex; Gaddis, Ed; Levin, Stanley; Reichmann, Brian	The effects of obstetrical medication on the behavior of Israeli newborn infants and some comparisons with Uruguayan and American infants.	Child Development	0009-3920	10.2307/112	http://libpro	1977-12	1607-1623		4	48
Exposure	thesis	2004	Horvath, Violet Elizabeth	Comparing substantiated child maltreatment reports for mothers with and without a service use record of substance abuse or dependence in low -income families				http://libpro	2004		232		
Exposure	journalArticle	1993	Houpt M.I.	Project USAP--Part III: Practice by heavy users of sedation in pediatric dentistry.	ASDC journal of dentistry for children			http://www	1993	183-185		3	60
Study Type	journalArticle	1982	Householder J.; Hatcher R.; Burns W.; Chasnoff I.	Infants born to narcotic-addicted mothers.	Psychological bulletin	0033-2909		http://www	1982	453-468		2	92
Exposure	journalArticle	1986	Howard J.; Kropenske V.; Tyler R.	The long-term effects on neurodevelopment in infants exposed prenatally to PCP	NIDA Research Monograph Series	1046-9516		http://www	1986	237-251		(Howard J.; I	NO. 64
Exposure	thesis	2006	Howard, David L.	Incarceration during pregnancy and birth outcomes				http://libpro	2006		159		
Study Type	bookSection	2004	Howell, Karen K.; Coles, Claire D.; Kable, Julie	The Medical and Developmental Consequences of Prenatal Drug Exposure.	Handbook of the medical consequences of alcohol and drug abuse.			http://libpro	2004	281-302			
Exposure	journalArticle	2015	Huang, Annie; Tanbonliong, Thomas	Oral Sedation Postdischarge Adverse Events in Pediatric Dental Patients.	Anesthesia progress	0003-3006 0	10.2344/0003-3006-62.3.		2015	91-99		3	62
Outcome	journalArticle	1997	Hulse G.K.; Milne E.; English D.R.; Holman C.D.J.	The relationship between maternal use of heroin and methadone and infant birth weight	Addiction	0965-2140		http://www	1997	1571-1579		11	92
No Control Group	journalArticle	2016	Humbarger, Olivia; Galanto, Daniel; Saia, Kelley; Bagley, Sarah M.; Wachman, Elisha M.; Brogly, Susan B.	Childhood Health and Development in a Cohort of Infants Exposed Prenatally to Methadone or Buprenorphine	Journal of Addiction Research & Therapy	2155-6105	10.4172/215	https://www	1/29/16				
Included	journalArticle	2008	Hunt, R. W.; Tzioumi, D.; Collins, E.; Jeffery, H. E.	Adverse neurodevelopmental outcome of infants exposed to opiate in-utero	Early Human Development	3783782	10.1016/j.ea	http://linking	2008	29-35		1	84
Data Format	journalArticle	1990	Huntington, Lee; Hans, Sydney L.; Zeskind, Philip S.	The relations among cry characteristics, demographic variables, and developmental test scores in infants prenatally exposed to methadone.	Infant Behavior & Development	1879-0453	10.1016/016	http://libpro	1990-10	533-538		4	13
Exposure	journalArticle	1997	Hurt, H.; Malmud, E.; Betancourt, L.; Brodsky, N. L.; Giannetta, J.	A prospective evaluation of early language development in children with in utero cocaine exposure and in control subjects.	The Journal of pediatrics	0022-3476 0022-3476			1997-02	310-312		2	130
Study Type	journalArticle	1982	Hutchings, D. E.	Methadone and heroin during pregnancy: a review of behavioral effects in human and animal offspring.	Neurobehavioral toxicology and teratology	0275-1380 0275-1380			1982-08	429-434		4	4
Study Type	journalArticle	1993	Hutchings, D. E.; Zmitrovich, A.; Church, S.; Malowany, D.	Methadone during pregnancy: the search for a valid animal model.	Annali dell'Istituto superiore di sanita	0021-2571 0021-2571			1993	439-444		3	29
Age	journalArticle	2016	Ibach B.W.; Johnson P.N.; Ernst K.D.; Harrison D.; Miller J.L.	Initial Dosing and Taper Complexity of Methadone and Morphine for Treatment of Neonatal Abstinence Syndrome	Journal of Pharmacy Technology	1549-4810	10.1177/875	http://www	2016	216-222		5	32
Outcome	thesis	2011	Illangasekare, Samantha Lakmali	The role of the intimate partner violence, HIV, and substance abuse syndemic in women's mental health				http://libpro	2011		183		
Outcome	journalArticle	2011	Inturi R.; Ramamurthy R.; McLerran C.; Matula K.; Daniels W.; Nair P.; Michalek J.	substance abuse does not compound the effect of gestational age on outcome of infants less than 1500 grams	Journal of Investigative Medicine	1081-5589		http://www	2011	453		2	59
Outcome	journalArticle	1985	Iosub, S.; Fuchs, M.; Bingol, N.; Stone, R. K.; Gromisch, D. S.; Wasserman, E.	Incidence of major congenital malformations in offspring of alcoholics and polydrug abusers.	Alcohol (Fayetteville, N.Y.)	0741-8329 0741-8329			1985-06	521-523		3	2
Study Type	journalArticle	1991	Jacobson J.L.; Jacobson S.W.	Assessment of teratogenic effects on cognitive and behavioral development in infancy and childhood	NIDA Research Monograph Series	1046-9516		http://www	1991	248-261		114	
Exposure	journalArticle	1994	Jacobson, J. L.; Jacobson, S. W.; Sokol, R. J.	Effects of prenatal exposure to alcohol, smoking, and illicit drugs on postpartum somatic growth.	Alcoholism, clinical and experimental research	0145-6008 0145-6008			1994-04	317-323		2	18
Exposure	journalArticle	1996	Jacobson, S. W.; Jacobson, J. L.; Sokol, R. J.; Martier, S. S.; Chiodo, L. M.	New evidence for neurobehavioral effects of in utero cocaine exposure.	The Journal of pediatrics	0022-3476 0022-3476			1996-10	581-590		4	129
Exposure	journalArticle	2011	Jakobovi? J.; Butkovi? D.	The pharmacokinetic and pharmacodynamic characteristics of analgesics in children	Paediatria Croatica	1330-1403		http://www	2011	35-41		1	55

© 2020 Nelson LF et al. *JAMA Network Open.*

Outcome	thesis	2016	James, Deborah L	Treatment experiences of adult substance dependent females: A qualitative study				http://libpro	2016		165		
Outcome	journalArticle	2003	Jansson L.M.; Svikis D.S.; Beilenson P.	Effectiveness of Child Case Management Services for Offspring of Drug-Dependent Women	Substance Use and Misuse	1082-6084	10.1081/JA-	http://www	2003	1933-1952		14	38
Age	journalArticle	2009	Jansson L.M.; Velez M.; Harrow C.	The opioid-exposed newborn: Assessment and pharmacologic management	Journal of Opioid Management	1551-7489		http://www	2009	47-55		1	5
Age	journalArticle	2012	Jansson, Lauren M; Velez, Martha	Neonatal abstinence syndrome.	Current Opinion in Pediatrics	1040-8703		http://libpro	2012-04	252-258		2	24
Age	journalArticle	2010	Jansson, Lauren M.; DiPietro, Janet A.; Elko, Andrea; Velez, Martha	Infant autonomic functioning and neonatal abstinence syndrome.	Drug and Alcohol Dependence	0376-8716	10.1016/j.dr	http://libpro	6/1/10	198-204		3-Jan	109
Outcome	journalArticle	2017	Krystle; Spencer, Nancy; Tuten, Michelle; Jones, Hendree; Rios, Rebeca; King, Van L.; Gandotra, Neeraj; Millio, Lorraine; DiPietro, Janet A.	Maternal buprenorphine treatment and infant outcome.	Drug and Alcohol Dependence	0376-8716	10.1016/j.dr	http://libpro	11/1/17	56-61			180
No Control Group	thesis	2014	Jeong, Jessica	COGNITIVE DEVELOPMENT OF SUBSTANCE-EXPOSED CHILDREN INVOLVED WITH AN EARLY INTERVENTION PROGRAM					2014-03		69		
Outcome	journalArticle	1984	Jeremy R.J.; Bernstein V.J.	Dyads at risk: methadone-maintained women and their four-month-old infants.	Child development	0009-3920		http://www	1984	1141-1154		4	55
			Jernite 1999										
Data Format	thesis	2018	Jimenez, Pamela W.	Treatment impact and neurobehavioral effects of neonates with intrauterine exposure to buprenorphine versus methadone				http://libpro	2018				
Outcome	journalArticle	2018	Johnson E.E.; Schulte K.; Seidel J.; Shakib J.; Buchi K.; Fung C.	The effect of in utero polysubstance exposure on length of stay and length of pharmacologic treatment compared with opioids alone	Journal of Investigative Medicine	1708-8267	10.1136/jim-	http://www	2018	87-88		1	66
Outcome	journalArticle	2003	Johnson K.; Gerada C.; Greenough A.	Substance misuse during pregnancy	British Journal of Psychiatry	0007-1250	10.1192/bjp.	http://www	2003	187-189		SEPT.	183
Age	journalArticle	2003	Johnson K.; Gerada C.; Greenough A.	Treatment of neonatal abstinence syndrome	Archives of Disease in Childhood: Fetal and Neonatal Edition	0003-9888		http://www	2003	F2-F5		1	88
Outcome	journalArticle	1990	Johnson, H. L.; Glassman, M. B.; Fiks, K. B.; Rosen, T. S.	Resilient children: individual differences in developmental outcome of children born to drug abusers.	The Journal of genetic psychology	0022-1325 0	10.1080/00221325.1990.9		1990-12	523-539		4	151
Included	journalArticle	1982	Johnson, H. L.; Rosen, T. S.	Prenatal methadone exposure: effects on behavior in early infancy.	Pediatric pharmacology (New York, N.Y.)	0270-322X 0270-322X			1982	113-120		2	2
Included	journalArticle	1985	Johnson, Helen L.; Diano, Angela; Rosen, Tove S.	24-month neurobehavioral follow-up of children of methadone-maintained mothers.	Annual Progress in Child Psychiatry & Child Development	0066-4030		http://libpro	1985	20-Nov			
Exposure	journalArticle	1997	Johnson, Jeanne M.; Seikel, J. Anthony; Madison, Charles L.; Foose, Shari M.; Rinard, Keri D.	Standardized test performance of children with a history of prenatal exposure to multiple drugs/cocaine.	Journal of Communication Disorders	0021-9924	10.1016/S00	http://libpro	1997-01	45-73		1	30
Outcome	thesis	2014	Johnson, Kimberly A.	Mapping the Addiction Treatment Gap				http://libpro	2014		167		
Study Type	journalArticle	2012	Jones H.E.; Heil S.H.; Baewert A.; Arria A.M.; Kaltenbach K.; Martin P.R.; Coyle M.G.; Selby P.; Stine S.M.; Fischer G.	Buprenorphine treatment of opioid-dependent pregnant women: a comprehensive review.	Addiction (Abingdon, England)	1360-0443		http://www	2012	27-May		(Jones H.E.)	107 Suppl 1
Age	journalArticle	2010	Jones H.E.; O'Grady K.E.; Johnson R.E.; Velez M.; Jansson L.M.	Infant neurobehavior following prenatal exposure to methadone or buprenorphine: results from the neonatal intensive care unit network neurobehavioral scale.	Substance use & misuse	1532-2491		http://www	2010	2244-2257		13	45
Study Type	journalArticle	2015	Jones, HendrÈe E.; Fielder, Andrea	Neonatal abstinence syndrome: Historical perspective, current focus, future directions.	Preventive Medicine	0091-7435	10.1016/j.yp	http://libpro	2015-11	17-Dec			80
Study Type	journalArticle	2000	Jonquil S.G.	Paradigm shift.	Midwifery today with international midwife	1551-8892		http://www	2000	35-36		54	
Study Type	journalArticle	2000	Joseph, H.; Stancliff, S.; Langrod, J.	Methadone maintenance treatment (MMT): a review of historical and clinical issues.	The Mount Sinai journal of medicine, New York	0027-2507 0027-2507			2000-11	347-364		6-May	67

© 2020 Nelson LF et al. *JAMA Network Open.*

Exposure	journalArticle	2012	Joya X.; Gomez-Culebras M.; Callejon A.; Friguls B.; Puig C.; Ortigosa S.; Morini L.; Garcia-Algar O.; Vall O.	Cocaine use during pregnancy assessed by hair analysis in a Canary Islands cohort	BMC Pregnancy and Childbirth	1471-2393	10.1186/147	http://www	2012	2		(Joya X.; Gomez-Culebras	
Outcome	journalArticle	2016	Joya X.; Marchei E.; Salat-Batlle J.; GarcÌa-Algar O.; Calvaresi V.; Pacifici R.; Pichini S.	Drugs of abuse in maternal hair and paired neonatal meconium: an objective assessment of foetal exposure to gestational consumption	Drug Testing and Analysis	1942-7611	10.1002/dta.	http://www	2016	864-868		8	8
Exposure	journalArticle	2009	Joya, Xavier; Papaseit, Esther; Civit, Ester; Pellegrini, Manuela; Vall, Oriol; Garcia-Algar, Oscar; Scaravelli, Giulia; Pichini, Simona	Unsuspected exposure to cocaine in preschool children from a Mediterranean city detected by hair analysis.	Therapeutic drug monitoring	1536-3694 0	10.1097/FTD.0b013e3181		2009-06	391-395		3	31
Outcome	thesis	2012	Kaboski, Juhi Ryo	An empirically-derived typology of risks for mothers with substance use problems				http://libpro	2012		155		
Outcome	journalArticle	2007	Kahila H.; Kivitie-Kallio S.; Halmesm‰ki E.; Valanne L.; Autti T.	Brain magnetic resonance imaging of infants exposed prenatally to buprenorphine.	Acta radiologica (Stockholm, Sweden : 1987)	0284-1851	10.1080/028	http://www	2007	228-231		2	48
Outcome	journalArticle	2008	Kahila, Hanna; Stefanovic, Vedran; Loukovaara, Mikko; Alfthan, Henrik; Hamalainen, Esa; Halmesmaki, Erja	Prenatal buprenorphine exposure: effects on biochemical markers of hypoxia and early neonatal outcome.	Acta obstetricia et gynecologica Scandinavica	1600-0412 0	10.1080/00016340802460		2008	1213-1219		11	87
Outcome	thesis	2011	Kahila, Kahila	Substance misuse problems during pregnancy with special emphasis on buprenorphine				https://helda	2011		107		
Outcome	journalArticle	2007	Kahlert, Christian; Rudin, Christoph; Kind, Christian	Sudden infant death syndrome in infants born to HIV-infected and opiate-using mothers.	Archives of disease in childhood	1468-2044 0	10.1136/adc.2007.117192		2007-11	1005-1008		11	92
Exposure	journalArticle	2004	Kain Z.N.; Caldwell-Andrews A.A.; Krivutza D.M.; Weinberg M.E.; Wang S.-M.; Gaal D.	Induction of Anesthesia and the Use of Preoperative Sedative Premedication in the United States, 1995-2002: Results of a Follow-Up National Survey	Anesthesia and Analgesia	0003-2999		http://www	2004	1252-1259		5	98
Outcome	journalArticle	2013	Kallen, Bengt; Finnstrom, Orvar; Nygren, Karl-Gosta; Otterblad Olausson, Petra	Maternal drug use during pregnancy and asthma risk among children.	Pediatric allergy and immunology : official publication of the European Society of Pediatric Allergy and Immunology	1399-3038 0	10.1111/pai.12034		2013-02	28-32		1	24
Study Type	journalArticle	2016	Källén, Bengt; Reis, Margareta	Ongoing Pharmacological Management of Chronic Pain in Pregnancy.	Drugs	0012-6667	10.1007/s40	http://libpro	2016-06	915-924		9	76
Data Format	journalArticle	1987	Kaltenbach K.; Finnegan L.P.	Perinatal and developmental outcome of infants exposed to methadone in-utero.	Neurotoxicology and teratology	0892-0362		http://www	1987	311-313		4	9
Age	journalArticle	2012	Kaltenbach K.; Holbrook A.M.; Coyle M.G.; Heil S.H.; Salisbury A.L.; Stine S.M.; Martin P.R.; Jones H.E.	Predicting treatment for neonatal abstinence syndrome in infants born to women maintained on opioid agonist medication.	Addiction (Abingdon, England)	1360-0443		http://www	2012	45-52		(Kaltenbach	107 Suppl 1
Study Type	journalArticle	1996	Kaltenbach K.A.	Exposure to opiates: behavioral outcomes in preschool and school-age children.	NIDA research monograph	1046-9516		http://www	1996	230-241		(Kaltenbach	164
Study Type	journalArticle	1984	Kaltenbach, K.; Finnegan, L. P.	Developmental outcome of children born to methadone maintained women: a review of longitudinal studies	Neurobehavioral Toxicology and Teratology	0275-1380			1984-08	271-275		4	6
Study Type	journalArticle	1994	Kaltenbach, Karol A.	Effects of in-utero opiate exposure: New paradigms for old questions.	Drug and Alcohol Dependence	0376-8716	10.1016/037	http://libpro	1994-10	83-87		2	36
Study Type	bookSection	1992	Kaltenbach, Karol; Finnegan, Loretta P.	Methadone maintenance during pregnancy: Implications for perinatal and developmental outcome.	Perinatal substance abuse: Research findings and clinical implications.			http://libpro	1992	239-253			
Included	journalArticle	1989	Kaltenbach, Karol; Finnegan, Loretta P.	Children exposed to methadone in utero: Assessment of developmental and cognitive ability.	Annals of the New York Academy of Sciences	0077-8923	10.1111/j.17	http://libpro	1989-06	360-362			562
No Control Group	journalArticle	1986	Kaltenbach, Karol; Finnegan, Loretta P.	Neonatal abstinence syndrome, pharmacotherapy and developmental outcome.	Neurobehavioral Toxicology & Teratology			http://libpro	1986-07	353-355		4	8
Age	journalArticle	2016	Kaltenbach, Karol; Jones, Hendree E.	Neonatal Abstinence Syndrome: Presentation and Treatment Considerations.	Journal of Addiction Medicine	1932-0620	10.1097/AD	http://libpro	8/7/16	217-223		4	10
No Control Group	journalArticle	2018	Kaltenbach, Karol; O'Grady, Kevin E.; Heil, Sarah H.; Salisbury, Amy L.; Coyle, Mara G.; Fischer, Gabriele; Martin, Peter R.; Stine, Susan; Jones, Hendree E.	Prenatal exposure to methadone or buprenorphine: Early childhood developmental outcomes.	Drug and alcohol dependence	1879-0046 0	10.1016/j.drugalcdep.201		4/1/18	40-49			185
Outcome	thesis	2015	Kamdar, Sarah	The lingering effects of addiction: Measuring anxiety, loneliness, and self-esteem in siblings of addicts				http://libpro	2015		155		
Outcome	journalArticle	1993	Kandall, S. R.; Gaines, J.; Habel, L.; Davidson, G.; Jessop, D.	Relationship of maternal substance abuse to subsequent sudden infant death syndrome in offspring.	The Journal of pediatrics	0022-3476	0022-3476		1993-07	120-126		1	123

© 2020 Nelson LF et al. *JAMA Network Open.*

Outcome	thesis	1999	Kane, Anne T.	Finding a way to get help and paying the price: Women and infants in Medicaid managed care				http://libpro	1999		233		
Exposure	journalArticle	1989	Kangas-Saarela T.; Koivisto M.; Jouppila R.; Jouppila P.; Hollmen A.	Comparison of the effects of general and epidural anaesthesia for caesarean section on the neurobehavioural responses of newborn infants	Acta Anaesthesiologica Scandinavica	0001-5172		http://www	1989	313-319		4	33
Exposure	thesis	2014	Kapeleris, Andrea R.	Children's Socio-Emotional Development and Adjustment: Role of Maternal Trauma, Mentalization and Parenting Style				http://libpro	2014		195		
Study Type	thesis	2018	Kaplan, Rachel S. W.	The Rhetoric of the Opioid Crisis and Addiction to Prescription Pain Medicine				http://libpro	2018		174		
Outcome	journalArticle	1992	Karasik J.B.; Marion R.W.; Martinez-Frias M.-L.; Rodriguez-Pinilla E.	Gastrointestinal and renal malformations associated with prenatal exposure to alcohol and other teratogens [2]	American Journal of Medical Genetics	0148-7299		http://www	1992	853-854		6	42
Exposure	journalArticle	1991	Kavanagh, C. K.; Lasoff, E.; Eide, Y.; Freeman, R.; McEttrick, M.; Dar, R.; Helgerson, R.; Remensynder, J.; Kalin, N.	Learned helplessness and the pediatric burn patient: dressing change behavior and serum cortisol and beta-endorphin.	Advances in pediatrics	0065-3101 0065-3101			1991	335-363			38
Study Type	journalArticle	2001	Keen, J.; Alison, L. H.	Drug misusing parents: key points for health professionals.	Archives of disease in childhood	1468-2044 0003-9888			2001-10	296-299		4	85
Exposure	journalArticle	2004	Keidan, Ilan; Zaslansky, Ruth; Eviatar, Ephraim; Segal, Samuel; Sarfaty, Shlomo M.	Intraoperative ketorolac is an effective substitute for fentanyl in children undergoing outpatient adenotonsillectomy.	Paediatric anaesthesia	1155-5645 1	10.1046/j.1460-9592.2003		2004-04	318-323		4	14
Study Type	journalArticle	2014	Kelly L.; Guilfoyle J.; Dooley J.; Antone I.; Gerber-Finn L.; Dooley R.; Brunton N.; Kakegamuck K.; Muileboom J.; Hopman W.; Cromarty H.; Linkewich B.; Maki J.	Incidence of narcotic abuse during pregnancy in northwestern Ontario: Three-year prospective cohort study	Canadian Family Physician	0008-350X		http://www	2014	e493-e498		10	60
Outcome	journalArticle	2011	Kelly L.; Minty B.; Madden S.; Dooley J.; Antone I.	The occasional management of narcotic exposure in neonates.	journal of the Society of Rural Physicians of Canada = Journal canadien de la mÈdecine rurale : le journal officiel de la SociÈtÈ de	1488-237X		http://www	2011	98-101		3	16
Outcome	journalArticle	2012	Kelly L.E.; Riede M.J.; Bridgman-Acker K.; Lauwers A.; Madadi P.; Koren G.	Are infants exposed to methadone in utero at an increased risk for mortality?	Journal of Population Therapeutics and Clinical Pharmacology	1710-6222		http://www	2012	e160-e165		2	19
Outcome	thesis	2002	Kelly, Kimberly Patrice	Ongoing substance abuse and its effect on the attachment quality of toddlers				http://libpro	2002		188		
Outcome	journalArticle	2017	Kelty, Erin; Hulse, Gary	A retrospective cohort study of the health of children prenatally exposed to methadone, buprenorphine or naltrexone compared with non-exposed control children.	The American journal on addictions	1521-0391 1	10.1111/ajad.12642		2017-12	845-851		8	26
Study Type	journalArticle	2008	Kennedy MS	In the news. From the National Institute of Nursing Research: long-term effects of in utero drug exposure.	AJN American Journal of Nursing	0002-936X		http://libpro	2008-05	21-21		5	108
Outcome	thesis	2015	Kepple, Nancy Jo	Children? An examination of the relationships between substance use patterns, social support type, and child maltreatment behaviors				http://libpro	2015		155		
Outcome	journalArticle	2005	Kerwin M.E.	Collaboration between child welfare and substance-abuse fields: Combined treatment programs for mothers	Journal of Pediatric Psychology	0146-8693	10.1093/jpe	http://www	2005	581-597		7	30
Exposure	journalArticle	2011	Kiblawi Z.N.; Smith L.M.; Lagasse L.L.; Derauf C.; Newman E.; Shah R.; Arria A.; Huestis M.; Haning W.; Strauss A.; Della G.S.; Dansereau L.M.; Lester B.M.	Prenatal methamphetamine use and neonatal and infant neurobehavioral outcome: Results from the infant development, environment, and lifestyle (Ideal) study	Journal of Investigative Medicine	1081-5589		http://www	2011	172		1	59
Exposure	journalArticle	2014	LaGasse, Linda L.; Derauf, Chris; Newman, Elana; Shah, Rizwan; Arria, Amelia; Huestis, Marilyn; Haning, William; Strauss, Arthur; DellaGrotta, Sheri;	Prenatal methamphetamine exposure and neonatal and infant neurobehavioral outcome: Results from the ideal study.	Substance Abuse	0889-7077	10.1080/088	http://libpro	2014-01	68-73		1	35
Exposure	journalArticle	2017	Kim, J.?S.; Kim, G. W.; Park, D. H.; Ahn, H. E.; Chang, M. Y.; Kim, J. Y.	Effects of scalp nerve block on pain and emergence agitation after paediatric nevus surgery: a clinical trial.	Acta Anaesthesiologica Scandinavica	0001-5172	10.1111/aas	http://libpro	2017-09	935-941		8	61
Exposure	thesis	2018	Kim, Jiyoung	Detection of in utero Cannabis Exposure by Umbilical Cord Analysis				http://libpro	2018		31		
Outcome	journalArticle	1991	KINNEY, HC; WHITE, WF	OPIOID RECEPTORS LOCALIZE TO THE EXTERNAL GRANULAR-CELL LAYER OF THE DEVELOPING HUMAN CEREBELLUM	Neuroscience	0306-4522	10.1016/0306-4522(91)90		1991	13-21		1	45
Outcome	journalArticle	1993	Kintz P.; Mangin P.	Evidence of gestational heroin or nicotine exposure by analysis of fetal hair	Forensic Science International	0379-0738	10.1016/037	http://www	1993	99-104		3-Jan	63
No Control Group	thesis	2015	Kivistˆ, Kaisa	HEALTH OF PRENATALLY BUPRENORPHINE-EXPOSED CHILDREN TO THREE YEARS OF AGE				http://ethes	2015		111		

© 2020 Nelson LF et al. *JAMA Network Open.*

Outcome	journalArticle	2014	Kivisto, K.; Alapulli, H.; Tupola, S.; Alaluusua, S.; Kivitie-Kallio, S.	Dental health of young children prenatally exposed to buprenorphine. A concern of child neglect?	European archives of paediatric dentistry : official journal of the European Academy of Paediatric Dentistry	1996-9805 1	10.1007/s40368-013-0095		2014-06	197-202		3	15
Outcome	journalArticle	2015	Kivisto, Kaisa; Tupola, Sarimari; Kivitie-Kallio, Satu	Prenatally buprenorphine-exposed children: health to 3 years of age.	European journal of pediatrics	1432-1076 0	10.1007/s00431-015-2562		2015-11	1525-1533		11	174
Exposure	journalArticle	2008	Knowlton A; Buchanan A; Wissow L; Pilowsky DJ; Latkin C; Knowlton, Amy; Buchanan, Amy; Wissow, Lawrence; Pilowsky, Daniel J; Latkin, Carl	Externalizing behaviors among children of HIV seropositive former and current drug users: parent support network factors as social ecological risks.	Journal of Urban Health	1099-3460		http://libpro	1/19/08	62-76		1	85
Study Type	journalArticle	2017	Ko, Jean Y.	CDC Grand Rounds: Public Health Strategies to Prevent Neonatal Abstinence Syndrome	MMWR. Morbidity and Mortality Weekly Report	0149-219515	10.15585/m	https://www	2017				66
Exposure	journalArticle	1999	Kokinsky E.; Thornberg E.; ÷stlund A.L.; Larsson L.E.	Postoperative comfort in paediatric outpatient surgery	Paediatric Anaesthesia	1155-5645	10.1046/j.14	http://www	1999	243-251		3	9
Outcome	journalArticle	1994	Kolar, A. F.; Brown, B. S.; Haertzen, C. A.; Michaelson, B. S.	Children of substance abusers: the life experiences of children of opiate addicts in methadone maintenance.	The American journal of drug and alcohol abuse	0095-2990 0095-2990			1994	159-171		2	20
Exposure	journalArticle	1995	Kolmen, Barbara K.; Feldman, Heidi M.; Handen, Benjamin L.; Janosky, Janine E.	Naltrexone in young autistic children: A double-blind, placebo-controlled crossover study.	Journal of the American Academy of Child & Adolescent Psychiatry	0890-8567	10.1097/000	http://libpro	1995-02	223-231		2	34
Exposure	journalArticle	2012	Konefa? H.; Jaskot B.; Czeszy?ska M.B.	[Pethidine for labor analgesia; monitoring of newborn heart rate, blood pressure and oxygen saturation during the first 24 hours after the delivery].	Ginekologia polska	0017-0011		http://www	2012	357-362		5	83
Outcome	journalArticle	2015	Konijnenberg, C.; Melinder, A.	Visual selective attention is impaired in children prenatally exposed to opioid agonist medication.	European addiction research	1421-9891 1	10.1159/000366018		2015	63-70		2	21
Outcome	journalArticle	2013	Konijnenberg, C.; Melinder, A.	Neurodevelopmental investigation of the mirror neurone system in children of women receiving opioid maintenance therapy during pregnancy.	Addiction (Abingdon, England)	1360-0443 0	10.1111/j.1360-0443.2012		2013	154-160		1	108
Outcome	journalArticle	2016	Konijnenberg, C.; Sarfi, M.; Melinder, A.	Mother-child interaction and cognitive development in children prenatally exposed to methadone or buprenorphine	Early Human Development	1872-6232	10.1016/j.ea	http://www	2016	91-97		(Konijnenbe	101
Outcome	journalArticle	2018	Konijnenberg, Carolien; Jondalen, Nils Martin; Husby, Mikael Falkhaugen; Melinder, Annika	ERP correlates of cognitive control in children prenatally exposed to methadone or buprenorphine	Developmental Neuropsychology	8756-5641	10.1080/87565641.2018.1		2018	642-655		7	43
Outcome	journalArticle	2015	Konijnenberg, Carolien; Melinder, Annika	Executive function in preschool children prenatally exposed to methadone or buprenorphine	Child Neuropsychology	0929-7049, 1	10.1080/092	http://www	9/3/15	570-585		5	21
Exposure	thesis	2004	Koppelman, Nancy F.	Defying dependence: Learning from women in recovery and service providers about family survival				http://libpro	2004		447		
Exposure	journalArticle	1970	Kornetsky C.	Psychoactive drugs in the immature organism.	Psychopharmacologia	0033-3158		http://www	1970	105-136		2	17
Exposure	journalArticle	1998	Kosofsky BE	Cocaine-induced alterations in neuro-development.	Seminars in Speech & Language	0734-0478		http://libpro	1998-05	109-202		2	19
Outcome	journalArticle	2017	Kraft W.K.; Adeniyi-Jones S.C.; Chervoneva I.; Greenspan J.S.; Abatemarco D.; Kaltenbach K.; Ehrlich M.E.	Buprenorphine for the treatment of the neonatal abstinence syndrome	New England Journal of Medicine	1533-4406	10.1056/NEJ	http://www	2017	2341-2348		24	376
Exposure	thesis	2017	Kramlich, Debra L.	Experiences and Perceptions of Rural Postpartum Women with Substance Use Disorders Inclusive of Opioids Regarding Their Care				http://libpro	2017		191		
Outcome	thesis	2013	Krause, Kylene	Relationship Predictors of Prenatal Maternal Representations of the Child and Parenting Experiences One Year after Birth				http://libpro	2013		194		
Outcome	journalArticle	1982	Kreek, Mary J.	Opioid disposition and effects during chronic exposure in the perinatal period in man.	Advances in Alcohol & Substance Abuse	0270-3106	10.1300/J25	http://libpro	1982	21-53		4-Mar	1
Age	journalArticle	2016	Kreitinger C.; Gutierrez H.; Hamidovic A.; Schmitt C.; Sarangarm P.; Rayburn W.F.; Leeman L.; Bakhireva L.N.	The effect of prenatal alcohol co-exposure on neonatal abstinence syndrome in infants born to mothers in opioid maintenance treatment	Journal of Maternal-Fetal and Neonatal Medicine	1476-4954	10.3109/147	http://www	2016	783-788		5	29
Exposure	journalArticle	1988	Kretz F.J.; Schweitzer D.; Niggemeyer R.; Heppe M.; Abu Dorrah T.; Benesch G.; Striebel H.W.	Psychological preparation, premedication and induction of anaesthesia in children	Anasthesiologie und Intensivmedizin	0170-5334		http://www	1988	7-Jan		1	29
Outcome	thesis	1995	Kruse, Diane Buck	"Well-growness" of the newborn and factors contributing to low birth weight				http://libpro	1995		142		

© 2020 Nelson LF et al. *JAMA Network Open.*

Study Type	journalArticle	1985	Kuhnert B.R.; Kuhnert P.M.	Placental transfer of drugs, alcohol, and components of cigarette smoke and their effects on the human fetus	NIDA Research Monograph Series	1046-9516		http://www	1985	98-109		(Kuhnert B.F	NO. 60
Outcome	thesis	2006	Kyskan, Christina	Evolution of the fragile fighter: A qualitative exploration of seven mothers in a substance abuse treatment program				http://libpro	2006		229		
Exposure	journalArticle	2005	Lˆnnqvist P.-A.; Morton N.S.	Postoperative analgesia in infants and children	British Journal of Anaesthesia	0007-0912	10.1093/bja/	http://www	2005	59-68		1	95
Outcome	journalArticle	2003	Tronick, E. Z.; Bauer, C. R.; Shankaran, S.; Bada, H. S.; Wright, L. L.; Smeriglio, V. L.; Finnegan, L. P.; Maza, P. L.; Liu, J.	Prenatal drug exposure and maternal and infant feeding behaviour.	Archives of disease in childhood. Fetal and neonatal edition	1359-2998 1359-2998			2003-09	F391-399		5	88
Outcome	journalArticle	1998	LaGasse, L. L.; Van Vorst, R. F.; Brunner, S. M.; Lester, B. M.	Effects of in utero exposure to cocaine and/or opiates on infants' reaching behavior.	Annals of the New York Academy of Sciences	0077-8923 0077-8923			6/21/98	405-407			846
Exposure	thesis	2001	Laird, Roy James	When mom goes back to school: Mothers who attend adult education classes and their school -aged children				http://libpro	2001		261		
Exposure	journalArticle	2015	Lam, Jessica; Baello, Stephanie; Iqbal, Majid; Kelly, Lauren E.; Shannon, Patrick T.; Chitayat, David; Matthews, Stephen G.; Koren, Gideon	The ontogeny of P-glycoprotein in the developing human blood-brain barrier: implication for opioid toxicity in neonates.	Pediatric research	1530-0447 0	10.1038/pr.2015.119		2015-10	417-421		4	78
Exposure	journalArticle	2014	D.; Hagemann, Tracy M.; Lawrence, Shelley M.; Williams, Patricia K.; Anderson, Michael P.; Miller, Jamie L.	Association of fentanyl with neurodevelopmental outcomes in very-low-birth-weight infants.	The Annals of pharmacotherapy	1542-6270 1	10.1177/10600280135140		2014-03	335-342		3	48
Outcome	thesis	2016	Land, Kathryn Lynn	Current systems of care for pregnant and parenting opiate-addicted women in Broome County of New York State				http://libpro	2016		111		
Age	journalArticle	1982	Larin, HÈlËne M.	Drug and obstetric medication effects on infant behavior as measured by the Brazelton Neonatal Behavioral Assessment Scale.	Physical & Occupational Therapy in Pediatrics	0194-2638	10.1300/J00	http://libpro	1982	75-84		1	2
Age	journalArticle	1989	Larue M.; Challier P.; Robinet F.; Fontaine J.L.; Bernheim C.	Use of paregoric for treatment of neonatal narcotic withdrawal	Journal de Pharmacie Clinique	0291-1981		http://www	1989	231-232		4	8
Outcome	thesis	2016	Latuskie, Kiri Anna	Discontinuation patterns of substances of abuse in pregnancy in a cohort of women seeking addiction and parenting support: A mixed methods study				http://libpro	2016		166		
Outcome	thesis	2009	Laughinghouse, Janzlean	Exploring the parenting beliefs of substance abusing women				http://libpro	2009		95		
Outcome	thesis	2004	Laughon, Kathryn	Women's experiences of lifetime violence, mental illness, substance use and risk of sexually transmitted infections in a predominantly African American sample				http://libpro	2004		177		
Outcome	thesis	2011	Lawrence, Lyn Haselden	Motherhood and Reproduction in the Lives of Women with Bipolar Disorder				http://libpro	2011		345		
Outcome	journalArticle	1980	Lawson, M. S.; Wilson, G. S.	Parenting among women addicted to narcotics.	Child welfare	0009-4021 0009-4021			1980-02	67-79		2	59
Outcome	thesis	2012	Lean, R. E.	The mental health outcomes of children born to methadone dependent mothers: the role of out-of-home care at age 4.5-years				http://ir.can	2012				
Outcome	journalArticle	2013	Lean, Rachel E.; Pritchard, Verena E.; Woodward, Lianne J.	Child protection and out-of-home placement experiences of preschool children born to mothers enrolled in methadone maintenance treatment during pregnancy	Children and Youth Services Review	0190-7409	10.1016/j.childyouth.2013		2013-11	1878-1885		11	35
Exposure	journalArticle	2001	Leckman, E. B.; Mayes, L. C.; Hodgins, H. S.	Perceptions and attitudes toward prenatal cocaine exposure in young children.	Child psychiatry and human development	0009-398X 0009-398X			2001	313-328		4	31
Outcome	thesis	2012	Lee, S. J	The school readiness of children born to mothers maintained on methadone during pregnancy.				http://ir.can	2012				
Exposure	journalArticle	2017	Lee, Vincent R.; Connolly, Michael; Calello, Diane P.	Pediatric Poisoning by Ingestion: Developmental Overview and Synopsis of National Trends.	Pediatric Annals	0090-4481	10.3928/193	http://libpro	2017-12	443-448		12	46
Exposure	journalArticle	1992	Legido A.; Clancy R.R.; Spitzer A.R.; Finnegan L.P.	Electroencephalographic and behavioral-state studies in infants of cocaine-addicted mothers	American Journal of Diseases of Children	0002-922X		http://www	1992	748-752		6	146
Exposure	journalArticle	2015	Lenahan M.; Wells M.; Scarbecz M.	A Retrospective Study of 248 Pediatric Oral Sedations Utilizing the Combination of Meperidine and Hydroxyzine for Dental Treatment	The Journal of clinical pediatric dentistry	1053-4628	10.17796/10	http://www	2015	481-487		5	39

© 2020 Nelson LF et al. *JAMA Network Open.*

Age	journalArticle	1982	Lesser-Katz, Miriam	Some effects of maternal drug addiction on the neonate.	International Journal of the Addictions	0020-773X	10.3109/108	http://libpro	1982-07	887-896		5	17
Study Type	journalArticle	1999	Lester B.M.	Clinics in perinatology: Preface	Clinics in Perinatology	0095-5108		http://www	1999	xiii-xiv		1	26
Exposure	journalArticle	1982	Lester B.M.; Als H.; Brazelton T.B.	Regional obstetric anesthesia and newborn behavior: a reanalysis toward synergistic effects.	Child development	0009-3920		http://www	1982	687-692		3	53
Exposure	journalArticle	2001	Lester B.M.; ElSohly M.; Wright L.L.; Smeriglio V.L.; Verter J.; Bauer C.R.; Shankaran S.; Bada H.S.; Walls H.C.; Huesris M.A.; Finnegan L.P.; Maza P.L.	The maternal lifestyle study: Drug use by meconium toxicology and maternal self-report	Pediatrics	0031-4005	10.1542/ped	http://www	2001	309-317		2	107
Exposure	journalArticle	2002	Lester, B. M.; Tronick, E. Z.; LaGasse, L.; Seifer, R.; Bauer, C. R.; Shankaran, S.; Bada, H. S.; Wright, L. L.; Smeriglio, V. L.; Lu, J.; Finnegan, L. P.; Maza, P. L.	The maternal lifestyle study: Effects of substance exposure during pregnancy on neurodevelopmental outcome in 1-month-old infants	Pediatrics	0031-4005	10.1542/peds.110.6.1182		2002-12	1182-1192		6	110
Exposure	journalArticle	2000	Lester, Barry M.	Prenatal cocaine exposure and child outcome: A model for the study of the infant at risk.	Israel Journal of Psychiatry and Related Sciences	0333-7308		http://libpro	2000	223-235		3	37
Exposure	journalArticle	2010	Lester, Barry M.; Lagasse, Linda L.	Children of addicted women.	Journal of addictive diseases	1545-0848 1	10.1080/10550881003684		2010-04	259-276		2	29
Exposure	journalArticle	2003	Tronick, Edward Z.; Bauer, Charles R.; Shankaran, Seetha; Bada, Henrietta S.; Wright, Linda L.; Smeriglio, Vincent L.; Liu, Jing; Finnegan, Loretta P.;	The Maternal Lifestyle Study (MLS): effects of prenatal cocaine and/or opiate exposure on auditory brain response at one month.	The Journal of pediatrics	0022-3476 0	10.1067/mpd.2003.112		2003-03	279-285		3	142
Exposure	thesis	2010	Levine Kornfield, Sara V.	Maternal -fetal attachment, temporal orientation and locus of control: Implications for prenatal care behaviors and HIV risk reduction during pregnancy				http://libpro	2010		103		
Included	journalArticle	2018	Levine T.A.; Woodward L.J.	Early inhibitory control and working memory abilities of children prenatally exposed to methadone	Early Human Development	1872-6232	10.1016/j.ea	http://www	2018	68-75		(Levine T.A.;	116
Exposure	journalArticle	2011	Li J.; Huang Z.-L.; Zhang X.-T.; Luo K.; Zhang Z.-Q.; Mao Y.; Zhuang X.-B.; Lian Q.-Q.; Cao H.	Sufentanil reduces emergence agitation in children receiving sevoflurane anesthesia for adenotonsillectomy compared with fentanyl	Chinese Medical Journal	0366-6999	10.3760/cma	http://www	2011	3682-3685		22	124
Outcome	journalArticle	1985	Lief, N. R.	The drug user as a parent.	The International journal of the addictions	0020-773X 0020-773X			1985-01	63-97		1	20
Outcome	journalArticle	1983	Lifschitz M.H.; Wilson G.S.; O'Brian Smith E.; Desmond M.M.	Fetal and postnatal growth of children born to narcotic-dependent women	Journal of Pediatrics	0022-3476		http://www	1983	686-691		5	102
Included	journalArticle	1985	Lifschitz, M. H.; Wilson, G. S.; Smith, E. O.; Desmond, M. M.	Factors affecting head growth and intellectual function in children of drug-addicts	Pediatrics	0031-4005			1985	269-274		2	75
Outcome	journalArticle	2001	Lifshitz M.; Gavrilov V.; Galil A.; Landau D.	A four year survey of neonatal narcotic withdrawal: Evaluation and treatment	Israel Medical Association Journal	1565-1088		http://www	2001	17-20		1	3
Age	journalArticle	2015	Perrine C.G.; Li R.; Hudak M.; Correia J.A.; Creanga A.A.; Sappenfield W.M.; Curran J.; Blackmore C.; Watkins S.M.; Anjohrin S.	Infant and maternal characteristics in neonatal abstinence syndrome--selected hospitals in Florida, 2010-2011	MMWR. Morbidity and mortality weekly report	1545-861X		http://www	2015	213-216		8	64
Outcome	journalArticle	1994	Linday L.A.	Developmental changes in renal tubular function	Journal of Adolescent Health	1054-139X	10.1016/S10	http://www	1994	648-653		8	15
Study Type	journalArticle	1993	Lindenberg CS; Keith AB	Opiate abuse in pregnancy.	Annual Review of Nursing Research	0739-6686		http://libpro	1993-01	249-279			11
Study Type	journalArticle	1999	Lindenskov, L.; Johnsen, K.; Larsen, K.; Lange, A. P.	[Pregnancy and drug abuse].	Ugeskrift for laeger	0041-5782 0041-5782			9/6/99	5005-5009		36	161
Outcome	journalArticle	1996	Linet, M. S.; Gridley, G.; Cnattingius, S.; Nicholson, H. S.; Martinsson, U.; Glimelius, B.; Adami, H. O.; Zack, M.	Maternal and perinatal risk factors for childhood brain tumors (Sweden).	Cancer causes & control : CCC	0957-5243 0957-5243			1996-07	437-448		4	7
Exposure	journalArticle	1996	Littlejohn, I. H.; Tarling, M. M.; Flynn, P. J.; Ordman, A. J.; Aiken, A.	Post-operative pain relief in children following extraction of carious deciduous teeth under general anaesthesia: a comparison of nalbuphine and diclofenac.	European journal of anaesthesiology	0265-0215 0265-0215			1996-07	359-363		4	13
Outcome	journalArticle	2010	Liu A.J.W.; Jones M.P.; Murray H.; Cook C.-M.; Nanan R.	Perinatal risk factors for the neonatal abstinence syndrome in infants born to women on methadone maintenance therapy	Obstetrical and Gynecological Survey	0029-7828	10.1097/OG	http://www	2010	603-604		10	65
Exposure	journalArticle	2007	Liu W.F.; Laudert S.; Perkins B.; MacMillan-York E.; Martin S.; Graven S.	The development of potentially better practices to support the neurodevelopment of infants in the NICU	Journal of Perinatology	0743-8346	10.1038/sj.jp	http://www	2007	S48-S74		SUPPL. 2	27

© 2020 Nelson LF et al. *JAMA Network Open.*

Exposure	journalArticle	1993	Lochary M.E.; Wilson S.; Griffen A.L.; Coury D.L.	Temperament as a predictor of behavior for conscious sedation in dentistry.	Pediatric dentistry	0164-1263		http://www	1993	348-352		5	15
Exposure	journalArticle	2016	Locke, Robin L.; Lagasse, Linda L.; Seifer, Ronald; Lester, Barry M.; Shankaran, Seetha; Bada, Henrietta S.; Bauer, Charles R.	Effects of prenatal substance exposure on infant temperament vary by context.	Development and psychopathology	1469-2198 0	10.1017/S0954579415000		2016-05	309-326		2	28
Study Type	journalArticle	2013	Logan B.A.; Brown M.S.; Hayes M.J.	Neonatal abstinence syndrome: Treatment and pediatric outcomes	Clinical Obstetrics and Gynecology	0009-9201	10.1097/GRF	http://www	2013	186-192		1	56
Poste- Data Published Else	journalArticle	2011	Logan B.A.; Heller N.A.; Paul J.A.; Morrison D.G.; Brown M.; Krishnan R.; Hayes M.J.	Longitudinal developmental outcomes in the first year in opiateexposed infants: Role of prenatal alcohol exposure	Alcoholism: Clinical and Experimental Research	0145-6008	10.1111/j.15	http://www	2011	40A		(Logan B.A.;	35
No Control Group	thesis	2013	Logan, Beth A.	Neurocognition and prenatal exposure to methadone: Comparison of the event related potential and the Bayley Scales of Infant Development in the first year				https://sear	2013		130		
Outcome	thesis	2015	Loree, Amy M.	Toward enhancing treatment for pregnant smokers: Laying the groundwork for the use of complementary and alternative medicine approaches				http://libpro	2015		246		
Outcome	thesis	2011	Loree, Amy M.	association between violence exposure and substance use among high-risk mothers receiving home visitation services				http://libpro	2011		95		
Study Type	journalArticle	2017	Loudin, Sean; Murray, Sara; Prunty, Leesa; Davies, Todd; Evans, Joseph; Werthammer, Joseph	An Atypical Withdrawal Syndrome in Neonates Prenatally Exposed to Gabapentin and Opioids.	The Journal of pediatrics	1097-6833 0	10.1016/j.jpeds.2016.11.0		2017-02	286-288			181
Outcome	journalArticle	2017	Lowe, J. R.; Qeadan, F.; Leeman, L.; Shrestha, S.; Stephen, J. M.; Bakhireva, L. N.	The effect of prenatal substance use and maternal contingent responsiveness on infant affect	Early Human Development	1872-6232	10.1016/j.ea	http://www	2017	51-59		(Lowe J.) De	115
Outcome	thesis	2017	Ludwick, Susan Danielle	Postpartum Depression: Implementing an Evidence-Based Social Support Network in North Carolina				http://libpro	2017		85		
Exposure	journalArticle	2019	Lupattelli, Angela; Chambers, Cristina D.; Bandoli, Gretchen; Handal, Marte; Skurtveit, Svetlana; Nordeng, Hedvig	Hypnotics During Pregnancy With Motor and Communication Skills and Attention-Deficit/Hyperactivity Disorder Symptoms in Preschoolers.	JAMA Network Open		10.1001/jam	http://libpro	4/5/19	e191435-e191435		4	2
Exposure	thesis	2000	Lussier, Eileen Fasoli	A study of substance abuse treatment for an insured population: Consideration of gender, substance of choice and dual diagnosis				http://libpro	2000		168		
Exposure	journalArticle	1998	Lustbader A.S.; Mayes L.C.; McGee B.A.; Jatlow P.; Roberts W.L.	Incidence of passive exposure to crack/cocaine and clinical findings in infants seen in an outpatient service.	Pediatrics	1098-4275		http://www	1998	e5		1	102
Exposure	journalArticle	1998	MacGregor, R.; Evans, D.; Sugden, D.; Gaussen, T.; Levene, M.	Outcome at 5-6 years of prematurely born children who received morphine as neonates.	Archives of disease in childhood. Fetal and neonatal edition	1359-2998 1359-2998			1998-07	F40-43		1	79
Exposure	journalArticle	2001	Macmillan, C.; Magder, L. S.; Brouwers, P.; Chase, C.; Hittelman, J.; Lasky, T.; Malee, K.; Mellins, C. A.; Velez-Borras, J.	Head growth and neurodevelopment of infants born to HIV-1-infected drug-using women.	Neurology	0028-3878 0028-3878			10/23/01	1402-1411		8	57
Study Type	journalArticle	2011	Mactier H	The management of heroin misuse in pregnancy: time for a rethink?	Archives of Disease in Childhood -- Fetal & Neonatal Edition	1359-2998		http://libpro	2011-06	F457-60		6	96
Study Type	journalArticle	2015	Mactier H.; McGlone L.	Reply to letter from Drs. Jones, Heil and O'Grady	Early Human Development	1872-6232	10.1016/j.ea	http://www	2015	245		3	91
Study Type	journalArticle	2013	Mactier, Helen	Neonatal and longer term management following substance misuse in pregnancy.	Early Human Development	0378-3782	10.1016/j.ea	http://libpro	2013-11	887-892		11	89
Outcome	journalArticle	2013	Maguire D.J.	Mothers on methadone: Care in the NICU	Neonatal Network	1539-2880	10.1891/073	http://www	2013	409-415		6	32
Study Type	journalArticle	2016	Maguire D.J.; Taylor S.; Armstrong K.; Shaffer-Hudkins E.; Germain A.M.; Brooks S.S.; Cline G.J.; Clark L.	Long-Term Outcomes of Infants with Neonatal Abstinence Syndrome	Neonatal network : NN	1539-2880	10.1891/073	http://www	2016	277-286		5	35
Age	journalArticle	2015	Maguire, Denise J.; Rowe, Meredeth A.; Spring, Heather; Elliott, Amanda F.	Patterns of Disruptive Feeding Behaviors in Infants With Neonatal Abstinence Syndrome.	Advances in neonatal care : official journal of the National Association of Neonatal Nurses	1536-0911 1	10.1097/ANC.0000000000		2015-12	429-439; quiz E1-2		6	15
Age	journalArticle	2016	Maguire, Denise; Groer, Maureen	Neonatal abstinence syndrome and the gastrointestinal tract.	Medical hypotheses	1532-2777 0	10.1016/j.mehy.2016.10.0		2016-12	15-Nov			97
Outcome	thesis	2002	Mancuso, Richard Frank	Effects of lifespan childhood and partner victimization on women's drug -related problems and drug-related lifestyle activities				http://libpro	2002		395		

© 2020 Nelson LF et al. *JAMA Network Open.*

Study Type	journalArticle	2012	Mann C.J.	Baby steps toward recovery.	Nursing for women's health	1751-486X		http://www	2012	260, 259		3	16
Outcome	journalArticle	2003	Manworren, Renee C. B.; Hynan, Linda S.	Clinical validation of FLACC: preverbal patient pain scale.	Pediatric nursing	0097-9805 0097-9805			2003-04	140-146		2	29
Outcome	thesis	2009	Marcelin, Gardith-Eileen	Adjustment and attachment in urban children of mothers with substance use, HIV, and mental health problems				http://libpro	2009		173		
Outcome	journalArticle	1982	Marcus, J.; Hans, S. L.	A methodological model to study the effects of toxins on child development.	Neurobehavioral toxicology and teratology	0275-1380 0275-1380			1982-08	483-487		4	4
Outcome	journalArticle	1984	Marcus, J.; Hans, S. L.; Jeremy, R. J.	A longitudinal study of offspring born to methadone-maintained women. III. Effects of multiple risk factors on development at 4, 8, and 12 months.	The American journal of drug and alcohol abuse	0095-2990 0095-2990			1984	195-207		2	10
Outcome	journalArticle	1982	Marcus, J.; Hans, S. L.; Jeremy, R. J.	Differential motor and state functioning in newborns of women on methadone.	Neurobehavioral toxicology and teratology	0275-1380 0275-1380			1982	459-462		4	4
Outcome	thesis	2015	Markowitz, Tracy	Short-term Attachment Outcomes of Infants in the Child Welfare System				http://aura.a	2015		47		
Outcome	thesis	2003	Markus, Donna Rose	Addiction, attachment and social support				http://libpro	2003		208		
Outcome	thesis	2007	Marotti, Alessandra	Life events concentration around the exacerbation of substance abuse in adolescents				http://libpro	2007		134		
No Control Group	thesis	2003	Maroufi, Hengameh	The development of prenatally drug -exposed children: A comparison between those living with their mothers and those placed in foster care				http://libpro	2003		128		
Outcome	thesis	2015	Marshall, Keith L.	Exploring Developmental Risk Factors Associated with Adolescent Substance Abuse				http://libpro	2015		209		
Age	thesis	2000	Martin, Nancy Ann	Effects of prenatal drug exposure on cognitive development and erps in 5 to 7 year old children.				http://libpro	2000-02		3773		
Outcome	thesis	2012	Martin, Yolanda C.	Wasting away: Substance abuse and health risk outcomes among Dominican deportees				http://libpro	2012		225		
Exposure	thesis	2009	Mason, Zachary S.	The role of maternal attachment: Its effects on postpartum depression and infant social-emotional development				http://libpro	2009		85		
Age	journalArticle	2009	Matano B.A.; Heller N.A.; Paul J.A.; Krishnan R.; Hayes M.J.	Sleep, arousal and spontaneous movements in neonates of opiate addicted women	Developmental Psychobiology	0012-1630	10.1002/dev	http://www	2009	594		7	51
Age	journalArticle	2003	Mathew P.J.; Mathew J.L.	Assessment and management of pain in infants	Postgraduate Medical Journal	0032-5473	10.1136/pmj	http://www	2003	438-443		934	79
Age	journalArticle	2003	Mathew P.J.; Mathew J.L.	Pain in the neonate: Appreciation, assessment and alleviation	Perinatology	0972-2408		http://www	2003	107-120		3	5
Exposure	journalArticle	1996	Mayes L.C.	Exposure to cocaine: behavioral outcomes in preschool and school-age children.	NIDA research monograph	1046-9516		http://www	1996	211-229		(Mayes L.C.)	164
Exposure	journalArticle	1996	Mayes, L. C.	Exposure to cocaine: behavioral outcomes in preschool and school-age children.	NIDA research monograph	1046-9516 1046-9516			1996	211-229			164
Age	journalArticle	1996	Mayes, Linda C.; Carroll, Kathleen M.	Neonatal withdrawal syndrome in infants exposed to cocaine and methadone.	Substance Use & Misuse	1082-6084	10.3109/108	http://libpro	1996-01	241-253		2	31
Exposure	journalArticle	1998	Maza, P. L.; Wright, L. L.; Bauer, C. R.; Shankaran, S.; Bada, H. S.; Lester, B.; Krause-Steinrauf, H.; Smeriglio, V. L.; Bowler, A.; Katsikiotis, V.	Maternal Lifestyles Study (MLS). Caretaking environment and stability of substance-exposed infants at one month corrected age.	Annals of the New York Academy of Sciences	0077-8923 0077-8923			6/21/98	358-361			846
Study Type	journalArticle	2019	McAllister, Jennifer M.; Wexelblatt, Scott L.; Hall, Eric S.	Developmental Disorders and Medical Complications Among Infants with Subclinical Intrauterine Opioid Exposures.	Population Health Management	1942-7891	10.1089/pop	http://libpro	2019-02	19-24		1	22
Age	journalArticle	1991	McCann, E. M.; Lewis, K.	Control of breathing in babies of narcotic- and cocaine-abusing mothers.	Early human development	0378-3782 0378-3782			1991-12	175-186		3	27

© 2020 Nelson LF et al. *JAMA Network Open.*

Age	journalArticle	2015	McCarthy J.J.; Leamon M.H.; Willits N.H.; Salo R.	The effect of methadone dose regimen on neonatal abstinence syndrome	Journal of Addiction Medicine	1935-3227	10.1097/AD	http://www	2015	105-110		2	9
Outcome	thesis	2008	McClure, Candace Kelly	in women of reproductive age and during pregnancy and birth outcomes following acute poisoning hospital discharge during pregnancy				http://libpro	2008		117		
Exposure	journalArticle	2014	McCormack L.; Chen J.-W.; Trapp L.; Job A.	A comparison of sedation-related events for two multiagent oral sedation regimens in pediatric dental patients	Pediatric dentistry	1942-5473		http://www	2014	302-308		4	36
Exposure	journalArticle	1997	McElhatton P.R.; Sullivan F.M.; Volans G.N.	Paracetamol overdose in pregnancy analysis of the outcomes of 300 cases referred to the teratology information service	Reproductive Toxicology	0890-6238	10.1016/S08	http://www	1997	85-94		1	11
Outcome	journalArticle	2009	McGlade, Andrea; Ware, Robert; Crawford, Maree	Child protection outcomes for infants of substance-using mothers: a matched-cohort study.	Pediatrics	1098-4275 0	10.1542/peds.2008-0576		2009-07	285-293		1	124
Data Format	journalArticle	2015	McGlone, L.; Mactier, H.	Infants of opioid-dependent mothers: Neurodevelopment at six months	Early Human Development	0378-3782	10.1016/j.ea	http://www	2015	19-21		1	91
Outcome	journalArticle	2013	McGlone, Laura; Hamilton, Ruth; McCulloch, Daphne L.; Boulton, Richard; Bradnam, Michael S.; Weaver, Lawrence T.; Mactier, Helen	Neonatal visual evoked potentials in infants born to mothers prescribed methadone.	Pediatrics	0031-4005	10.1542/ped	http://libpro	2013-03	e857-e863		3	131
Outcome	journalArticle	2014	McGlone, Laura; Hamilton, Ruth; McCulloch, Daphne L.; MacKinnon, Jane R.; Bradnam, Michael; Mactier, Helen	Visual outcome in infants born to drug-misusing mothers prescribed methadone in pregnancy.	The British journal of ophthalmology	1468-2079 0	10.1136/bjophthalmol-20		2014-02	238-245		2	98
Study Type	journalArticle	2008	McGlone, Laura; Mactier, Helen; MacKinnon, Jane R.	Outcome in infants exposed to methadone in utero.	BMJ (Clinical research ed.)	1756-1833 0959-535X			9/23/08	a1774			337
Outcome	journalArticle	2002	McGuire W.; Fowlie P.W.	Naloxone for narcotic-exposed newborn infants.	Cochrane database of systematic reviews (Online)	1469-493X		http://www	2002	CD003483		4	
Exposure	thesis	2013	McKnight, Jacqueline Odell	African American women and substance abuse: The impact of cumulative disadvantages and attachment patterns on addiction severity and treatment participation				http://libpro	2013		128		
Exposure	thesis	2007	McMillin, Heidee Eileen	Process and outcome evaluation of the Spokane County Meth Family Treatment Court, 2003ñ2005				http://libpro	2007		257		
Outcome	thesis	1999	McNeilly, Catherine G.	Mothers' representations of their children and Axis II diagnoses in women on methadone maintenance				http://libpro	1999		160		
Exposure	thesis	1998	McNeilly, Lemmietta Garlyn Jones	A descriptive analysis of the receptive and expressive language skills of young children born to mothers with human immunodeficiency virus infection				http://libpro	1998		159		
Outcome	journalArticle	1989	McPherson D.L.; Madden J.D.; Payne T.F.	Auditory brainstem-evoked potentials in term infants born to mothers addicted to opiates.	Journal of perinatology : official journal of the California Perinatal Association	0743-8346		http://www	1989	262-267		3	9
Age	journalArticle	2015	McQueen, Karen A.; Murphy-Oikonen, Jodie; Desaulniers, Lindsay	Maternal Substance Use and Neonatal Abstinence Syndrome: A Descriptive Study.	Maternal and child health journal	1573-6628 1	10.1007/s10995-015-1689		2015-08	1756-1765		8	19
Age	journalArticle	2011	McQueen, Karen A.; Murphy-Oikonen, Jodie; Gerlach, Keri; Montelpare, William	The impact of infant feeding method on neonatal abstinence scores of methadone-exposed infants.	Advances in neonatal care : official journal of the National Association of Neonatal Nurses	1536-0911 1	10.1097/ANC.0b013e3182		2011-08	282-290		4	11
Exposure	journalArticle	2017	Mehrnoush N.; Ashktorab T.; Heidarzadeh M.; Momenzadeh S.	Knowledge and attitude of personnel, key factors in implementation of neonatal pain management in NICU: A qualitative study	Journal of Clinical and Diagnostic Research	0973-709X	10.7860/JCD	http://www	2017	SC05-SC09		11	11
Outcome	journalArticle	2013	Melinder, A.; Konijnenberg, C.; Sarfi, M.	Deviant smooth pursuit in preschool children exposed prenatally to methadone or buprenorphine and tobacco affects integrative visuomotor capabilities.	Addiction (Abingdon, England)	1360-0443 0	10.1111/add.12267		2013	2175-2182		12	108
Outcome	thesis	2009	Mendoza, Natasha S.	Single mothers, substance misuse and child well-being: Examining the effects of family structure and service provision in the child welfare system				http://libpro	2009		253		
Exposure	thesis	2011	Merrill, Lisa Dawn	Demographic and Psychosocial Correlates of Illicit Drug Use in Pregnancy: A Mixed Methods Study				http://libpro	2011		264		
Exposure	journalArticle	2014	Messerer, B.; Groegl, G.; Stromer, W.; Jaksch, W.	Pediatric perioperative systemic pain therapy. Austrian interdisciplinary recommendations on pediatric perioperative pain management	Schmerz	0932-433X	10.1007/s00482-013-1384		2014-02	43-64		1	28
Exposure	journalArticle	2004	Seifer, Ron; Lester, Barry M.; Lagasse, Linda L.; Wright, Linda L.; Shankaran, Seetha; Bada, Henrietta S.; Smeriglio, Vincent L.; Langer, John C.; Beeghly,	The maternal lifestyle study: cognitive, motor, and behavioral outcomes of cocaine-exposed and opiate-exposed infants through three years of age	Pediatrics	1098-4275			2004-06	1677-1685		6	113

© 2020 Nelson LF et al. *JAMA Network Open.*

Exposure	journalArticle	1980	Metroka D.C.; Marchesani J.R.; Carrel R.	A submucous technique utilizing a narcotic and a potentiator.	The Journal of pedodontics	0145-5508		http://www	1980	124-138		2	4
Exposure	thesis	2010	Meyer, Andrea Selaine	Risk and protective factors for substance use among adolescents involved with the child welfare system				http://libpro	2010		130		
Exposure	thesis	2002	Meyers, Ruth A. M.	The experiences of women with AIDS who have received occupational therapy intervention in a home health care setting				http://libpro	2002		286		
Exposure	journalArticle	2003	Miaskowski C.	Identifying issues in the management of pain in infants and children	Pain Management Nursing	1524-9042	10.1053/jpm	http://www	2003	2-Jan		1	4
Exposure	journalArticle	2003	Mikhel'son V.A.; Zhirkova I.V.; Beliaeva I.D.; Stepanenko S.M.; Manerova A.F.; Butyleva O.I.	Postoperative analgesia with tramal in newborn children using the method of continuous intravenous infusion	Anesteziologiia i reanimatologiia	0201-7563		http://www	2003	24-28		1	
Exposure	journalArticle	1997	Miller H.	Prenatal cocaine exposure and mother-infant interaction: implications for occupational therapy intervention.	The American journal of occupational therapy. : official publication of the American Occupational Therapy Association	0272-9490		http://www	1997	119-131		2	51
Exposure	journalArticle	2005	Ronald; Lagasse, Linda L.; Bauer, Charles R.; Shankaran, Seetha; Bada, Henrietta S.; Wright, Linda L.; Smeriglio, Vincent L.; Bigsby, Rosemarie; Liu, Jing	Predictors of motor development in children prenatally exposed to cocaine.	Neurotoxicology and Teratology	0892-0362	10.1016/j.nt	http://libpro	2005-03	213-220		2	27
Exposure	journalArticle	2000	Milnes A.R.; MaupomÈ G.; Cannon J.	Intravenous sedation in pediatric dentistry using midazolam, nalbuphine and droperidol.	Pediatric dentistry	0164-1263		http://www	2000	113-119		2	22
Study Type	journalArticle	2011	Minnes, Sonia; Lang, Adelaide; Singer, Lynn	Prenatal tobacco, marijuana, stimulant, and opiate exposure: outcomes and practice implications.	Addiction science & clinical practice	1940-0640 1940-0632			2011-07	57-70		1	6
Outcome	journalArticle	2013	Moe-Byrne T.; Brown J.V.; McGuire W.	Naloxone for opiate-exposed newborn infants.	Cochrane database of systematic reviews (Online)	1469-493X		http://www	2013	CD003483		(Moe-Byrne	2
Exposure	journalArticle	2002	Moe, Vibeke	Foster-placed and adopted children exposed in utero to opiates and other substances: prediction and outcome at four and a half years	Journal of developmental and behavioral pediatrics: JDBP	0196-206X			2002-10	330-339		5	23
Outcome	journalArticle	2010	Moller M.; Karaskov T.; Koren G.	Opioid detection in maternal and neonatal hair and meconium: Characterization of an at-risk population and implications to fetal toxicology	Therapeutic Drug Monitoring	0163-4356	10.1097/FTD	http://www	2010	318-323		3	32
Outcome	journalArticle	2018	Alan; Cabez, Manuel Blesa; Cooper, E. Sarah; Mactier, Helen; Semple, Scott I.; Bastin, Mark E.; Boardman, James P.	Prenatal methadone exposure is associated with altered neonatal brain development.	NeuroImage. Clinical	2213-1582 2	10.1016/j.nicl.2017.12.03		2018	14-Sep			18
Exposure	journalArticle	2000	Moore, P. A.; Cuddy, M. A.; Magera, J. A.; Caputo, A. C.; Chen, A. H.; Wilkinson, L. A.	Oral transmucosal fentanyl pretreatment for outpatient general anesthesia.	Anesthesia progress	0003-3006 0003-3006			2000	29-34		2	47
Exposure	journalArticle	2017	Morag I.; Rotem I.; Frisch M.; Hendler I.; Simchen M.J.; Leibovitz L.; Maayan-Metzger A.; Strauss T.	Cumulative pain-related stress and developmental outcomes among low-risk preterm infants at one year corrected age	Early Human Development	1872-6232	10.1016/j.ea	http://www	2017	5-Jan		(Morag I., Iri	109
Exposure	thesis	2017	Morgan-Eason, Andrea	The Process of Mothering: Women in Recovery from Drug Addiction				http://libpro	2017		190		
Outcome	journalArticle	2019	Morie, Kristen P.; Crowley, Michael J.; Mayes, Linda C.; Potenza, Marc N.	Prenatal drug exposure from infancy through emerging adulthood: Results from neuroimaging	Drug and Alcohol Dependence	0376-8716	10.1016/j.dr	http://libpro	5/1/19	39-53			198
Outcome	journalArticle	2012	Morley, Stephen R.; Becker, Julia; Al-Adnani, Mudher; Cohen, Marta Cecilia	Drug- and alcohol-related deaths at a pediatric institution in the United kingdom.	The American journal of forensic medicine and pathology	1533-404X 0	10.1097/PAF.0b013e3182		2012-12	390-394		4	33
Outcome	thesis	2012	Morris, Traci	Economic status and substance abuse during pregnancy				http://libpro	2012		34		
Study Type	journalArticle	1986	Morselli P.L.	Influence of development on the pharmacokinetics of drugs in neonates, infants and children	Annales de Pediatrie	0066-2097		http://www	1986	151-160		2	33
Outcome	thesis	2009	Mosing, Patricia R.	Maternal perceptions of their infants: Do perceptions predict maternal motivation to nurture?				http://libpro	2009		178		
Study Type	journalArticle	2004	Moskalenko, V. D.	Psychophysical development of children exposed antenatally to narcotic drugs. Russian.	Zhurnal nevrologii i psikhiatrii imeni S.S. Korsakova	1997-7298 1997-7298			2004	65-68		12	104
Outcome	journalArticle	2013	Mostrom, Krister	[The importance of early and good intervention].	Tidsskrift for den Norske laegeforening : tidsskrift for praktisk medicin, ny raekke	0807-7096 0	10.4045/tidsskr.13.0643		6/11/13	1161		11	133

© 2020 Nelson LF et al. *JAMA Network Open.*

Outcome	journalArticle	1991	Moyer SMR; Howe CJ	Pediatric pain intervention in the PACU.	Critical Care Nursing Clinics of North America	0899-5885		http://libpro	1991-03	49-57		1	3
Study Type	journalArticle	1998	Msall, M. E.; Bier, J. A.; LaGasse, L.; Tremont, M.; Lester, B.	The vulnerable preschool child: The impact of biomedical and social risks on neurodevelopmental function	Seminars in Pediatric Neurology	1071-9091			1998-03	52-61		1	5
Outcome	thesis	2000	Mulia, Nina Sandra	Everyday struggles for well -being among low -income, drug -using women				http://libpro	2000		241		
Outcome	journalArticle	2007	Mulvihill, Alan O.; Cackett, Peter D.; George, Nick D.; Fleck, Brian W.	Nystagmus secondary to drug exposure in utero.	The British journal of ophthalmology	0007-1161 0	10.1136/bjo.2006.105569		2007-05	613-615		5	91
Age	journalArticle	2010	Murphy-Oikonen, Jodie; Montelpare, William J.; Southon, Sarah; Bertoldo, Larry; Persichino, Nancy	Identifying infants at risk for neonatal abstinence syndrome: a retrospective cohort comparison study of 3 screening approaches.	The Journal of perinatal & neonatal nursing	1550-5073 0	10.1097/JPN.0b013e3181f		2010-12	366-372		4	24
Outcome	thesis	2004	Murphy, Anne	Daughters' perspectives on maternal substance abuse: Pledge to be a different kind of mother. A grounded theory study				http://libpro	2004		226		
Exposure	journalArticle	2002	Murray D.J.; Cole J.W.; Shrock C.D.; Snider R.J.; Martini J.A.	Sevoflurane versus halothane: Effect of oxycodone premedication on emergence behaviour in children	Paediatric Anaesthesia	1155-5645	10.1046/j.14	http://www.	2002	308-312		4	12
Exposure	journalArticle	2003	Musial, Kimberly M.; Wilson, Stephen; Preisch, James; Weaver, Joel	Comparison of the efficacy of oral midazolam alone versus midazolam and meperidine in the pediatric dental patient.	Pediatric dentistry	0164-1263 0164-1263			2003-10	468-474		5	25
Outcome	journalArticle	2015	N¯rgaard M.; Nielsson M.S.; Heide-J¯rgensen U.	Birth and neonatal outcomes following opioid use in pregnancy: A danish population-based study	Substance Abuse: Research and Treatment	1178-2218	10.4137/SaR	http://www.	2015	11-May		(N¯rgaard M	9
KT4XW86J	journalArticle	2008	Nair, Prasanna; Black, Maureen M; Ackerman, John P; Schuler, Maureen E; Keane, Virginia	Childrenís Cognitive-Behavioral Functioning at Age 6 and 7: Prenatal Drug Exposure and Caregiving Environment	Ambulatory pediatrics : the official journal of the	1530-1567	10.1016/j.a	hhttps://www	2008	154-162		3	8
Exposure	journalArticle	2002	Nathan J.E.; Vargas K.G.	Oral midazolam with and without meperidine for management of the difficult young pediatric dental patient: a retrospective study.	Pediatric dentistry	0164-1263		http://www.	2002	129-138		2	24
Exposure	journalArticle	1987	Nathan J.E.; West M.S.	Comparison of chloral hydrate-hydroxyzine with and without meperidine for management of the difficult pediatric patient.	ASDC journal of dentistry for children			http://www.	1987	437-444		6	54
Outcome	thesis	2011	Nathan, Aleah L.	The power of love: Attachment style in the battered woman syndrome				http://libpro	2011		160		
Outcome	thesis	2018	Neary, Karen D.	Perinatal Nurses' Therapeutic Attitudes towards Women Who Use Addictive Substances during Pregnancy				http://libpro	2018		162		
Outcome	thesis	2016	Nelson, Brittany L.	Parent-child interaction therapy: A psychoeducational group model for substance using mothers in a residential perinatal substance abuse treatment program				http://libpro	2016		124		
Outcome	journalArticle	2004	Neu N; Leighty R; Adeniyi-Jones S; Diaz C; Handelsman E; Kaufman G; Paul ME; Rich K; Mofenson L; Pitt J	Immune parameter and morbidity in hard drug and human immunodeficiency virus-exposed but uninfected infants.	Pediatrics	0031-4005		http://libpro	2004-05	1260-1266		5	113
Exposure	journalArticle	2017	Monique van; Rosmalen, Joost van; Standing, Joseph F.; Sheng, Yucheng; Rascher, Wolfgang; Roberts, Deborah; Winslade, Jackie; Rawcliffe, Louise; Hanning,	for SEDation of paediatric patients in the intensive care unit: study protocol for a multicentre randomised controlled trial.	BMJ open	2044-6055 2	10.1136/bmjopen-2017-0		6/21/17	e016031		6	7
Outcome	journalArticle	2016	Newman R.G.; Gevertz S.G.	Stigmatizing and Misleading Terminology - More	Journal of Addiction Medicine	1935-3227	10.1097/AD	http://www.	2016	208		3	10
Outcome	journalArticle	2015	Newman, Adam; Davies, Gregory A.; Dow, Kimberly; Holmes, Belinda; Macdonald, Jessica; McKnight, Sarah; Newton, Lynn	Rooming-in care for infants of opioid-dependent mothers: Implementation and evaluation at a tertiary care hospital.	Canadian family physician Medecin de famille canadien	1715-5258 0008-350X			2015-12	e555-561		12	61
Study Type	journalArticle	1985	Newman, R. G.	Offspring born to methadone-maintained women.	The American journal of drug and alcohol abuse	0095-2990 0095-2990			1985	163-167		2-Jan	11
Outcome	journalArticle	2005	Niccols A.; Sword W.	"New Choices" for substance-using mothers and their children: Preliminary evaluation	Journal of Substance Use	1465-9891	10.1080/146	http://www.	2005	239-251		4	10
Study Type	journalArticle	1973	Nichtern S.	The children of drug users.	Journal of the American Academy of Child Psychiatry	0002-7138		http://www.	1973	24-31		1	12
Study Type	journalArticle	2013	Niesters, M.; Overdyk, F.; Smith, T.; Aarts, L.; Dahan, A.	Opioid-induced respiratory depression in paediatrics: a review of case reports	British Journal of Anaesthesia	0007-0912	10.1093/bja/aes447		2013-02	175-182		2	110

© 2020 Nelson LF et al. *JAMA Network Open.*

Exposure	journalArticle	2012	Nizhnikov M.E.; Pautassi R.M.; Varlinskaya E.I.; Spear N.E.	Prenatal ethanol exposure affects intake and motivational properties of ethanol during infancy: Possible mechanisms of action	Alcoholism: Clinical and Experimental Research	0145-6008	10.1111/j.15	http://www	2012	65A		(Nizhnikov M	36
Exposure	thesis	2001	Noel, Pamela E.	Case management in treatment for adolescent substance use: An approach to program evaluation				http://libpro	2001		299		
Outcome	journalArticle	1992	NYBERG, K.; ALLEBECK, P.; EKLUND, G.; JACOBSON, B.	SOCIOECONOMIC VERSUS OBSTETRIC RISK-FACTORS FOR DRUG-ADDICTION IN OFFSPRING	British Journal of Addiction	0952-0481			1992-12	1669-1676		12	87
Exposure	journalArticle	2015	Nygaard, Egil; Moe, Vibeke; Slinning, Kari; Walhovd, Kristine B.	Longitudinal cognitive development of children born to mothers with opioid and polysubstance use.	Pediatric research	1530-0447 0	10.1038/pr.2015.95		2015-09	330-335		3	78
Age	journalArticle	2018	Nygaard, Egil; Slinning, Kari; Moe, Vibeke; Due-T¯nnessen, Paulina; Fjell, Anders; Walhovd, Kristine B.	Neuroanatomical characteristics of youths with prenatal opioid and poly-drug exposure	Neurotoxicology and Teratology	0892-0362	10.1016/j.nt	http://libpro	2018-07	13-26			68
Age	journalArticle	2016	Nygaard, Egil; Slinning, Kari; Moe, Vibeke; Walhovd, Kristine B.	Behavior and Attention Problems in Eight-Year-Old Children with Prenatal Opiate and Poly-Substance Exposure: A Longitudinal Study.	PloS one	1932-6203 1	10.1371/journal.pone.015		2016	e0158054		6	11
Age	journalArticle	2013	O'Connor A.B.; O'Brien L.; Alto W.A.	Are there gender related differences in neonatal abstinence syndrome following exposure to buprenorphine during pregnancy?	Journal of Perinatal Medicine	0300-5577	10.1515/jpm	http://www	2013	621-623		5	41
Study Type	journalArticle	2013	O'Grady K.E.; Jones H.E.; Jansson L.M.; Kaltenbach K.	Response to Wouldes and Woodward: Maternal methadone dose during pregnancy and infant clinical outcome	Neurotoxicology and Teratology	0892-0362	10.1016/j.nt	http://www	2013	116-118		(O'Grady K.E	39
Exposure	journalArticle	2001	Oakes LL	Assessment and management of pain in the critically ill pediatric patient.	Critical Care Nursing Clinics of North America	0899-5885		http://libpro	2001-06	281-295		2	13
Exposure	journalArticle	2000	Oberlander TF; Robeson P; Ward V; Huckin RS; Kamani A; Harpur A; McDonald W	Prenatal and breast milk morphine exposure following maternal intrathecal morphine treatment.	Journal of Human Lactation	0890-3344		http://libpro	2000	137-142		2	16
Study Type	journalArticle	2016	Obladen, Michael	Lethal Lullabies: A History of Opium Use in Infants.	Journal of human lactation : official journal of International Lactation Consultant Association	1552-5732 0	10.1177/08903344155946		2016-02	75-85		1	32
Outcome	journalArticle	2016	Oconnor A.B.; Obrien L.; Alto W.A.; Wong J.	Does concurrent in utero exposure to buprenorphine and antidepressant medications influence the course of neonatal abstinence syndrome?	Journal of Maternal-Fetal and Neonatal Medicine	1476-4954	10.3109/147	http://www	2016	112-114		1	29
Outcome	journalArticle	2009	Oei, Ju Lee; Abdel-Latif, Mohamed E.; Craig, Fiona; Kee, Aivy; Austin, Marie-Paule; Lui, Kei	Short-term outcomes of mothers and newborn infants with comorbid psychiatric disorders and drug dependency.	Australian and New Zealand Journal of Psychiatry	0004-8674	10.1080/000	http://libpro	2009	323-331		4	43
Outcome	journalArticle	2017	Nadin; Breen, Courtney; Burns, Lucinda; Hilder, Lisa; Bajuk, Barbara; Abdel-Latif, Mohamed E.; Ward, Meredith; Feller, John M.; Falconer, Janet; Clews,	Neonatal Abstinence Syndrome and High School Performance	Pediatrics	0031-4005	10.1542/peds.2016-2651		2017-02	e20162651		2	139
Outcome	thesis	2008	Ogilvie, Kristen Aubrey	Where science meets community: An Alaskan case study of a community -centered approach in prevention research				http://libpro	2008		250		
Outcome	journalArticle	2017	OíConnor, Alane B.; Kelly, Brandon K.; OíBrien, Liam M.; O'Connor, Alane B; O'Brien, Liam M	Maternal and infant outcomes following third trimester exposure to marijuana in opioid dependent pregnant women maintained on buprenorphine.	Drug & Alcohol Dependence	0376-8716	10.1016/j.dr	http://libpro	2017-11	200-203			180
Outcome	journalArticle	2016	Oji?Mmuo, Christiana N.; Michael, Eric J.; McLatchy, Jacqueline; Lewis, Mary M.; Becker, Julie E.; Doheny, Kim Kopenhaver	Skin conductance at baseline and postheel lance reflects sympathetic activation in neonatal opiate withdrawal.	Acta Paediatrica	0803-5253	10.1111/apa	http://libpro	2016-03	e99-e106		3	105
Study Type	journalArticle	2017	Okoroh, Ekwutosi M.; Gee, Rebekah E.; Jiang, Baogong; McNeil, Melissa B.; Hardy-Decuir, Beverly A.; Zapata, Amy L.	Neonatal abstinence syndrome: Trend and expenditure in Louisiana Medicaid, 2003ñ2013.	Maternal and Child Health Journal	1092-7875	10.1007/s10	http://libpro	2017-07	1479-1487		7	21
Outcome	journalArticle	1999	Olsen Jÿ.; Steffensen F.H.; Czeicel A.; Nielsen G.L.; De Jong L.; Irgens L.; Bergman U.; Pedersen L.; Lie R.T.; De Vries C.; Leurquin P.	Prescribing during pregnancy and lactation with reference to the Swedish classification system. A population-based study among Danish women	Acta Obstetricia et Gynecologica Scandinavica	0001-6349		http://www	1999	686-692		8	78
Outcome	thesis	2018	Olivieri, Georgianna	Burden of Worthlessness: The Role of Familial Shame in Opioid Addiction				http://libpro	2018		61		
No Control Group	journalArticle	1983	Olofsson M.; Buckley W.; Andersen G.E.; Friis Hansen B.	Investigation of 89 children born by drug-dependent mothers. II. Follow-up 1-10 years after birth	Acta Paediatrica Scandinavica	0001-656X		http://www	1983	407-410		3	72
Exposure	journalArticle	2003	Ornoy A.	environment in neurodevelopmental toxicity: Long-term neurobehavioral studies in children at risk for developmental disorders	Toxicology Letters	0378-4274	10.1016/S03	http://www	2003	171-181		(Ornoy A., o	140-141
Exposure	journalArticle	1996	Ornoy, A.; Michailevskaya, V.; Lukashov, I.; Bar-Hamburger, R.; Harel, S.	The developmental outcome of children born to heroin-dependent mothers, raised at home or adopted	Child Abuse & Neglect	0145-2134			1996-05	385-396		5	20

© 2020 Nelson LF et al. *JAMA Network Open.*

Exposure	journalArticle	2001	Ornoy, A.; Segal, J.; Bar-Hamburger, R.; Greenbaum, C.	Developmental outcome of school-age children born to mothers with heroin dependency: importance of environmental factors.	Developmental medicine and child neurology	0012-1622 0012-1622			2001-10	668-675		10	43
Exposure	journalArticle	2016	Ornoy, Asher; Finkel-Pekarsky, Victoria; Peles, Einat; Adelson, Miriam; Schreiber, Shaul; Ebstein, P. Richard	ADHD risk alleles associated with opiate addiction: study of addicted parents and their children.	Pediatric research	1530-0447 0	10.1038/pr.2016.78		2016-08	228-236		2	80
Outcome	journalArticle	2012	Ortigosa S.; Friguls B.; Joya X.; Martinez S.; MariÒoso M.L.; Alameda F.; Vall O.; Garcia-Algar O.	Feto-placental morphological effects of prenatal exposure to drugs of abuse	Reproductive Toxicology	0890-6238	10.1016/j.re	http://www	2012	73-79		1	34
Outcome	thesis	2007	Ortiz, Lisa M.	Educational attainment among high -risk teenage mothers				http://libpro	2007		97		
Outcome	journalArticle	1998	Ostrea E.M. Jr.; Matias O.; Keane C.; Mac E.; Utarnachitt R.; Ostrea A.; Mazhar M.	Spectrum of gestational exposure to illicit drugs and other xenobiotic agents in newborn infants by meconium analysis	Journal of Pediatrics	0022-3476	10.1016/S00	http://www	1998	513-515		4	133
Outcome	journalArticle	1997	Ostrea, E. M. Jr; Ostrea, A. R.; Simpson, P. M.	Mortality within the first 2 years in infants exposed to cocaine, opiate, or cannabinoid during gestation.	Pediatrics	1098-4275 0031-4005			1997-07	79-83		1	100
Exposure	journalArticle	2011	Ozer L, Oktem ZB, Küçükyavuz Z.	Effects of deep sedation on behaviors and side effects in children undergoing different dental procedures	Pediatric Dentistry	0164-1263		http://www	2011	158-164		2	33
Outcome	journalArticle	1989	Pacifico P.; Nardelli E.; Pantarotto M.F.	Neonatal heroin withdrawal syndrome; Evaluation of different pharmacological treatments	Pharmacological Research	0031-6989		http://www	1989	63-64		SUPPL. 1	21
Exposure	journalArticle	1973	Paik T.J.	Demerol, phenergan, and atarax premedication in dentistry for children.	Taehan Ch'ikkwa Uisa Hy?phoe chi	0376-4672		http://www	1973	483-490		7	11
Outcome	journalArticle	2013	Palchik, Alexander B.; Einspieler, Christa; Evstafeyeva, Irina V.; Talisa, Victor B.; Marschik, Peter B.	Intra-uterine exposure to maternal opiate abuse and HIV: The impact on the developing nervous system.	Early Human Development	0378-3782	10.1016/j.ea	http://libpro	2013-04	229-235		4	89
Exposure	journalArticle	2010	Pandey R.; Padmanabhan M.; Saksena A.; Chandra G.	Midazolam-fentanyl analgo-sedation in pediatric dental patients - A pilot study	Journal of Clinical Pediatric Dentistry	1053-4628		http://www	2010	105-110		1	35
Outcome	journalArticle	1988	Pasto M.; Ehrlich S.; Graziani L.; Kurtz A.; Goldberg B.; Kaltenbach K.; Finnegan L.	Cerebral sonographic characteristics and maternal and neonatal risk factors in infants of opiate dependent mothers	NIDA Research Monograph Series			http://www	1988	317		90	
Outcome	journalArticle	1984	Pasto M.; Graziani L.; Leifer B.	Cerebral ventricular changes in newborns exposed to psychoactive agents in utero	NIDA Research Monograph Series	1046-9516		http://www	1984	331		(Pasto M.; G	NO. 49
Exposure	thesis	2006	Patterson, David Allen	Motivational interviewing: Does it increase alcohol and other drug addicted clients' retention in outpatient treatment?				http://libpro	2006		104		
Outcome	journalArticle	2014	Heller, Nicole A.; Morrison, Deborah G.; Pritham, Ursula A.; Tisher, Paul W.; Troese, Marcia; Brown, Mark S.; Hayes, Marie J.	Development of auditory event-related potentials in infants prenatally exposed to methadone.	Developmental psychobiology	1098-2302 0	10.1002/dev.21160		2014-07	1119-1128		5	56
Exposure	journalArticle	1992	Payne, K. A.; Coetzee, A. R.; Mattheyse, F. J.; Heydenrych, J. J.	Behavioural changes in children following minor surgery--is premedication beneficial?	Acta anaesthesiologica Belgica	0001-5164 0001-5164			1992	173-179		3	43
Exposure	journalArticle	1999	Payne, K. A.; Roelofse, J. A.	Tramadol drops in children: analgesic efficacy, lack of respiratory effects, and normal recovery times.	Anesthesia progress	0003-3006 0003-3006			1999	91-96		3	46
Exposure	thesis	2015	Payne, Linda Gail	The experience of caring for women with drug or alcohol problems in the general hospital				http://libpro	2015		147		
Exposure	journalArticle	2003	Peters J.W.B.; Koot H.M.; De Boer J.B.; Passchier J.; Bueno-de-Mesquita J.M.; De Jong F.H.; Duivenvoorden H.J.; Tibboel D.	Major surgery within the first 3 months of life and subsequent biobehavioral pain responses to immunization at later age: A case comparison study	Pediatrics	0031-4005	10.1542/ped	http://www	2003	129-135		1	111
Exposure	journalArticle	1996	Petrack E.M.; Marx C.M.; Wright M.S.	Intramuscular ketamine is superior to meperidine, promethazine, and chlorpromazine for pediatric emergency department sedation	Archives of Pediatrics and Adolescent Medicine	1072-4710		http://www	1996	676-681		7	150
Exposure	thesis	2016	Phillips, Cindy DuPre'	Psychological and Sociocultural Influences of Current and Historical Intimate Partner Violence in Pregnancy				http://libpro	2016		167		
Outcome	journalArticle	2012	Pichini S.; Garcia-Algar O.	Hair analysis of newborns and children to determine medical and social risk: The experience of barcelona hospital del mar	Journal of Population Therapeutics and Clinical Pharmacology	1710-6222		http://www	2012	e301		2	19
Exposure	journalArticle	2014	Pichini S.; Garcia-Algar O.; Alvarez A.; Gottardi M.; Marchei E.; Svaizer F.; Pellegrini M.; Rotolo M.C.; Pacifici R.	Assessment of unsuspected exposure to drugs of abuse in children from a mediterranean city by hair testing	International Journal of Environmental Research and Public Health	1661-7827	10.3390/ijer	http://www	2014	2288-2298		2	11

© 2020 Nelson LF et al. *JAMA Network Open.*

Exposure	journalArticle	2003	Pichini S.; Pacifici R.; Pellegrini M.; Marchei E.; PÈrez-AlarcÛn E.; Puig C.; Vall O.; GarcÌa-Algar O.	Development and validation of a liquid chromatography-mass spectrometry assay for the determination of opiates and cocaine in meconium	Journal of Chromatography B: Analytical Technologies in the Biomedical and Life Sciences	1570-0232	10.1016/S15	http://www	2003	281-292		2	794
Exposure	journalArticle	2014	Pichini, Simona; Garcia-Algar, Oscar; Alvarez, Airam-Tenesor; Mercadal, Maria; Mortali, Claudia; Gottardi, Massimo; Svaizer, Fiorenza; Pacifici, Roberta	Pediatric Exposure to Drugs of Abuse by Hair Testing: Monitoring 15 Years of Evolution in Spain	International Journal of Environmental Research and Public Health	1660-4601	10.3390/ijerph110808267		2014-08	8267-8275		8	11
Sample Size	thesis	1994	Pinto, Peter F.	The effect of parent treatment environment and intrauterine exposure to heroin on the cognitive development of children of addicted parents.				http://libpro	1994		6481		
Exposure	journalArticle	1998	Pliner, V.; Weedon, J.; Thomas, P. A.; Steketee, R. W.; Abrams, E. J.; Lambert, G.; Greenberg, B.; Bamji, M.; Thea, D. M.; Matheson, P. B.	Incubation period of HIV-1 in perinatally infected children	Aids	0269-9370	10.1097/00002030-19980		5/7/98	759-766		7	12
Outcome	journalArticle	2006	Alencar D.M.; Brandalise S.R.; Guimar„es Carvalho E.; Coser V.M.; Costa I.; CÛrdoba J.C.; Emerenciano M.; Dobbin J J.; Moraes Guerra M.C.; Gumes Lopes V.;	Infant acute leukemia and maternal exposures during pregnancy	Cancer Epidemiology Biomarkers and Prevention	1055-9965	10.1158/105	http://www	2006	2336-2341		12	15
Exposure	journalArticle	2010	Pong K.M.; Abdel-Latif M.E.; Lui K.; Wodak A.D.; Feller J.M.; Campbell T.; Oei J.	The temporal influence of a heroin shortage on pregnant drug users and their newborn infants in Sydney, Australia	Australian and New Zealand Journal of Obstetrics and Gynaecology	1479-828X	10.1111/j.14	http://www	2010	230-236		3	50
Exposure	journalArticle	1990	Poorman T.L.; Farrington F.H.; Mourino A.P.	Comparison of a chloral hydrate/hydroxyzine combination with and without meperidine in the sedation of pediatric dental patients.	Pediatric dentistry	0164-1263		http://www	1990	288-291		5	12
Exposure	thesis	2010	Poznansky, Olga	Stability and change in maternal reflective functioning in early childhood				http://libpro	2010		118		
Study Type	journalArticle	1972	Priestley B.L.	Drug addiction and the newborn.	Proceedings of the Royal Society of Medicine	0035-9157		http://www	1972	870		10	65
Outcome	journalArticle	2018	Prindle J.J.; Hammond I.; Putnam-Hornstein E.	Prenatal substance exposure diagnosed at birth and infant involvement with child protective services	Child Abuse and Neglect	1873-7757	10.1016/j.ch	http://www	2018	75-83		(Prindle J.J.,	76
Exposure	journalArticle	2005	Prins S.A.; Peeters M.Y.M.; Houmes R.J.; van Dijk M.; Knibbe C.A.J.; Danhof M.; Tibboel D.	Propofol 6% as sedative in children under 2 years of age following major craniofacial surgery	British Journal of Anaesthesia	0007-0912	10.1093/bja/	http://www	2005	630-635		5	94
Outcome	journalArticle	2013	Pritham U.A.	Breastfeeding Promotion for Management of Neonatal Abstinence Syndrome	JOGNN - Journal of Obstetric, Gynecologic, and Neonatal Nursing	1552-6909	10.1111/155	http://www	2013	517-526		5	42
Study Type	journalArticle	2017	Pryor J.R.; Maalouf F.I.; Krans E.E.; Schumacher R.E.; Cooper W.O.; Patrick S.W.	The opioid epidemic and neonatal abstinence syndrome in the USA: A review of the continuum of care	Archives of Disease in Childhood: Fetal and Neonatal Edition	1468-2052	10.1136/arc	http://www	2017	F183-F187		2	102
Exposure	journalArticle	2003	Przybylo H.J.; Martini D.R.; Mazurek A.J.; Bracey E.; Johnsen L.; CotÈ C.J.	Assessing behaviour in children emerging from anaesthesia: Can we apply psychiatric diagnostic techniques?	Paediatric Anaesthesia	1155-5645	10.1046/j.14	http://www	2003	609-616		7	13
Exposure	journalArticle	2008	Pulsifer, Margaret B.; Butz, Arlene M.; O'Reilly Foran, Megan; Belcher, Harolyn M. E.	Prenatal drug exposure: effects on cognitive functioning at 5 years of age.	Clinical pediatrics	0009-9228 0	10.1177/00099228073058		2008-01	58-65		1	47
Exposure	journalArticle	2004	Pulsifer, Margaret B.; Radonovich, Krestin; Belcher, Harolyn M. E.; Butz, Arlene M.	Intelligence and School Readiness in Preschool Children With Prenatal Drug Exposure.	Child Neuropsychology	0929-7049	10.1080/092	http://libpro	2004-06	89-101		2	10
Outcome	journalArticle	2009	Quick, Zoe L.; Robb, Michael P.; Woodward, Lianne J.	Acoustic cry characteristics of infants exposed to methadone during pregnancy.	Acta Paediatrica	0803-5253	10.1111/j.16	http://libpro	2009-01	74-79		1	98
Exposure	journalArticle	2016	Radhika K.P.; Sreejit M.S.; Ramadas K.T.	Efficacy of midazolam as oral premedication in children in comparison to triclofos sodium	Indian Journal of Anaesthesia	0019-5049	10.4103/001	http://www	2016	415-419		6	60
Exposure	journalArticle	1993	Rahbar F.; Fomufod A.; White D.; Westney L.S.	Impact of intrauterine exposure to phencyclidine (PCP) and cocaine on neonates.	Journal of the National Medical Association	0027-9684		http://www	1993	349-352		5	85
Outcome	thesis	2010	Raines-Milenkov, Amy L.	Health and pregnancy among women with prostitution experience				http://libpro	2010		304		
Exposure	thesis	1996	Rainville, Alice Johannah	An investigation of the pica practices of pregnant women in Houston and Prairie View, Texas				http://libpro	1996		210		
Exposure	journalArticle	2006	Rakhmanina N.Y.; van den Anker J.N.	Pharmacological research in pediatrics: From neonates to adolescents	Advanced Drug Delivery Reviews	0169-409X	10.1016/j.ad	http://www	2006	14-Apr		1	58
No Control Group	journalArticle	1975	Ramer, C. M.; Lodge, A.	Neonatal addiction: a two-year study. Part I. Clinical and developmental characteristics of infants of mothers on methadone maintenance	Addictive Diseases	0094-0267			1975	227-234		2-Jan	2

© 2020 Nelson LF et al. *JAMA Network Open.*

Exposure	journalArticle	2013	Ranger M.; Celeste Johnston C.; Rennick J.E.; Limperopoulos C.; Heldt T.; Du Plessis A.J.	A multidimensional approach to pain assessment in critically ill infants during a painful procedure	Clinical Journal of Pain	0749-8047	10.1097/AJP	http://www	2013	613-620		7	29
Exposure	journalArticle	2001	Ransjˆ-Arvidson A.B.; Matthiesen A.S.; Lilja G.; Nissen E.; Widstrˆm A.M.; Uvn‰s-Moberg K.	Maternal analgesia during labor disturbs newborn behavior: effects on breastfeeding, temperature, and crying.	Birth (Berkeley, Calif.)	0730-7659		http://www	2001	12-May		1	28
Exposure	thesis	2017	Raphemot, Cenean Walls	Using the COMPASS-EZ and Dual Diagnosis Capability in Addiction Treatment (DDCAT) Index to Improve Outcomes: Recovering, Renewing, and Restoring Lives				http://libpro	2017		70		
Exposure	journalArticle	2001	Reimann, B.; Kretz, F. J.	Ontogenesis of anesthesia-relevant receptors	Anasthesiologie Intensivmedizin Notfallmedizin Schmerztherapie	0939-2661	10.1055/s-2001-18051		2001-11	664-682		11	36
Outcome	thesis	2006	Reinking, Maria Andrea	ìI just want a normal life!î: A phenomenological inquiry into children's perspectives on parental addiction and the effects of the addiction in the parent -child relationship				http://libpro	2006		142		
Exposure	journalArticle	2002	Religa, Zdzislaw Christopher; Wilson, Stephen; Ganzberg, Stephen I.; Casamassimo, Paul S.	Association between bispectral analysis and level of conscious sedation of pediatric dental patients.	Pediatric dentistry	0164-1263 0164-1263			2002-06	221-226		3	24
Exposure	journalArticle	2000	Renwick A.G.; Dorne J.L.; Walton K.	An analysis of the need for an additional uncertainty factor for infants and children	Regulatory Toxicology and Pharmacology	0273-2300	10.1006/rtpł	http://www	2000	286-296		3	31
Study Type	journalArticle	2009	Reynolds B.C.; Penman D.K.M.; Howatson A.G.; Jackson L.A.; Skeoch C.H.	Multifocal multi-organ ischaemia and infarction in a preterm baby due to maternal intravenous cocaine use: A case report	Journal of Medical Case Reports	1752-1947	10.1186/175	http://www	2009			1	3
Outcome	journalArticle	2007	Reynolds E.W.; Riel-Romero R.M.S.; Bada H.S.	Neonatal abstinence syndrome and cerebral infarction following maternal codeine use during pregnancy	Clinical Pediatrics	0009-9228	10.1177/000	http://www	2007	639-645		7	46
Outcome	thesis	2015	Rhea, Melissa A.	Factors affecting retention for pregnant and parenting women in residential substance abuse treatment: A retrospective study				http://libpro	2015		116		
Study Type	journalArticle	2000	Rice, D.; Barone, S.	Critical periods of vulnerability for the developing nervous system: Evidence from humans and animal models	Environmental Health Perspectives	0091-6765	10.1289/ehp.00108s3511		2000-06	511-533			108
Exposure	journalArticle	1996	Rice, L. J.	Pain management in children.	Canadian journal of anaesthesia = Journal canadien d'anesthesie	0832-610X 0832-610X			1996-05	R155-162		5 Pt 2	43
Exposure	journalArticle	2002	Richards K.A.; Stasko T.	Dermatologic surgery and the pregnant patient	Dermatologic Surgery	1076-0512	10.1046/j.15	http://www	2002	248-256		3	28
Exposure	journalArticle	1981	Richards M.P.M.	Effects of analgesics and anaesthetics given in childbirth on child development	Neuropharmacology	0028-3908		http://www	1981	1259-1265		12 B	20
Exposure	journalArticle	1996	Richardson, Gale A.; Hamel, Sara C.; Goldschmidt, Lidush; Day, Nancy L.	The effects of prenatal cocaine use on neonatal neurobehavioral status.	Neurotoxicology and Teratology	0892-0362	10.1016/089	http://libpro	1996-09	519-528		5	18
Exposure	thesis	2001	Rigney, Betty Ann	readiness for behavior change and continued substance abuse in mothers who have used illegal drugs during pregnancy				http://libpro	2001		109		
Exposure	journalArticle	2013	Ritwik P.; Cao L.T.; Curran R.; Musselman R.J.	Post-sedation events in children sedated for dental care.	Anesthesia progress	0003-3006	10.2344/000	http://www	2013	54-59		2	60
Exposure	journalArticle	1991	Rivera WB	Practical points in the assessment and management of postoperative pediatric pain.	Journal of Post Anesthesia Nursing	0883-9433		http://libpro	1991-02	40-42		1	6
Outcome	thesis	2014	Roberson, Emily K.	Using pregnancy risk assessment monitoring system data to investigate prescription drug use during pregnancy in Hawai`i				http://libpro	2014		99		
Exposure	journalArticle	1992	Roberts S.M.; Wilson C.F.; Seale N.S.; McWhorter A.G.	Evaluation of morphine as compared to meperidine when administered to the moderately anxious pediatric dental patient.	Pediatric dentistry	0164-1263		http://www	1992	306-313		5	14
Exposure	thesis	2006	Roberts, Chastity N.	Applying the theory of planned behavior to the intent to quit smoking among pregnant women				http://libpro	2006		122		
Outcome	thesis	2009	Roberts, Sarah Carolyn	drug use in prenatal care: The (not so) hidden connections between universal screening and reporting to Child Protective Services				http://libpro	2009		181		
Outcome	thesis	2019	Robertson, Kirsten	Introducing Shame Resilience to Women Who Struggle with Complex Trauma and Substance Abuse				http://libpro	2019		159		

© 2020 Nelson LF et al. *JAMA Network Open.*

Exposure	thesis	2009	Robinson, Carleen	The relational theory of substance abuse and African American women who are HIV+				http://libpro	2009		181		
Exposure	thesis	2018	Rodriguez-Manning, Susan	Client Demographic Factors, Failed Urinalysis, and Treatment Completion at a Substance Abuse Treatment Facility				http://libpro	2018		161		
Exposure	journalArticle	1999	Roelofse, J. A.; Payne, K. A.	Oral tramadol: analgesic efficacy in children following multiple dental extractions.	European journal of anaesthesiology	0265-0215 0265-0215			1999-07	441-447		7	16
Exposure	journalArticle	1996	Rogers, W. B.	Colic is pain.	Pediatrics	0031-4005 0031-4005			1996-04	601-602		4	97
Exposure	journalArticle	2013	Rogosnitzky, Moshe; Finegold, Milton J.; McLaughlin, Patricia J.; Zagon, Ian S.	Opioid growth factor (OGF) for hepatoblastoma: a novel non-toxic treatment	Investigational New Drugs	0167-6997	10.1007/s10637-012-9918		2013-08	1066-1070		4	31
Outcome	thesis	2009	Romero, Valeria I.	Parental substance abuse and child neglect: A controlled trial of a developed treatment manual				http://libpro	2009		169		
Outcome	journalArticle	1989	Rosati P.; Noia G.; Conte M.; De Santis M.; Mancuso S.	Drug abuse in pregnancy: fetal growth and malformations.	Panminerva medica	0031-0808		http://www	1989	71-75		2	31
Outcome	thesis	2000	Rosen, Daniel	Partner violence in the lives of low -income teenage mothers				http://libpro	2000		253		
Included	journalArticle	1988	Rosen, T. S.; Johnson, H. L.	Drug-addicted mothers, their infants, and SIDS.	Annals of the New York Academy of Sciences	0077-8923	10.1111/j.17	http://libpro	1988	89-95			533
Included	journalArticle	1985	Rosen, T. S.; Johnson, H. L.	Long-term effects of prenatal methadone maintenance.	NIDA research monograph	1046-9516 1046-9516			1985	73-83			59
Included	journalArticle	1982	Rosen, T. S.; Johnson, H. L.	Children of methadone-maintained mothers: follow-up to 18 months of age	The Journal of Pediatrics	0022-3476			1982	192-196		2	101
Study Type	journalArticle	1993	Rosen, Tove S.; Johnson, Helen L.	Prenatal methadone maintenance: Its effects on fetus, neonate, and child.	Developmental Brain Dysfunction	1019-5815		http://libpro	1993-11	317-323		6	6
Age	journalArticle	2011	Roy, J.; Toubin, R.-M.; Mazurier, E.; Chanal, C.; Misraoui, M.; Brulet, C.; Molenat, F.	Developmental outcome of 5-year-old children born to opiate-dependent mothers: Effects of a multidisciplinary intervention during pregnancy	Archives De Pediatrie	0929-693X	10.1016/j.arcped.2011.08.		2011-11	1130-1138		11	18
Outcome	journalArticle	2019	Rubenstein, Eric; Young, Jessica C.; Croen, Lisa A.; DiGuiseppi, Carolyn; Dowling, Nicole F.; Lee, Li-Ching; Schieve, Laura; Wiggins, Lisa D.; Daniels, Julie	Brief Report: Maternal Opioid Prescription from Preconception Through Pregnancy and the Odds of Autism Spectrum Disorder and Autism Features in Children.	Journal of autism and developmental disorders	1573-3432 0	10.1007/s10803-018-3721		2019-01	376-382		1	49
Outcome	thesis	2013	Rubio, Melissa	The experiences of women entering methadone treatment for opioid use: An interpretive phenomenological inquiry				http://libpro	2013		151		
Outcome	thesis	2009	Russo, Jessica	substance abusing mothers using Bowen Family Systems concepts based on the Personal Authority in Family Systems Questionnaire				http://libpro	2009		139		
Outcome	journalArticle	2016	Saeidi R.; Farkhani E.M.; Saeidi M.; Izadi S.; Lotfabadi M.Z.	Neonatal effects of substance abuse during pregnancy	Iranian Journal of Neonatology	2322-2158		http://www	2016	40-43		2	7
Outcome	journalArticle	2001	Salem M.Y.; Ross S.A.; Murphy T.P.; ElSohly M.A.	GC-MS determination of heroin metabolites in meconium: Evaluation of four solid-phase cartridges	Journal of Analytical Toxicology	0146-4760		http://www	2001	93-98		2	25
Exposure	journalArticle	1995	Sallee F.R.; Katikaneni L.P.; McArthur P.D.; Ibrahim H.M.; Nesbitt L.; Sethuraman G.	Head growth in cocaine-exposed infants: relationship to neonate hair level.	Journal of developmental and behavioral pediatrics : JDBP	0196-206X		http://www	1995	77-81		2	16
Outcome	journalArticle	2009	Salo S; Kivistˆ K; Korja R; Biringen Z; Tupola S; Kahila H; Kivitie-Kallio S	Emotional availability, parental self-efficacy beliefs, and child development in caregiver-child relationships with buprenorphine-exposed 3-year-olds.	Parenting: Science & Practice	1529-5192		http://libpro	2009-07	244-259		4-Mar	9
Included	journalArticle	2010	Salo, S.; Politi, J.; Tupola, S.; Biringen, Z.; Kalland, M.; Halmesm‰ki, E.; Kahila, H.; Kivitie?Kallio, S.	Early development of opioid?exposed infants born to mothers in buprenorphine?replacement therapy	Journal of Reproductive and Infant Psychology	0264-6838, 1	10.1080/026	http://www	2010	161-179		2	28
Exposure	journalArticle	1993	Sams D.R.; Cook E.W.; Jackson J.G.; Roebuck B.L.	Behavioral assessments of two drug combinations for oral sedation.	Pediatric dentistry	0164-1263		http://www	1993	186-190		3	15
Outcome	thesis	2003	Sandau-Beckler, Patricia Ann	A description of the readiness for change among substance abusing mothers in the child welfare system				http://libpro	2003		254		

© 2020 Nelson LF et al. *JAMA Network Open.*

Exposure	journalArticle	1997	Sanders, B. J.; Avery, D. R.	The effect of sleep on conscious sedation: a follow-up study.	The Journal of clinical pediatric dentistry	1053-4628 1053-4628			1997	131-134		2	21
No Control Group	journalArticle	2009	Sandtorv L.; Reigstad H.; Bruar¯y S.; Elgen I.; Laegreid L.M.	Substitution treatment of drug addicts during pregnancy: consequences for the children?	Tidsskrift for den Norske lÊgeforening : tidsskrift for praktisk medicin, ny rÊkke	0807-7096		http://www	2009	287-290		4	129
Age	journalArticle	2018	Sandtorv, Lisbeth Beate; Fevang, Silje Katrine Elgen; Nilsen, Sondre Aasen; B¯e, Tormod; Gjestad, Rolf; Haugland, Siren; Elgen, Irene Bircow	Deficit/Hyperactivity Disorder and Autism Spectrum Disorders in School-Aged Children Prenatally Exposed to Substances.	Substance Abuse: Research & Treatment	1178-2218	10.1177/117	http://libpro	2018-01	1-Jan			12
Outcome	journalArticle	2009	Sarfi, M.; Martinsen, H.; Bakstad, B.; Roislien, J.; Waal, H.	Patterns in sleep-wakefulness in three-month old infants exposed to methadone or buprenorphine.	Early Human Development	1872-6232 0	10.1016/j.earlhumdev.200		2009	773-778		12	85
Outcome	journalArticle	2011	Sarfi, M.; Smith, L.; Waal, H.; Sundet, J. M.	Risks and realities: Dyadic interaction between 6-month-old infants and their mothers in opioid maintenance treatment	Infant Behavior and Development	0163-6383	10.1016/j.inf	http://www	2011	578-589		4	34
Outcome	journalArticle	2013	Sarfi, M.; Sundet, J. M.; Waal, H.	Maternal stress and behavioral adaptation in methadone- or buprenorphine-exposed toddlers	Infant Behavior and Development	0163-6383	10.1016/j.inf	http://www	2013	707-716		4	36
Study Type	journalArticle	2006	Sarkar S.; Donn S.M.	Management of neonatal abstinence syndrome in neonatal intensive care units: A national survey	Journal of Perinatology	0743-8346	10.1038/sj.jp	http://www	2006	15-17		1	26
Exposure	journalArticle	1974	Scanlon J.W.	Obstetric anesthesia as a neonatal risk factor in normal labor and delivery.	Clinics in perinatology	0095-5108		http://www	1974	465-482		2	1
Outcome	thesis	1998	Schenberg, Timothy Brian	Children of substance abusers: The relationship of perceived parental rejection to child emotional functioning				http://libpro	1998		278		
Outcome	journalArticle	1988	Scherer P.	Methadone maintenance has a tragic price.	The American journal of nursing	0002-936X		http://www	1988	1630, 1632		12	88
Study Type	journalArticle	2005	Schindler, Andreas; Thomasius, Rainer; Sack, Peter-Michael; Gemeinhardt, Brigitte; K¸stner, Udo; Eckert, Jochen	Attachment and substance use disorders: A review of the literature and a study in drug dependent adolescents.	Attachment & Human Development	1461-6734	10.1080/146	http://libpro	2005-09	207-228		3	7
Outcome	journalArticle	1996	Schneider, J. W.; Hans, S. L.	Effects of prenatal exposure to opioids on focused attention in toddlers during free play.	Journal of developmental and behavioral pediatrics : JDBP	0196-206X 0196-206X			1996	240-247		4	17
Unable to Locate	thesis	1993	Schneider, Jane W.	An investigation of focused attention in opioid-exposed toddlers.				http://libpro	1993		2425		
Exposure	journalArticle	2003	Schuler, Maureen E.; Nair, Prasanna; Kettinger, Laurie	Drug-exposed infants and developmental outcome: effects of a home intervention and ongoing maternal drug use	Archives of Pediatrics & Adolescent Medicine	1072-4710			2003-02	133-138		2	157
Exposure	thesis	2016	Schultz, Katie A'Neil	Native Women, Intimate Partner Violence, and Drug Use and Consequences: Prevalence and Associations among Tribal College and University Students				http://libpro	2016		155		
Outcome	thesis	1998	Schulz, Linda Lao	Concomitants of success in a perinatal substance abuse treatment program				http://libpro	1998		227		
Outcome	journalArticle	1998	Scott, C. S.; Decker, J. L.; Edwards, M. L.; Freid, E. B.	Withdrawal after narcotic therapy: a survey of neonatal and pediatric clinicians.	Pharmacotherapy	0277-0008 0277-0008			1998-12	1308-1312		6	18
Outcome	thesis	2008	Sealy, Peter Ashward	Racial oppression and the link to mental illness in Blacks and the substance abuse factor				http://libpro	2008		274		
Outcome	journalArticle	2004	Seifer, Ronald; LaGasse, Linda L.; Lester, Barry; Bauer, Charles R.; Shankaran, Seetha; Bada, Henrietta S.; Wright, Linda L.; Smeriglio, Vincent L.; Liu, Jing	Attachment Status in Children Prenatally Exposed to Cocaine and Other Substances.	Child Development	0009-3920	10.1111/j.14	http://libpro	2004-05	850-868		3	75
Exposure	journalArticle	2011	Seo I.-S.; Seong C.-R.; Jung G.; Park S.-J.; Kim S.Y.; Kim M.M.	The effect of sub-Tenon lidocaine injection on emergence agitation after general anaesthesia in paediatric strabismus surgery	European Journal of Anaesthesiology	0265-0215	10.1097/EJA	http://www	2011	334-339		5	28
Study Type	journalArticle	2008	Serane, V. Tiroumourougane; Kurian, Ommen	Neonatal abstinence syndrome.	Indian journal of pediatrics	0973-7693 0	10.1007/s12098-008-0107		2008-09	911-914		9	75
Outcome	thesis	2006	Sexton, Chris Collier	Axis I and Axis II psychopathology among at-risk mothers: Correlates of parenting quality and child adjustment				http://libpro	2006		159		
Outcome	thesis	2014	Shaddix, Catherine	An interpretative phenomenological study of the experience of parents who attended a mindfulness-based childbirth and parenting program				http://libpro	2014		287		

© 2020 Nelson LF et al. *JAMA Network Open.*

Outcome	thesis	2013	Shadur, Julia Madeleine	Parent emotion socialization and emotion regulation in substance abusing families				http://libpro	2013		114		
Exposure	journalArticle	2011	Shankaran S.; Das A.; Bauer C.R.; Bada H.S.; Lester B.M.; Wright L.L.; Higgins R.D.; Poole W.K.	Prenatal cocaine exposure and small-for-gestational-age status: Effects on growth at 6years of age	Neurotoxicology and Teratology	0892-0362	10.1016/j.nt	http://www	2011	575-581		5	33
Exposure	journalArticle	2006	Sheroan, Marianne M.; Dilley, Diane C.; Lucas, Warner J.; Vann, William F.	A prospective study of 2 sedation regimens in children: chloral hydrate, meperidine, and hydroxyzine versus midazolam, meperidine, and hydroxyzine.	Anesthesia progress	0003-3006 0	10.2344/0003-3006(2006)		2006	83-90		3	53
Exposure	thesis	2001	Shipley, Stacey Lynn	Attachment, psychopathy, and women who commit predatory homicide: A case study investigation				http://libpro	2001		399		
Exposure	journalArticle	2010	Shor, Sarit; Nulman, Irena; Kulaga, Vivian; Koren, Gideon	Heavy in utero ethanol exposure is associated with the use of other drugs of abuse in a high-risk population	Alcohol	0741-8329	10.1016/j.alcohol.2009.08		2010-12	623-627		8-Jul	44
Outcome	thesis	2017	Siminerio Lemon, Lara	Pharmacotherapy in Pregnancy: Application to Opioid Maintenance Therapy and 17-OHPC for Prevention of Preterm Birth				http://libpro	2017		135		
Exposure	journalArticle	2000	Simmons S	Pain management in pediatric patients.	Journal of Vascular Access Devices	1083-0081		http://libpro	2000	15-Oct		1	5
Exposure	journalArticle	2012	Singer L.T.; Moore D.G.; Fulton S.; Goodwin J.; Turner J.J.D.; Min M.O.; Parrott A.C.	Neurobehavioral outcomes of infants exposed to MDMA (Ecstasy) and other recreational drugs during pregnancy	Neurotoxicology and Teratology	0892-0362	10.1016/j.nt	http://www	2012	303-310		3	34
Outcome	journalArticle	2009	Skinner, Martie L.; Haggerty, Kevin P.; Fleming, Charles B.; Catalano, Richard F.	Predicting functional resilience among young-adult children of opiate-dependent parents.	Journal of Adolescent Health	1054-139X	10.1016/j.ja	http://libpro	2009-03	283-290		3	44
Outcome	journalArticle	1997	Skopp, G.; Potsch, L.	A case report on drug screening of nail clippings to detect prenatal drug exposure.	Therapeutic drug monitoring	0163-4356 0163-4356			1997-08	386-389		4	19
Exposure	journalArticle	2016	Skovlund E.; Handal M.; Selmer R.M.; Brandlistuen R.E.; Skurtveit S.	Language development in 3 year old children after prenatal exposure to opioids	Pharmacoepidemiology and Drug Safety	1099-1557	10.1002/pds	http://www	2016	17		(Skovlund E.	25
Exposure	journalArticle	2017	Skovlund, Eva; Handal, Marte; Selmer, Randi; Brandlistuen, Ragnhild Eek; Skurtveit, Svetlana	Language competence and communication skills in 3-year-old children after prenatal exposure to analgesic opioids	Pharmacoepidemiology and Drug Safety	1053-8569	10.1002/pds.4170		2017-06	625-634		6	26
Outcome	journalArticle	2019	Skurtveit, Svetlana; Nechansk·, Blanka; Handal, Marte; Mahic, Milada; Mrav?Ìk, Viktor; GabrhelÌk, Roman	Hospitalization of children after prenatal exposure to opioid maintenance therapy during pregnancy: a national registry study from the Czech Republic.	Addiction	0965-2140	10.1111/add	http://libpro	2019-07	1225-1235		7	114
Outcome	thesis	2007	Slocum, Lee Ann	General strain theory and stability in offending and substance use over time: A dynamic approach				http://libpro	2007		197		
Exposure	thesis	2003	Sloss, Christine Miriam Faust	The mental health needs and service utilization of women who trade sex				http://libpro	2003		297		
Study Type	journalArticle	1999	Smeriglio, V. L.; Wilcox, H. C.	Prenatal drug exposure and child outcome. Past, present, future.	Clinics in perinatology	0095-5108 0095-5108			1999-03	16-Jan		1	26
Exposure	journalArticle	2012	Smith L.M.; Lagasse L.L.; Derauf C.; Newman E.; Shah R.; Arria A.; Huestis M.A.; Haning W.; Strauss A.; Dellagrotta S.; Dansereau L.M.; Neal C.; Lester B.M.	Growth and neurodevelopmental outcomes in children prenatally exposed to methamphetamine	Birth Defects Research Part A - Clinical and Molecular Teratology	1542-0752	10.1002/bdr	http://www	2012	303		5	94
Outcome	thesis	2012	Smith, Courtney E.	Indirect screening: Enhancing identification of illicit drug use during pregnancy				http://libpro	2012		108		
Exposure	journalArticle	2011	Newman, Elana; Shah, Rizwan; Haning, William; Arria, Amelia; Huestis, Marilyn; Strauss, Arthur; Della Grotta, Sheri; Dansereau, Lynne M.; Lin, Hai; Lester,	Motor and cognitive outcomes through three years of age in children exposed to prenatal methamphetamine.	Neurotoxicology and Teratology	0892-0362	10.1016/j.nt	http://libpro	2011-01	176-184		1	33
Exposure	thesis	2014	Snyder, Melia Ann	pregnant, post-partum, and parenting women in recovery from substance abuse: An expressive arts group therapy intervention				http://libpro	2014		229		
Exposure	journalArticle	1994	Soepatmi, S.	Developmental outcomes of children of mothers dependent on heroin or heroin/methadone during pregnancy	Acta Paediatrica (Oslo, Norway: 1992). Supplement	0803-5326			1994-11	36-39			404
Exposure	journalArticle	2003	Song, Yun U.; Webb, Michael D.	Comparison of the effect of orally versus submucosally administered meperidine on the behavior of pediatric dental patients: a retrospective study.	Anesthesia progress	0003-3006 0003-3006			2003	129-133		3	50
Study Type	journalArticle	1974	Soule A.B.; Standley K.; Copans S.A.; Davis M.	Clinical uses of the Brazelton Neonatal Scale.	Pediatrics	0031-4005		http://www	1974	583-586		5	54

© 2020 Nelson LF et al. *JAMA Network Open.*

Study Type	thesis	2016	Southgate, Karl	A Potential Space: Discovering a Place for D.W. Winnicott in the Psychoanalytic Literature on Drug Addiction				http://libpro	2016		304		
Exposure	thesis	2007	Spears, Gwendolyn Verdelle	An examination of psychosocial, behavioral and sociodemographic factors associated with substance use among pregnant adolescents				http://libpro	2007		168		
Outcome	journalArticle	2017	Spehr, Michelle K.; Coddington, Jennifer; Ahmed, Azza H.; Jones, Elizabeth	Parental Opioid Abuse: Barriers to Care, Policy, and Implications for Primary Care Pediatric Providers.	Journal of pediatric health care : official publication of National Association of Pediatric Nurse Associates & Practitioners		10.1016/j.pedhc.2017.05.		2017-07				
Outcome	thesis	2019	Sperandio, Katharine	When a Man Loves a Woman: The Lived Experiences of Male Sober Partners in Romantic Relationships with Women Who Struggle with Addiction				http://libpro	2019		209		
Outcome	journalArticle	1982	Spotts, J. V.; Shontz, F. C.	Ego development, dragon fights, and chronic drug abusers.	The International journal of the addictions	0020-773X 0020-773X			1982-08	945-976		6	17
Outcome	journalArticle	2017	Stabler, Meagan; Giacobbi, Peter Jr; Chertok, Ilana; Long, Leann; Cottrell, Lesley; Yossuck, Panitan	Comparison of Biological Screening and Diagnostic Indicators to Detect In Utero Opiate and Cocaine Exposure Among Mother-Infant Dyads.	Therapeutic drug monitoring	1536-3694 0	10.1097/FTD.0000000000		2017-12	640-647		6	39
Exposure	journalArticle	2010	Stade B.C.; Bennett D.; Khuu M.; Patterson K.; Tran S.; Kapur B.	Endogenous methanol derived formic acid corelates with cognitive dysfunction in children born to drinking mot hers	Paediatrics and Child Health	1751-7222		http://www	2010	70A-71A		(Stade B.C.;	15
Outcome	journalArticle	2002	Stanger C; Kamon J; Dumenci L; Higgins ST; Bickel WK; Grabowski J; Amass L	Predictors of internalizing and externalizing problems among children of cocaine and opiate dependent parents.	Drug & Alcohol Dependence	0376-8716		http://libpro	2002-04	199-212		2	66
Age	journalArticle	2007	Steinhausen, H.-C.; Blattmann, B.; Pfund, F.	Developmental outcome in children with intrauterine exposure to substances	European Addiction Research	1022-6877	10.1159/000097939		2007	94-100		2	13
Outcome	thesis	2018	Stephens, Donna	Early Assessment of the Pregnant Substance Abuser				http://libpro	2018		51		
Study Type	journalArticle	1998	Stevens B	ketorolac, and sucrose reduce postoperative and procedural pain in children [commentary on Maikler VE. Pharmacologic pain management in children: a review of	Evidence Based Nursing	1367-6539		http://libpro	1998-10	114-114			
Study Type	journalArticle	2001	Stevens B.J.; Franck L.S.	Assessment and management of pain in neonates	Paediatric Drugs	1174-5878		http://www	2001	539-558		7	3
Outcome	thesis	2009	Stevenson, Lauren DeMarco	The influence of treatment motivation, treatment status and social networks on perceived social support of women with substance use or co-occurring disorders				http://libpro	2009		184		
Outcome	journalArticle	1976	STIMMEL, B.; ADAMSONS, K.	NARCOTIC DEPENDENCY IN PREGNANCY - METHADONE-MAINTENANCE COMPARED TO USE	Jama-Journal of the American Medical Association	0098-7484	10.1001/jama.235.11.112		1976	1121-1124		11	235
Outcome	journalArticle	2010	Stone, Kristen C.; LaGasse, Linda L.; Lester, Barry M.; Shankaran, Seetha; Bada, Henrietta S.; Bauer, Charles R.; Hammond, Jane A.	Sleep problems in children with prenatal substance exposure: the Maternal Lifestyle study.	Archives of pediatrics & adolescent medicine	1538-3628 1	10.1001/archpediatrics.20		2010-05	452-456		5	164
Outcome	thesis	2014	Stone, Rebecca Jane Grainger	A narrative inquiry of women's substance use in pregnancy and motherhood				http://libpro	2014		206		
Outcome	thesis	2011	Stoops, Brandi Sue	Voices from the underworld: The shadow world of addiction				http://libpro	2011		310		
Outcome	thesis	2002	Storer, Elena	Identifying mothers at risk for child abuse through the development of a maternal prenatal assessment instrument				http://libpro	2002		187		
Outcome	thesis	2012	Stortz, Jessica Nichole	Spatial analysis of pregnancy complications associated with maternal cardiovascular disease risk in Ontario				http://libpro	2012		141		
Outcome	journalArticle	2015	Stover, Megan W.; Davis, Jonathan M.	Opioids in pregnancy and neonatal abstinence syndrome.	Seminars in perinatology	1558-075X 0	10.1053/j.semperi.2015.0		2015-11	561-565		7	39
Included	journalArticle	1979	Strauss, M. E.; Lessenfirestone, J. K.; Chavez, C. J.; Stryker, J. C.	Children of methadone-treated women at 5 years of age	Pharmacology Biochemistry and Behavior	0091-3057			1979	6-Mar			11
Study Type	journalArticle	1983	Strauss, M. E.; Reynolds, K. S.	Psychological characteristics and development of narcotic-addicted infants	Drug and Alcohol Dependence	0376-8716			1983-12	381-393		4	12
Included	journalArticle	1976	Strauss, M. E.; Starr, R. H.; Ostrea, E. M.; Chavez, C. J.; Stryker, J. C.	Behavioural concomitants of prenatal addiction to narcotics.	The Journal of pediatrics	0022-3476 0022-3476			1976-11	842-846		5	89

© 2020 Nelson LF et al. *JAMA Network Open.*

Study Type	journalArticle	1997	Strauss, R. S.	Effects of the intrauterine environment on childhood growth	British Medical Bulletin	0007-1420			1997-01	81-95		1	53
Outcome	journalArticle	1992	Struthers, Jean M.; Hansen, Robin L.	Visual recognition memory in drug-exposed infants.	Journal of Developmental and Behavioral Pediatrics	0196-206X	10.1097/000	http://libpro	1992-04	108-111		2	13
Outcome	journalArticle	2013	Sublett, Juli	Neonatal abstinence syndrome: therapeutic interventions.	MCN. The American journal of maternal child nursing	1539-0683 0	10.1097/NMC.0b013e318		2013-03	102-7; quiz 107-109		2	38
Outcome	journalArticle	2017	Suchman, Nancy E.; DeCoste, Cindy L.; McMahon, Thomas J.; Dalton, Rachel; Mayes, Linda C.; Borelli, Jessica	Mothering from the Inside Out: Results of a second randomized clinical trial testing a mentalization-based intervention for mothers in addiction treatment	Development and psychopathology	0954-5794	10.1017/S09	http://www	2017-05	617-636		2	29
No Control Group	journalArticle	1984	Suffet, Frederic; Brotman, Richard	A comprehensive care program for pregnant addicts: Obstetrical, neonatal, and child development outcomes.	International Journal of the Addictions	0020-773X	10.3109/108	http://libpro	1984-04	199-219		2	19
Age	journalArticle	2013	Sundelin Wahlsten, Viveka; Sarman, Ihsan	Neurobehavioural development of preschool-age children born to addicted mothers given opiate maintenance treatment with buprenorphine during pregnancy	Acta Paediatrica (Oslo, Norway: 1992)	1651-2227	10.1111/apa.12210		2013-05	544-549		5	102
Exposure	journalArticle	2001	Suraseranivongse S.; Santawat U.; Kraiprasit K.; Petcharatana S.; Prakkamodom S.; Muntraporn N.	Cross-validation of a composite pain scale for preschool children within 24 hours of surgery	British Journal of Anaesthesia	0007-0912	10.1093/bja/	http://www	2001	400-405		3	87
Outcome	journalArticle	2014	Sutter M.B.; Leeman L.; Hsi A.	Neonatal opioid withdrawal syndrome	Obstetrics and Gynecology Clinics of North America	1558-0474	10.1016/j.og	http://www	2014	317-334		2	41
Outcome	journalArticle	2017	Swain J.; Ho S.	Postpartum depression and opiate exposure affect maternal brain physiology according to parenting measures	Neuropsychopharmacology	1740-634X	10.1038/npp	http://www	2017	S283		(Swain J.; Ho	43
Outcome	thesis	2003	Swihart, Gayla	Female offenders: Attachment and parenthood				http://libpro	2003		223		
Outcome	journalArticle	1985	Szeto H.H.; Umans J.G.	Pharmacodynamics of fetal exposure to narcotics	NIDA Research Monograph Series	1046-9516		http://www	1985	78-87		(Szeto H.H.;	NO. 60
Outcome	thesis	2006	Ta, Van M.	Depressive symptoms and utilization of services among Asian Pacific Islander women				http://libpro	2006		310		
Exposure	journalArticle	2010	Tait A.R.; Voepel-Lewis T.; Burke C.; Doherty T.	Anesthesia induction, emergence, and postoperative behaviors in children with attention-deficit/hyperactivity disorders	Paediatric Anaesthesia	1155-5645	10.1111/j.14	http://www	2010	323-329		4	20
Exposure	thesis	2013	Tavlarides, Andrea M.	Understanding well-being in appalachian women: A qualitative study of health perceptions				http://libpro	2013		138		
Outcome	thesis	2007	Telfeyan, N. Lael	The attachment experiences of women with young children in women's substance abuse treatment: An exploratory study				http://libpro	2007		215		
Study Type	journalArticle	2016	Terasaki, Laurne S.; Gomez, Julie; Schwarz, Jaclyn M.	An examination of sex differences in the effects of early-life opiate and alcohol exposure	Philosophical Transactions of the Royal Society B-Biological Sciences	0962-8436	10.1098/rstb.2015.0123		2/19/16	20150123		1688	371
Exposure	journalArticle	2013	Thompson, Cecilia; Shabanova, Veronika; Giuliano, John S. Jr	The SNAP index does not correlate with the State Behavioral Scale in intubated and sedated children.	Paediatric anaesthesia	1460-9592 1	10.1111/pan.12258		2013-12	1174-1179		12	23
Exposure	thesis	2013	Thompson, Matthew L.	Increasing the equitability of substance abuse services for fathers involved in the child welfare system: A grant proposal				http://libpro	2013		95		
Exposure	journalArticle	1984	Thompson, R. G.	Hyperventilation, hypokalemia, and SIDS.	Hospital practice (Office ed.)	8750-2836 8750-2836			1984-12	84E-84H, 84L, 84P-84Q pa		12	19
Exposure	thesis	2012	Tillman, Shaquita	Influence of social support and intimate partner abuse on African American mothers' substance use				http://libpro	2012		63		
Study Type	journalArticle	2013	Tinelli, Francesca; Gamucci, Alessandra; Battini, Roberta; Cioni, Giovanni	Congenital nystagmus in two infants born from mothers exposed to methadone during pregnancy.	Italian journal of pediatrics	1824-7288 1	10.1186/1824-7288-39-40		7/3/13	40			39
Data Unavailable	poster abstract	2011	Tisher P.; Paul J.A.; Matano B.A.; Krishnan R.; Heller N.; Brown M.; Hayes M.J.	Maine infants-at-risk project	Journal of Neuropsychiatry and Clinical Neurosciences	0895-0172		http://www	2011	20-21		2	23
Outcome	journalArticle	2018	Tolia V.N.; Murthy K.; Bennett M.M.; Miller E.S.; Benjamin D.K.; Smith P.B.; Clark R.H.	Antenatal methadone vs buprenorphine exposure and length of hospital stay in infants admitted to the intensive care unit with neonatal abstinence syndrome	Journal of Perinatology	1476-5543	10.1038/jp.2	http://www	2018	75-79		1	38

© 2020 Nelson LF et al. *JAMA Network Open.*

Age	journalArticle	2008	Topley, J.; Windsor, D.; Williams, R.	Behavioural, developmental and child protection outcomes following exposure to Class A drugs in pregnancy	Child Care Health and Development	0305-1862	10.1111/j.1365-2214.2007		2008-01	71-76		1	34
Outcome	thesis	2018	Toquinto, Signy Marie	Pregnant Women's Acceptability of Substance Use Screening and Willingness to Disclose Use in Prenatal				http://libpro	2018		94		
Outcome	thesis	2011	Torrey, Antonia Rae	instrument to assess the treatment fidelity of a brief opportunistic intervention to reduce substance use among pregnant women				http://libpro	2011		170		
Outcome	journalArticle	2019	Towers, Craig V.; Hyatt, Branson W.; Visconti, Kevin C.; Chernicky, Lindsey; Chattin, Katie; Fortner, Kimberly B.	Neonatal Head Circumference in Newborns With Neonata Abstinence Syndrome.	Pediatrics	0031-4005	10.1542/ped	http://libpro	2019-01	7-Jan		1	143
Exposure	thesis	2007	Trabold, Nicole	The interrelationship between intimate partner violence and postpartum depression in a sample of women living in an impoverished section of an urban community				http://libpro	2007		141		
Outcome	thesis	2019	Trainor, Kristin Elise	Prenatal Substance Misuse: Exploring Healthcare Providers' Attitudes and Perceptions				http://libpro	2019		127		
Exposure	journalArticle	1999	Tronick E.Z.; Beeghly M.	Prenatal cocaine exposure, child development, and the compromising effects of cumulative risk	Clinics in Perinatology	0095-5108		http://www	1999	151-171		1	26
Outcome	journalArticle	1996	Tronick E.Z.; Lester B.M.	The NICU Network Neurobehavioral Scale: a comprehensive instrument to assess substance-exposed and high-risk infants.	NIDA research monograph	1046-9516		http://www	1996	198-204		(Tronick E.Z.	166
Exposure	journalArticle	2005	Tronick, E. Z.; Messinger, D. S.; Weinberg, M. K.; Lester, B. M.; Lagasse, L.; Seifer, R.; Bauer, C. R.; Shankaran, S.; Bada, H.; Wright, L. L.; Poole, K.; Liu, J.	of infants' and mothers' social-emotional behavior and dyadic features of their interaction in the face-to-face still-face paradigm.	Developmental psychology	0012-1649 0	10.1037/0012-1649.41.5.7		2005-09	711-722		5	41
Exposure	journalArticle	1999	Trottier, G.; Srivastava, L.; Walker, C. D.	Etiology of infantile autism: a review of recent advances in genetic and neurobiological research	Journal of Psychiatry & Neuroscience	1180-4882			1999-03	103-115		2	24
Outcome	journalArticle	2003	Tsvetkova S.; Kovachev E.; Chernookova V.	Perinatal problems in drug addiction	Akusherstvo i ginekologiia	0324-0959		http://www	2003	12-Sep		(Tsvetkova S	42 Suppl 2
Outcome	thesis	2000	Turnbull, Lorna Anne	Bearing children, bearing burdens				http://libpro	2000		222		
Outcome	journalArticle	2015	Lisa; Bajuk, Barbara; Breen, Courtney; Abdel-Latif, Mohamed E.; Feller, John M.; Falconer, Janet; Clews, Sarah; Eastwood, John; Oei, Ju Lee	Reasons for Rehospitalization in Children Who Had Neonatal Abstinence Syndrome	Pediatrics	0031-4005	10.1542/peds.2014-2767		2015-10	E811-E820		4	136
Outcome	thesis	2015	Urgelles, Jessica Marie	Examining the role of supportive others in substance abuse treatment and child welfare				http://libpro	2015		86		
Outcome	journalArticle	1995	Valentine, J. L.; Komoroski, E. M.	Use of a visual panel detection method for drugs of abuse: clinical and laboratory experience with children and adolescents.	The Journal of pediatrics	0022-3476 0022-3476			1995-01	135-140		1	126
Outcome	journalArticle	1998	Valverde Blanco F.; Moreno J.C.; Velez A.; Cano A.	Aplasia cutis	Medicina Cutanea Ibero-Latino-Americana	0210-5187		http://www	1998	274-278		5	26
Age	journalArticle	1994	Van Baar A.L.; Soepatmi S.; Gunning W.B.; Akkerhuis G.W.	Development after prenatal exposure to cocaine, heroin and methadone	Acta Paediatrica, International Journal of Paediatrics, Supplement	0803-5326		http://www	1994	40-46		404	83
Included	journalArticle	1990	van Baar, A.	Development of infants of drug dependent mothers.	Child Psychology & Psychiatry & Allied Disciplines	0021-9630	10.1111/j.14	http://libpro	1990	911-920		6	31
Study Type	journalArticle	1993	van Baar, A. L.; Boer, K.; Soepatmi, S.	[The consequences for the child of drug addiction in the mother: current status in relation to knowledge and management policy in The Netherlands].	Nederlands tijdschrift voor geneeskunde	0028-2162 0028-2162			9/4/93	1811-1815		36	137
Age	journalArticle	1989	van Baar, A. L.; Fleury, P.; Soepatmi, S.; Ultee, C. A.; Wesselman, P. J.	Neonatal behavior after drug dependent pregnancy.	Archives of disease in childhood	1468-2044 0003-9888			1989-02	235-240		2	64
Included	journalArticle	1989	van Baar, A. L.; Fleury, P.; Ultee, C. A.	Behaviour in first year after drug dependent pregnancy.	Archives of disease in childhood	1468-2044 0003-9888			1989	241-245		2	64
Included	journalArticle	1994	van Baar, A.; de Graaff, B. M.	Cognitive development at preschool-age of infants of drug-dependent mothers	Developmental Medicine and Child Neurology	0012-1622			1994-12	1063-1075		12	36
Exposure	journalArticle	2001	Van Dijk M.; De Boer J.B.; Koot H.M.; Duivenvoorden H.J.; Passchier J.; Bouwmeester N.; Tibboel D.	The association between physiological and behavioral pain measures in 0- to 3-year-old Infants after major surgery	Journal of Pain and Symptom Management	0885-3924	10.1016/S08	http://www	2001	600-609		1	22

© 2020 Nelson LF et al. *JAMA Network Open.*

Study Type	journalArticle	1990	Van Dyke D.C.; Fox A.A.	Fetal drug exposure and its possible implications for learning in the preschool and school-age population.	Journal of learning disabilities	0022-2194		http://www	1990	160-163		3	23
Outcome	thesis	2017	Van Scoyoc, Amanda	Pathways to Substance Abuse Treatment Success in Pregnancy				http://libpro	2017		157		
Outcome	journalArticle	1997	Vance, J. C.; Chant, D. C.; Tudehope, D. I.; Gray, P. H.; Hayes, A. J.	Infants born to narcotic dependent mothers: physical growth patterns in the first 12 months of life.	Journal of paediatrics and child health	1034-4810 1034-4810			1997-12	504-508		6	33
Study Type	journalArticle	2009	Vani S.; Thakre R.; Nimbalkar S.	Research issues in assessment and management of pain in newborns	Journal of Neonatology	0973-2179		http://www	2009	358-364		4	23
Outcome	thesis	2015	Vazquez, Roger D.	Evaluating overall success and relative influence of different treatment services in substance use treatment				http://libpro	2015		140		
Outcome	thesis	2001	Vedder, Julie Ann	Modifying mothers: The rhetorical construction of prenatal substance use in American discourse				http://libpro	2001		294		
Study Type	journalArticle	2018	Veeravagu A.; Azad T.D.; Jiang B.; Edwards M.S.B.	Spontaneous Intrauterine Depressed Skull Fractures: Report of 2 Cases Requiring Neurosurgical Intervention and Literature Review	World Neurosurgery	1878-8769	10.1016/j.w	http://www	2018	256-262		(Veeravagu	110
Exposure	journalArticle	2010	Vel·zquez I.; MuÒoz-Garrido J.C.	Premedication in paediatric anaesthesia: Oral transmucosal fentanyl citrate vs oral midazolam	Revista de la Sociedad Espanola del Dolor	1134-8046	10.1016/S11	http://www	2010	139-146		3	17
Age	journalArticle	2009	Velez M.L.; Jansson L.M.; Schroeder J.; Williams E.	Prenatal methadone exposure and neonatal neurobehavioral functioning	Pediatric Research	0031-3998	10.1203/PDR	http://www	2009	704-709		6	66
Outcome	journalArticle	2004	Velez, M. L.; Jansson, L. M.; Montoya, I. D.; Schweitzer, W.; Golden, A.; Svikis, D.	Parenting knowledge among substance abusing women in treatment	Journal of Substance Abuse Treatment	0740-5472	10.1016/j.jsat.2004.07.00		2004-10	215-222		3	27
Exposure	journalArticle	2016	Vet N.J.; De Wildt S.N.; Verlaat C.W.M.; Mooij M.G.; Tibboel D.; De Hoog M.; Buysse C.M.P.	Short-Term Health-Related Quality of Life of Critically Ill Children Following Daily Sedation Interruption?	Pediatric Critical Care Medicine	1947-3893	10.1097/PCC	http://www	2016	e513-e520		11	17
Exposure	journalArticle	2005	Vidal M.A.; CalderÛn E.; MartÌnez E.; Gonz·lvez A.; Torres L.M.	Pain in neonates	Revista de la Sociedad Espanola del Dolor	1134-8046		http://www	2005	98-111		2	12
Outcome	journalArticle	2003	Vinner E.; Vignau J.; Thibault D.; Codaccioni X.; Brassart C.; Humbert L.; Lhermitte M.	Neonatal hair analysis contribution to establishing a gestational drug exposure profile and predicting a withdrawal syndrome	Therapeutic Drug Monitoring	0163-4356	10.1097/000	http://www	2003	421-432		4	25
Outcome	thesis	1999	Vitucci, Judith Suzanne	Mediated learning playgroups as a tool for improving interactions between drug-exposed infants and their mothers				http://libpro	1999		233		
Exposure	journalArticle	2016	Vlenterie R.; Wood M.E.; Brandlistuen R.E.; Roeleveld N.; van Gelder M.M.H.J.; Nordeng H.	Neurodevelopmental problems at 18 months among children exposed to paracetamol in utero: A propensity score matched cohort study	International Journal of Epidemiology	1464-3685	10.1093/ije/	http://www	2016	1998-2008		6	45
Study Type	thesis	1999	Vollmer, VaNessa Ann	The effects of substance abuse on psychosocial development: A critical review of the literature				http://libpro	1999		82		
Outcome	thesis	2011	Vrieze, Danielle Marie	The Role of Parental Reflective Functioning in Promoting Attachment for Children of Depressed Mothers in a Toddler-Parent Psychotherapeutic Intervention				http://libpro	2011		95		
Exposure	journalArticle	2018	Wachman, E. M.; Hayes, M. J.; Shrestha, H.; Nikita, F. N. U.; Nolin, A.; Hoyo, L.; Daigle, K.; Jones, H. E.; Nielsen, D. A.	Epigenetic variation in OPRM1 gene in opioid-exposed mother-infant dyads.	Genes, brain, and behavior	1601-183X 1	10.1111/gbb.12476		2018-09	e12476		7	17
Outcome	journalArticle	2015	Wachman, Elisha M.; Hayes, Marie J.; Sherva, Richard; Brown, Mark S.; Davis, Jonathan M.; Farrer, Lindsay A.; Nielsen, David A.	Variations in opioid receptor genes in neonatal abstinence syndrome.	Drug & Alcohol Dependence	0376-8716	10.1016/j.dr	http://libpro	2015-10	253-259			155
Outcome	journalArticle	2011	Wachman, Elisha M.; Newby, P. K.; Vreeland, Joy; Byun, John; Bonganzi, Anthony; Bauchner, Howard; Philipp, Barbara L.	The relationship between maternal opioid agonists and psychiatric medications on length of hospitalization for neonatal abstinence syndrome.	Journal of Addiction Medicine	1932-0620	10.1097/AD	http://libpro	2011-12	293-299		4	5
Outcome	journalArticle	2018	Wachman, Elisha M.; Warden, A. Hutcheson; Thomas, Zoe; Thomas-Lewis, Jo Ann; Shrestha, Hira; Nikita, F. N. U.; Shaw, Daniel; Saia, Kelley; Schiff, Davida M.	Impact of psychiatric medication co-exposure on Neonatal Abstinence Syndrome severity.	Drug and alcohol dependence	1879-0046 0	10.1016/j.drugalcdep.201		11/1/18	45-50			192
Exposure	journalArticle	1989	Wachsman, Laura; Schuetz, Sally; Chan, Linda S.; Wingert, Willis A.	What happens to babies exposed to phencyclidine (PCP) in utero?	The American Journal of Drug and Alcohol Abuse	0095-2990	10.3109/009	http://libpro	1989	31-39		1	15
Age	journalArticle	2013	Wahlsten, Viveka Sundelin; Sarman, Ihsan	Neurobehavioural development of preschool?age children born to addicted mothers given opiate maintenance treatment with buprenorphine during pregnancy	Acta Paediatrica	0803-5253	10.1111/apa	http://libpro	2013-05	544-549		5	102

© 2020 Nelson LF et al. *JAMA Network Open.*

Outcome	thesis	2001	Wainwright, Jerina	Exploring the long -term benefits of ìspecializedî treatment services for mothers in recovery				http://libpro	2001		177		
Outcome	journalArticle	2007	Walhovd, K. B.; Moe, V.; Slinning, K.; Due-Tonnessen, P.; Bjornerud, A.; Dale, A. M.; van der Kouwe, A.; Quinn, B. T.; Kosofsky, B.; Greve, D.; Fischl, B.	Volumetric cerebral characteristics of children exposed to opiates and other substances in utero.	NeuroImage	1053-8119 1	10.1016/j.neuroimage.20		7/15/07	1331-1344		4	36
Exposure	journalArticle	2015	Kristin; Siqveland, Torill; Slinning, Kari; Nygaard, Egil; Fjell, Anders M.; Due-Tonnessen, Paulina; Bjornerud, Atle; Moe, Vibeke	Child neuroanatomical, neurocognitive, and visual acuity outcomes with maternal opioid and polysubstance detoxification.	Pediatric neurology	1873-5150 0	10.1016/j.pediatrneurol.2		2015-03	326-332.e1-3		3	52
Outcome	journalArticle	2012	Walhovd, Kristine B.; Watts, Richard; Amlien, Inge; Woodward, Lianne J.	Neural Tract Development of Infants Born to Methadone-Maintained Mothers	Pediatric Neurology	0887-8994	10.1016/j.pe	http://www	7/1/12	6-Jan		1	47
Outcome	thesis	2001	Wan, Angela Moe	Strategies of survival: Studying the link between women's victimization and offending				http://libpro	2001		295		
Exposure	journalArticle	2013	Wang C.; Sadhavisvam S.; Krekels E.H.J.; Dahan A.; Tibboel D.; Danhof M.; Vinks A.A.; Knibbe C.A.J.	Developmental changes in morphine clearance across the entire paediatric age range are best described by a bodyweight-dependent exponent model	Clinical Drug Investigation	1173-2563	10.1007/s40	http://www	2013	523-534		7	33
Exposure	thesis	2015	Wapinsky, Alisa	Opiate-dependent Pregnant Women's Perceptions of their Personal Strengths, Substance Abuse and Treatment Histories: An Appreciative Inquiry Approach				http://libpro	2015		191		
Outcome	journalArticle	1989	Ward, O. B.; Kopertowski, D. M.; Finnegan, Loretta P.; Sandberg, D. E.	Gender-identity variations in boys prenatally exposed to opiates.	Annals of the New York Academy of Sciences	0077-8923	10.1111/j.17	http://libpro	1989-06	365-366			562
Exposure	journalArticle	1992	Waters L	Pharmacologic strategies for managing pain in children.	Orthopaedic Nursing	0744-6020		http://libpro	1992-01	34-40		1	11
Exposure	journalArticle	2002	Webb, Michael D.; Moore, Paul A.	Sedation for pediatric dental patients.	Dental clinics of North America	0011-8532	0011-8532		2002-10	803-814, xi		4	46
Exposure	thesis	2014	Wedeles, John	Among Pregnant Women in the West Virginia-Smoking Cessation and Reduction in Pregnancy Treatment (SCRIPT) Dissemination Program				http://libpro	2014		109		
Sample Size	journalArticle	2016	Wei L.-C.; Chen C.-T.; Chou S.-Y.; Fan C.-Y.; Liu W.-Y.; Chan H.-Y.	Evaluation of the development of children whose mother is under methadone maintenance treatment during pregnancy	Neuropsychiatry	1758-2016	10.4172/Neu	http://www	2016	161-166		4	6
Outcome	thesis	1998	Weinstein, Diane Weber	Posttraumatic stress disorder, dissociation and substance abuse as long-term sequelae in a population of adult children of substance abusers				http://libpro	1998		203		
Exposure	thesis	2013	Weir, Brian W.	scientific perspectives and multi-level associations among women recently involved in the criminal justice system				http://libpro	2013		232		
Outcome	journalArticle	2003	Weitoft, Gunilla Ringb‰ck; Hjern, Anders; Haglund, Bengt; RosÈn, MÂns	Mortality, severe morbidity, and injury in children living with single parents in Sweden: A population-based study.	The Lancet	0140-6736	10.1016/S01	http://libpro	2003-01	289-295		9354	361
Age	journalArticle	2013	Welle-Strand G.K.; Skurtveit S.; Jones H.E.; Waal H.; Bakstad B.; Bjark˜ L.; Ravndal E.	methadone or buprenorphine: A National Cohort Study of opioid-agonist treatment of Pregnant Women in Norway from 1996 to 2009	Drug and Alcohol Dependence	0376-8716	10.1016/j.dr	http://www	2013	200-206		3-Jan	127
Outcome	thesis	2004	West, Courtney Marie	Substance -abusing women and their roles as mothers: Predicting treatment success in a gender -specific environment				http://libpro	2004		114		
Outcome	thesis	2002	West, Diane D.	Addiction as a search for the sacred				http://libpro	2002		248		
Age	journalArticle	2007	Westrup B.; Sizun J.; Lagercrantz H.	Family-centered developmental supportive care: A holistic and humane approach to reduce stress and pain in neonates	Journal of Perinatology	0743-8346	10.1038/sj.jp	http://www	2007	S12-S18		SUPPL. 1	27
Age	journalArticle	2002	White-Traut R.; Studer T.; Meleedy-Rey P.; Murray P.; Labovsky S.; Kahn J.	Pulse rate and behavioral state correlates after auditory, tactile, visual, and vestibular intervention in drug-exposed neonates	Journal of Perinatology	0743-8346	10.1038/sj/j	http://www	2002	291-299		4	22
Exposure	thesis	1998	White, James Edward	An analytical study on treating drug and alcohol addiction: Evaluating alternative recovery protocols				http://libpro	1998		195		
Outcome	journalArticle	2010	Whitham, J. N.; Spurrier, N. J.; Sawyer, M. G.; Baghurst, P. A.; Taplin, J. E.; White, J. M.; Gordon, A. L.	The effects of prenatal exposure to buprenorphine or methadone on infant visual evoked potentials.	Neurotoxicology and Teratology	0892-0362	10.1016/j.nt	http://libpro	2010	280-288		2	32
Include	thesis	2012	Whitham, Justine N.	Prenatal exposure to buprenorphine or methadone: effects on physical growth, neurological development and temperament in early childhood.				https://digit	2012				

© 2020 Nelson LF et al. *JAMA Network Open.*

Outcome	journalArticle	2015	Whitham, Justine N.; Spurrier, Nicola J.; Baghurst, Peter A.; Weston, Paul; Sawyer, Michael G.	children prenatally exposed to buprenorphine or methadone compared with non-opioid exposed children: The results of a longitudinal study.	Neurotoxicology and teratology	1872-9738 0	10.1016/j.ntt.2015.09.008		2015-12	17-24		Pt A	52
Outcome	thesis	2016	Wiegmann, Wendy Lynn	Impact of residential versus outpatient substance abuse treatment on child welfare outcomes: A secondary analysis of NSCAW II data				http://libpro	2016		77		
Outcome	journalArticle	1995	Wilens, Timothy E.; Biederman, Joseph; Kiely, Kathleen; Bredin, Elizabeth; Spencer, Thomas J.	Pilot study of behavioral and emotional disturbances in the high-risk children of parents with opioid dependence.	Journal of the American Academy of Child & Adolescent Psychiatry	0890-8567	10.1097/000	http://libpro	1995-06	779-785		6	34
Exposure	journalArticle	2005	Wille C.; Bocquet N.; Cojocaru B.; Leis A.; ChÈron G.	Oral morphine administration for children's traumatic pain	Archives de Pediatrie	0929-693X	10.1016/j.ar	http://www	2005	248-253		3	12
Outcome	thesis	2010	Willenbacher, Alison F.	Dominance as an interpersonal construct: Maturity of object relations, attachment style, and measurement				http://libpro	2010		126		
Outcome	thesis	2009	Williams, Karen	The relationship between coping self -efficacy and substance abuse treatment provided to individuals in the New Jersey Drug Court program				http://libpro	2009		175		
Outcome	journalArticle	1975	Wilson G.S.	Somatic growth effects of perinatal addiction.	Addictive diseases	0094-0267		http://www	1975	333-345		2-Jan	2
Exposure	journalArticle	2000	Wilson S.; Easton J.; Lamb K.; Orchardson R.; Casamassimo P.	A retrospective study of chloral hydrate, meperidine, hydroxyzine, and midazolam regimens used to sedate children for dental care.	Pediatric dentistry	0164-1263		http://www	2000	107-112		2	22
Exposure	journalArticle	1990	Wilson TA; Graves SA	Pediatric considerations in a general postanesthesia care unit.	Journal of Post Anesthesia Nursing	0883-9433		http://libpro	1990-02	16-24		1	5
Included	journalArticle	1989	Wilson, G. S.	Clinical studies of infants and children exposed prenatally to heroin.	Annals of the New York Academy of Sciences	0077-8923	0077-8923		1989	183-194			562
Include	journalArticle	1989	Wilson, G. S.	Clinical studies of infants and children exposed prenatally to heroin.	Annals of the New York Academy of Sciences	0077-8923	0077-8923		1989	183-194			562
Outcome	journalArticle	1975	Wilson, G. S.	Somatic growth effects of perinatal addiction.	Addictive diseases	0094-0267	0094-0267		1975	333-345		2-Jan	2
Exposure	journalArticle	1973	Wilson, G. S.; Desmond, M. M.; Verniaud, W. M.	Early development of infants of heroin-addicted mothers.	American journal of diseases of children (1960)	0002-922X	0002-922X		1973-10	457-462		4	126
Age	journalArticle	2009	Reinhold; Rohrmeister, Klaudia; Metz, Verena; Aeschbach Jachmann, Crispa; Thau, Kenneth; Fischer, Gabriele	Association between prenatal tobacco exposure and outcome of neonates born to opioid-maintained mothers. Implications for treatment.	European addiction research	1421-9891 1	10.1159/000216466		2009	150-156		3	15
Outcome	thesis	2017	Witkowski, Lisa	The Mother's Experience: A Phenomenological Study of Maternal Self-Efficacy Among Mothers Attending Outpatient Treatment for Substance Abuse				http://libpro	2017		282		
Outcome	journalArticle	2017	Witt, C. E.; Rudd, K. E.; Bhatraju, P.; Rivara, F. P.; Hawes, S. E.; Weiss, N. S.	Neonatal abstinence syndrome and early childhood morbidity and mortality in Washington state: a retrospective cohort study	Journal of Perinatology	0743-8346	10.1038/jp.2017.106		2017-10	1124-1129		10	37
Outcome	thesis	2003	Wong, Josephine Y.	An exploration of the parenting experiences of mothers living with their young children in a residential drug treatment facility				http://libpro	2003		283		
Exposure	journalArticle	2016	Wood M.E.; Frazier J.A.; Nordeng H.M.E.; Lapane K.L.	childhood neurodevelopmental outcomes: An application of propensity score calibration to adjust for unmeasured confounding by migraine severity	Pharmacoepidemiology and Drug Safety	1099-1557	10.1002/pds	http://www	2016	493-502		5	25
Exposure	thesis	2015	Woolforde, Launette	The relationship of perceived susceptibility, medical mistrust, and perceived racism to HIV screening among adult Black women				http://libpro	2015		300		
Outcome	journalArticle	2010	Wouldes, Trecia A.; Woodward, Lianne J.	Maternal methadone dose during pregnancy and infant clinical outcome.	Neurotoxicology and Teratology	0892-0362	10.1016/j.nt	http://libpro	2010-05	406-413		3	32
Study Type	journalArticle	1994	Yawn B.P.; Thompson L.R.; Lupo V.R.; Googins M.K.; Yawn R.A.	Prenatal drug use in Minneapolis-St Paul, Minn. A 4-year trend.	Archives of family medicine	1063-3987		http://www	1994	520-527		6	3
Study Type	journalArticle	2016	Yim S.W.; Siu L.Y.	Retrospective review of neonatal abstinence syndrome in a regional hospital in Hong Kong	Hong Kong Journal of Paediatrics	1013-9923		http://www	2016	223		3	21
Outcome	journalArticle	2017	Yoo, Sylvia H.; Jansson, Lauren M.; Park, Hee-Jung	Sensorimotor outcomes in children with prenatal exposure to methadone.	Journal of AAPOS : the official publication of the American Association for Pediatric Ophthalmology and Strabismus	1528-3933 1	10.1016/j.jaapos.2017.05.		2017-08	316-321		4	21

© 2020 Nelson LF et al. *JAMA Network Open.*

Study Type	journalArticle	1997	Young, N. K.	Effects of alcohol and other drugs on children	Journal of Psychoactive Drugs	0279-1072			1997-03	23-42		1	29
Outcome	thesis	2004	Zacharias, Jennifer Ann	mothers on children's adjustment: The mediating influences of maternal responsiveness, monitoring, and family rituals				http://libpro	2004		117		
Outcome	journalArticle	2017	Zadzielski E.; Rogers S.; Gargano S.; Washio Y.	ìgroup Prenatal Careî - Providing obstetric care in a community-based substance abuse treatment center	Drug and Alcohol Dependence	0376-8716	10.1016/j.dr	http://www	2017	e221-e222		(Zadzielski E	171
Study Type	journalArticle	1982	Zagon, I. S.; McLaughlin, P. J.; Weaver, D. J.; Zagon, E.	Opiates, endorphins and the developing organism: a comprehensive bibliography.	Neuroscience and biobehavioral reviews	0149-7634 0149-7634			1982	439-479		4	6
Exposure	journalArticle	1992	Zajac J	Pediatric pain management.	Critical Care Nursing Quarterly	0887-9303		http://libpro	1992-08	35-51		2	15
Exposure	journalArticle	2010	Zanette G.; Micaglio M.; Zanette L.; Manani G.; Facco E.	Comparison between ketamine and fentanyl-droperidol for rectal premedication in children: A randomized placebo controlled trial	Journal of Anesthesia	0913-8668	10.1007/s00	http://www	2010	197-203		2	24
Outcome	thesis	2006	Zelazo, Laurel Bidwell	Maternal ego development, substance use and internalizing disorder: Implications for parenting experience and child outcome				http://libpro	2006		91		
Outcome	thesis	2015	Zhai, Zu Wei	The role of attachment to parents in the etiology of substance use disorder				http://libpro	2015		149		
Exposure	journalArticle	2004	Zhirkova I.V.; Stepanenko S.M.; Butyleva O.I.; Zilbert E.V.; Manerova A.F.; Golodenko N.V.	Method of continuous intravenous postoperative analgesia with promedol in newborn children	Anesteziologiia i reanimatologiia	0201-7563		http://www	2004	16-Dec		1	
Exposure	journalArticle	2011	Zhirkova I.V.; Stepanenko S.M.; Kucherov I.I.	[Postoperative analgesia of newborns using local anesthetic administered through wound irrigation catheter of permanent action].	Anesteziologiia i reanimatologiia	0201-7563		http://www	2011	55-58		1	
No Control Group	journalArticle	2000	Ziegler, M.; Poustka, F.; von Loewenich, V.; Englert, E.	[Postpartum risk factors in the development of children born to opiate-addicted mothers; comparison between mothers with and without methadone substitution].	Der Nervenarzt	0028-2804 0028-2804			2000-09	730-736		9	71
Outcome	thesis	2011	Zimmerman, Patricia Amy Freeman	Parenting risk, maternal functioning, and ego development among substance-using mothers				http://libpro	2011		116		
Study Type	journalArticle	1985	Zuckerman B.	Developmental consequences of maternal drug use during pregnancy	NIDA Research Monograph Series	1046-9516		http://www	1985	96-106		(Zuckerman	NO. 59
Study Type	journalArticle	1991	Zuckerman B.; Bresnahan K.	Developmental and behavioral consequences of prenatal drug and alcohol exposure	Pediatric Clinics of North America	0031-3955		http://www	1991	1387-1406		6	38
Study Type	journalArticle	1995	Zuckerman, B.; Frank, D.; Brown, E.	Overview of the effects of abuse and drugs on pregnancy and offspring.	NIDA research monograph	1046-9516 1046-9516			1995	16-38			149
Study Type	bookSection	1993	Zuckerman, Barry; Brown, Elizabeth R.	Maternal substance abuse and infant development.	Handbook of infant mental health.			http://libpro	1993	143-158			
Outcome	journalArticle	2010	Zuckerman, Barry; Kaplan-Sanoff, Margot; Augustyn, Marilyn	Who rules the roost?	Journal of Developmental and Behavioral Pediatrics	0196-206X	10.1097/DBP	http://libpro	2010-01	72-74		1	31
Outcome	journalArticle	2019	[illegible]	[illegible] درمان تعما [illegible] والد کودک بر بهبود شاخص عاطفه در کودکان	Journal of Shaheed Sadoughi University of Medical Sciences & Health Services	2228-5741	10.18502/ss	http://libpro	2019-04	1164-1173		1	27
Outcome	journalArticle	2017		Treatment Option for Opioid-Addicted Infants.	Neonatal Intensive Care	1062-2454		http://libpro	2017	17-20		3	30
Outcome	journalArticle	2015		Statewide Task Force on Prescription Drug Abuse and Newborns Update: Neonatal Abstinence Syndrome Data Summary	The Florida nurse	0015-4199		http://www	2015	14		2	63
Outcome	journalArticle	2013		AAP OKs breastfeeding on methadone but not buprenorphine...American Academy of Pediatrics	Alcoholism & Drug Abuse Weekly	1042-1394		http://libpro	9/2/13	4-Mar		34	25
Outcome	journalArticle	2010		Clinical digest. Methadone exposure in utero puts children's eyesight in jeopardy.	Nursing Standard	0029-6570		http://libpro	6/9/10	17-17		40	24

© 2020 Nelson LF et al. *JAMA Network Open.*

eFigure 1. Downs and Black Quality Assessment of All Included Cohorts

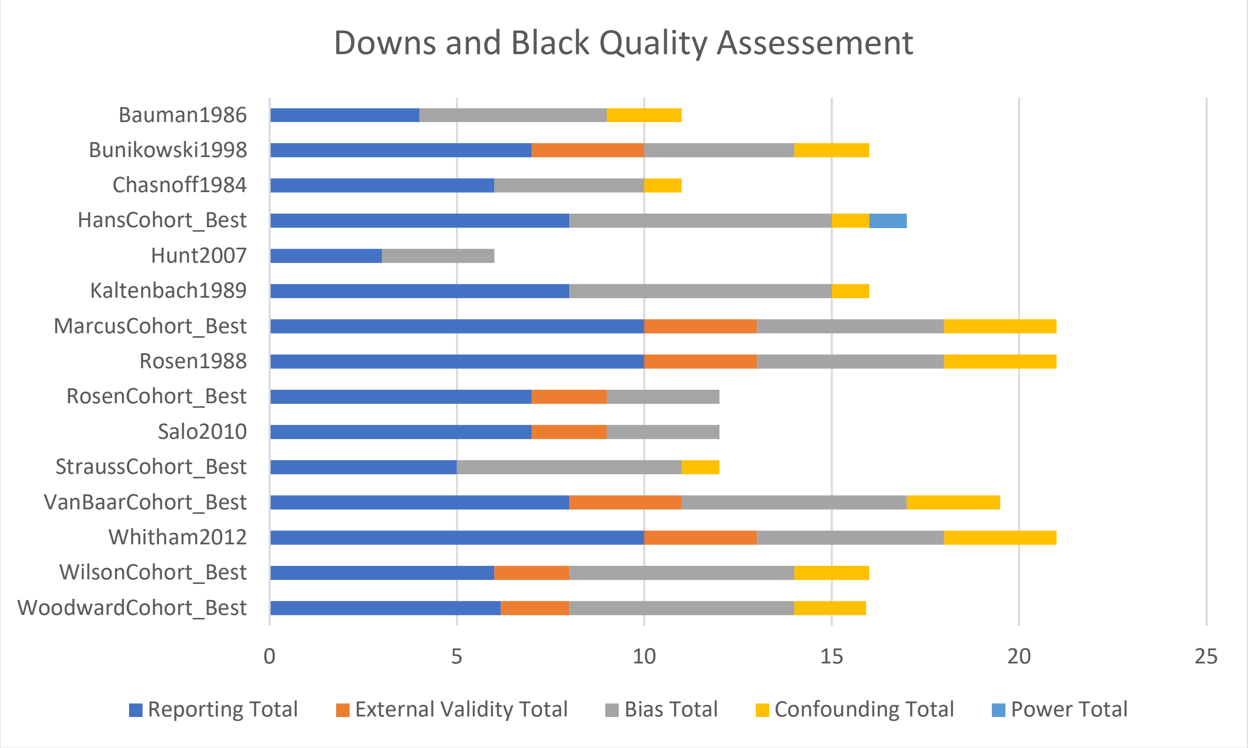

Mean quality score was 15.2. The maximum total points possible was 24.

© 2020 Nelson LF et al. *JAMA Network Open.*

eFigure 2. Publication Bias Funnel Plot

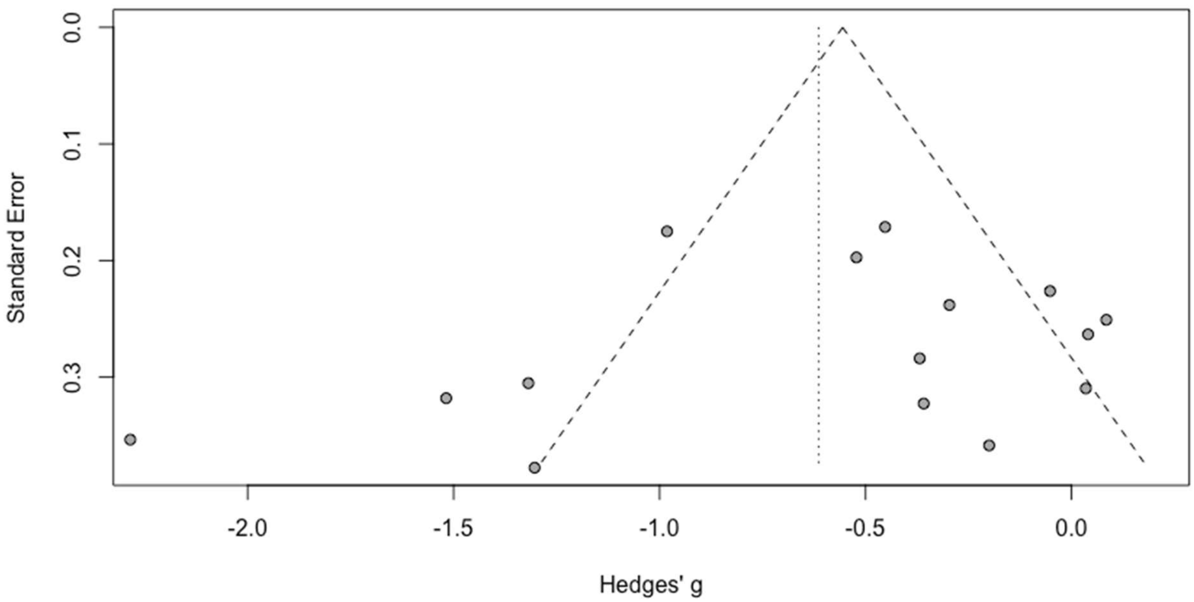

© 2020 Nelson LF et al. *JAMA Network Open.*
